# Female *Drosophila melanogaster* Gene Expression and Mate Choice: The X Chromosome Harbours Candidate Genes Underlying Sexual Isolation

**DOI:** 10.1371/journal.pone.0017358

**Published:** 2011-02-28

**Authors:** Richard I. Bailey, Paolo Innocenti, Edward H. Morrow, Urban Friberg, Anna Qvarnström

**Affiliations:** Department of Ecology and Genetics, Evolutionary Biology Centre (EBC), Uppsala University, Uppsala, Sweden; Montreal Botanical Garden, Canada

## Abstract

**Background:**

The evolution of female choice mechanisms favouring males of their own kind is considered a crucial step during the early stages of speciation. However, although the genomics of mate choice may influence both the likelihood and speed of speciation, the identity and location of genes underlying assortative mating remain largely unknown.

**Methods and Findings:**

We used mate choice experiments and gene expression analysis of female *Drosophila melanogaster* to examine three key components influencing speciation. We show that the 1,498 genes in Zimbabwean female *D. melanogaster* whose expression levels differ when mating with more (Zimbabwean) versus less (Cosmopolitan strain) preferred males include many with high expression in the central nervous system and ovaries, are disproportionately X-linked and form a number of clusters with low recombination distance. Significant involvement of the brain and ovaries is consistent with the action of a combination of pre- and postcopulatory female choice mechanisms, while sex linkage and clustering of genes lead to high potential evolutionary rate and sheltering against the homogenizing effects of gene exchange between populations.

**Conclusion:**

Taken together our results imply favourable genomic conditions for the evolution of reproductive isolation through mate choice in Zimbabwean *D. melanogaster* and suggest that mate choice may, in general, act as an even more important engine of speciation than previously realized.

## Introduction

The evolution of sexual isolation during speciation depends on a joint change in male sexual traits and female preference for those traits [1]. Theoretical work has identified several genetic conditions favouring this process, such as sex linkage and spatial clustering of genes underlying species-specific sexual signalling systems, [2]–[5]. Our empirical knowledge of the genetics underlying male secondary sexual traits is increasing [4], but the genetics underlying female choice mechanisms, causing biases in male fertilization success [6], remain largely unexplored. Sex linkage of the genes underlying female choice mechanisms should lead to increased potential rate of sequence divergence in response to selection [7] and favours processes such as reinforcement [3] and good genes sexual selection [2], but not Fisherian runaway selection [2]. The spatial clustering of mate choice genes affects inter-taxon recombination during periods of contact and gene exchange and can mitigate the homogenising effects of gene flow [5]. Therefore taxa whose female choice genes are more sex-linked and/or more highly clustered are expected to be more prone to speciation, other things being equal.

**Figure 1:**
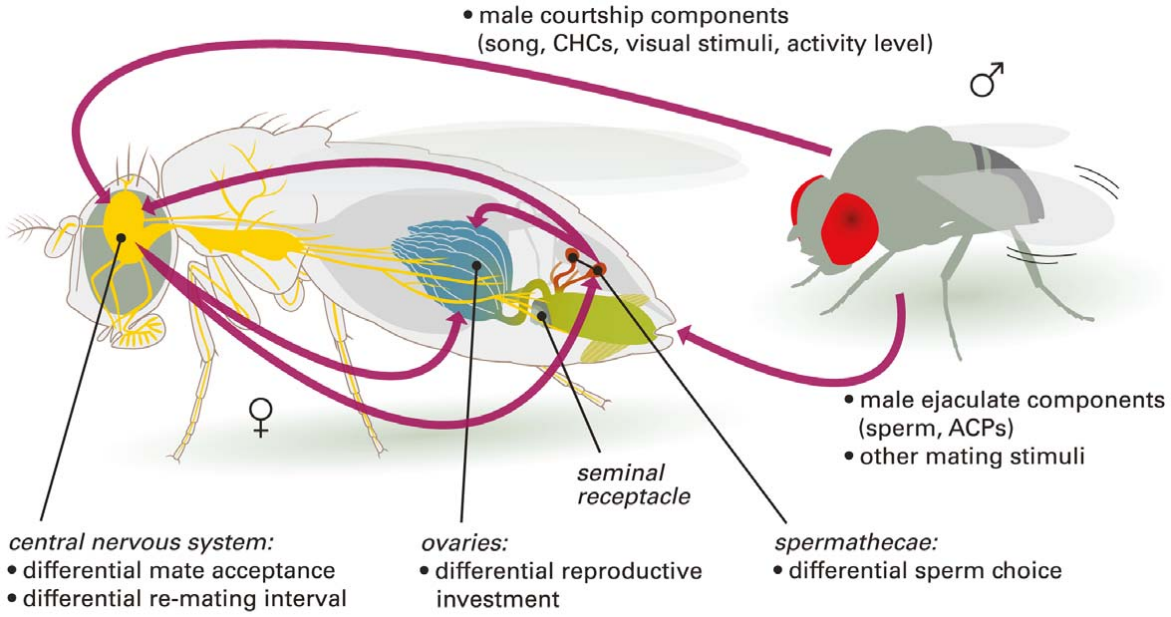
Mechanisms of mate choice influencing sexual isolation. Mate choice is any bias in male reproductive success caused by female responses (active or passive) to phenotypic differences between males (6; Text S1). The labelled female tissues (seminal receptacle not tested for overrepresentation of candidate mate choice genes; green  =  other untested parts of the reproductive tract) are possible locations for mechanisms of mate choice (bullet points). The arrows represent routes by which mate choice may occur. Active female choice is represented by arrows starting or finishing at the female brain; passive female choice by any arrows that do not involve the female brain. Arrows between tissues within the female represent neuronal and/or hormonal responses. The digestive tract, containing the majority of the remainder of the tested tissues, is represented in dark grey. ACPs  =  accessory gland proteins; CHCs  =  cuticular hydrocarbons.

Biases in male reproductive success may be caused by multiple female choice components (Fig. 1; Text S1) that together can have an overriding influence on reproductive isolation between populations [8]. From a mechanistic point of view, female mate choice is the product of the interplay between neurological and physiological processes, which in turn are regulated by gene expression patterns during courtship and mating. An important step in understanding the link between mate choice and speciation is therefore to understand how female gene expression patterns affect reproductive isolation. Gene expression studies do not rely on pre-existing genetic divergence to reveal mechanistic associations between genes and traits, making them ideal for identifying genes that are potential targets for future divergence. Establishing the identity of the genes underlying plastic female responses to males belonging to their own population versus other populations can be used to make predictions of (i) the potential for future divergence, (ii) the rate by which divergence may proceed, and (iii) what evolutionary processes are likely to be driving their evolution.

*Drosophila melanogaster* from outside sub-Saharan Africa (cosmopolitan or M strain) are thought to have diverged from southern African strains during their spread around the world as human commensals within the last 10,000 years [9]–[11]. They are genetically depauperate and the majority of their genetic variation is thought to be a subset of that found in Africa [12], [13], although a number of fixed differences exist (10) and mean *F*_ST_ between European M strain and Zimbabwean (Z strain) *D. melanogaster* is 0.23 [14]. Lineages such as these that are at an early stage of evolving reproductive isolation may serve as important model systems for studying speciation through divergence in sexual signalling systems. There is partial reproductive isolation between populations of *Drosophila melanogaster* from Zimbabwe (Z strain) and from the rest of the world (M strain) [15]–[17]; M strain females show no apparent pre-copulatory preferences for M males but Z strain females prefer Z males. Sperm-egg incompatibilities also exist when females from ‘strong Z’ isofemale lines (those with strong sexual preference for Z males) mate with M strain males, but not vice versa [18]. In this study we use mate choice experiments and gene expression analysis in Z strain female *D. melanogaster* to examine three key components influencing speciation: (i) which of the known mate choice mechanisms in *Drosophila* are likely to be involved in Z female discrimination against M males (i.e. that play a role in relation to sexual isolation), (ii) the degree of sex linkage of the candidate mate choice genes involved in sexual isolation; and (iii) the physical clustering of these genes. Thus, we view female discrimination of males belonging to other populations than their own as a composite trait (i.e. the result of a joint action of several mate choice mechanisms) and use gene expression analysis to establish a candidate set of genes underlying this composite trait. The candidate set of genes is subsequently used to evaluate the potential for evolution of stronger sexual isolation (i.e. stronger discrimination against males not belonging to their own population).

By identifying genes differentially expressed between Z strain females mated to preferred Z males versus less preferred M males, we find that (i) tissue specificity patterns of the identified candidate genes indicate the action of multiple mate choice mechanisms involved in sexual isolation, (ii) candidate mate choice genes involved in sexual isolation cluster disproportionately on the X chromosome, and (iii) they form several tight physical clusters on the X chromosome and the autosomes. These conditions are expected to lead to faster evolution than would be the case with few genes involved in mate choice and little X linkage, plus a greater possibility for divergence in sympatry in certain genomic regions. We therefore conclude that mate choice may act as an even more powerful engine of speciation than previously realized.

## Results

### Female mate choice in Z and M strains

Using a multi-choice mating design allowing separate estimation of mate choice and mating propensity [19], [20], we confirm a mate preference of Z females for Z males in two individual ‘strong Z’ lines and one composite line (SZ) made up of six strong Z isofemale lines (Table 1). We also show that, in the best-fitting statistical model, both sexes of M strain have more than four times the mating propensity of Z strain. Hence M strain males court more vigorously, and M strain females have lower resistance to courtship. Furthermore, we find that a model of asymmetric preference of both Z and M females for Z males provides the best fit to the data (Table 1). The composite line (SZ) with confirmed discrimination against M males was subsequently used in the gene expression analysis.

**Table 1:** Multi-choice mate preference tests.

Model	Likelihood	Deviance	Parameters	AIC	*I* Z	*I* M	*MP* male	*MP* female
**Asymmetric female preference + ** ***MP*** ** sexes combined**	**−279.64**	**559.27**	**2**	**563.27**	**0.43**	**0.43**	**4.77**	**4.77**
**Symmetric isolation + ** ***MP***	**−278.69**	**557.37**	**3**	**563.37**	**0.23**	**0.23**	**1.53**	**5.7**
**Asymmetric female preference + ** ***MP***	**−278.69**	**557.37**	**3**	**563.37**	**0.32**	**0.32**	**3.27**	**5.7**
**Z female preference only + ** ***MP***	**−278.69**	**557.39**	**3**	**563.39**	**0.26**	**0**	**2.02**	**5.74**
Male isolation only	−284.58	569.16	2	573.16				
Z female preference only + *MP* sexes combined	−289.92	579.83	2	583.83				
*MP* only	−292.81	585.62	2	589.62				
Asymmetric male preference + *MP* sexes combined	−297.32	594.63	2	598.63				
Asymmetric male preference only	−298.7	597.4	1	599.4				
Symmetric isolation + *MP* sexes combined	−308.31	616.62	2	620.62				
Symmetric isolation only	−352.41	704.82	1	706.82				
Symmetric female isolation only	−351.51	703.02	2	707.02				
Asymmetric female preference only	−365.63	731.27	1	733.27				
Random mating	−371.53	743.05	0	743.05				

*I*  =  isolation/preference index for Z strain or M strain; *MP*  =  mating propensity. Model comparisons of multi-choice mate preference tests ranked by AIC (best-fitting model at the top). Models in bold represent the candidate set that provide a good fit to the data (AIC within 2 of the best model), and only parameter estimates for these models are presented.

### Differentially expressed genes

To elucidate potential mechanisms of sexual isolation through mate choice, we identified candidate female choice genes by examining gene expression in Zimbabwean SZ female *D. melanogaster* 30 minutes after mating with more (their own strain) versus less (M strain) preferred males. At 10% false discovery rate (i.e. up to 10% of genes are expected to be false positives) 1,498 genes were differentially expressed between ZxZ and ZxM matings (Table S1). These represent the set of candidate genes whose expression level is associated with mate choice discrimination between M and Z males. Given that mechanisms of mate choice in *Drosophila* may include differential re-mating interval, egg production and sperm storage and manipulation (Fig. 1; Text S1) on top of pre-copulatory female preference, we expected genes with high expression in the central nervous system, the ovaries, and the sperm storage organs to be overrepresented among the candidate mate choice genes involved in sexual isolation. For a total of fifteen different adult tissues (Table S2), Fisher exact tests were carried out to examine overrepresentation of genes with double the expression level in that tissue compared to whole flies (‘tissue-enriched expression’) among the 1,498 differentially expressed genes. Genes with tissue-enriched expression were heavily underrepresented for most tissue types (Table S2), including both mated and virgin spermathecae; however there were more ovary-enriched and brain-enriched genes than expected by chance and high but non-significant numbers of thoracic abdominal ganglion-enriched genes, a second component of the central nervous system (Table S2).

### Sex linkage of candidate mate choice genes

**Figure 2:**
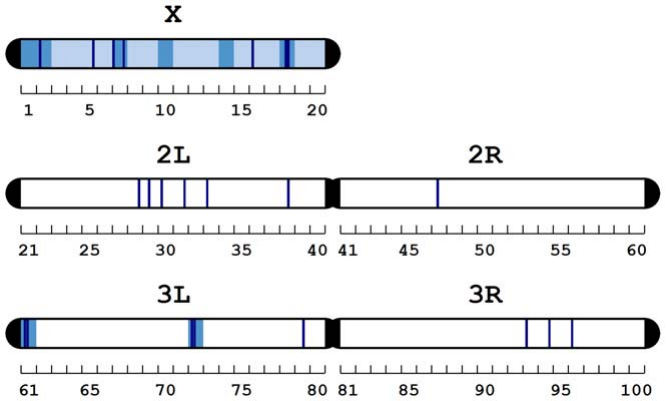
Non-random distribution of candidate mate choice genes. Significant clustering on the X chromosome is represented by pale blue, on the cytobands (1–100, shown on the labels beneath each chromosome) by medium blue, and on sub-bands (A–F, not labelled) by dark blue. There are a further 67 significant sub-sub-bands not represented (see Table S3).

Traits associated with sexual isolation are predicted to be largely determined by sex-linked genes [4]. Existing QTL analyses using recombinant lines between ‘strong Z’ isofemale lines and M strain revealed that all chromosomes contribute to the stronger mate preferences of Z females compared to M. However, the strongest effects were on the autosomes, with a particularly strong effect of the tip of the left arm of chromosome III [21], [22]. In contrast to these previous studies, our study focuses on candidate genes underlying Z females overall responses to being courted and mated to males belonging to the Z or M lineages. We found that a disproportionately large number of our candidate mate choice genes were present on the X chromosome (Fig. 2; Table S3). If stronger discrimination were favoured by selection, the prediction based on this finding is more rapid future divergence than if the genes were spread evenly across chromosomes.

### Gene clustering and recombination

Hypergeometric tests were used to identify chromosomal cytobands holding more candidate mate choice genes than expected by chance. Cytobands are the unique banding patterns of each chromosome that become visible microscopically after staining [23]. As expected, a greater number of clusters of high concentrations of candidate mate choice genes occur on the X chromosome than on the autosomes (Fig. 2; Table S3). Despite its smaller size, the X chromosome holds 16 cytobands and sub-bands with an overrepresentation of candidate mate choice genes, compared to 8 on chromosome II and 10 on chromosome III. However, the tip of the left arm of chromosome III, which exerted a strong influence on mate choice patterns in previous studies using recombinant lines [22], also contains high concentrations of candidate mate choice genes in this study (Fig. 2; Table S3). This suggests that some of the candidate genes identified here, or their regulatory regions, may represent the QTL identified previously.

## Discussion

We identify a candidate set of mate choice genes whose expression levels in female Z strain *D. melanogaster* are affected by mating with males from their own preferred population versus those from another (M strain) less preferred population. Genes with enriched expression in the central nervous system and ovaries are overrepresented among the candidate gene set. These genes are also disproportionately X-linked and form a number of tight physical clusters, mainly on the X chromosome but also on the autosomes.

In the context of speciation, gene expression studies are often used to investigate the genomics underlying phenotypic differences between lineages [24], [25]. We have used a different approach by instead comparing two groups of females belonging to the same lineage (the Z strain) that were exposed to males belonging to two different lineages during courtship and mating. Our main assumption is that the observed differences in gene expression patterns between these two groups of females should mainly be caused by differences in their neurological and physiological responses to these two types of males. The differences therefore represent variation in the plastic responses made by females after exposure to males of two different genotypes. With the identified candidate set of genes we can then make predictions regarding the likelihood of evolution of even stronger differences in Z females' overall responses to males belonging to their own population versus the M strain. However, at this stage we cannot disentangle the role of individual choice mechanisms nor can we link particular responses to particular genes. Still, tissue specificity of the differently expressed genes can give us an idea regarding the most likely type of mate choice mechanisms involved.

Genes with ovary-enriched and central nervous system (particularly the brain)-enriched expression were heavily overrepresented among candidate genes, while genes with enriched expression in all other tissue types tested were heavily underrepresented. This suggests that, as well as the differences in pre-copulatory female choice in favour of males belonging to their own populations described here and previously [15]–[17], phenotypic differences between males may also have led to discrimination in terms of cryptic female choice mechanisms through differential egg and ovary development, and possibly interactions between active and cryptic choice (Fig. 1; Text S1). The lack of overrepresentation of spermatheca-enriched genes may indicate the absence of a role for differential sperm storage and manipulation in mate choice. However, tissue-level gene expression data were not available for other reproductive organs bearing sperm and their potential role cannot be discounted (see ‘Spermathecae as the site of sperm storage and manipulation’ in Text S1). That the combined mate choice mechanisms behind sexual isolation together represent a highly polygenic composite trait, including several gene clusters on the X chromosome, means that there are many possible and potentially rapid pathways to the evolution of stronger isolation.

### Sex linkage

Despite the X chromosome only representing 16% of the genome, 21.2% of candidate mate choice genes were X-linked. Establishing the genomic location of the genes underlying female choice of their own type of males provides an excellent tool for evaluating the likelihood of evolution of complete sexual isolation. Quantifying the relative influence of genes on the X chromosome is particularly useful because X-linked genes tend to diverge fast, due to the higher mutation accumulation and greater exposure to selection caused by hemizygosity [7]. In general, sex-linked genes are expected to contribute progressively more to heritable differences between taxa as time passes because initial selection on standing genetic variation favours divergence on autosomes [26], whereas divergence due to new mutations will be biased towards sex chromosomes [4], [7], and this divergence accumulates over time. Hence even if choice mechanisms are mainly determined by sex-linked genes, the autosomal genes involved may nevertheless diverge first between populations. The extent by which mate choice divergence is attributed to evolution of sex-linked genes should therefore increase with time since divergence between populations. Differences between gene expression studies and studies using recombinant lines can be interpreted in the light of this prediction. Our gene expression study was designed to detect genes with a mechanistic involvement in sexual isolation, rather than focusing on genes that have already diverged between strains. This approach allows not only the detection of choice genes that have themselves diverged - e.g. the genes or their *cis*-regulatory regions located at the tip of the left arm of chromosome III, as also detected in previous QTL studies [22] - but also the detection of mate choice genes that are candidates for future divergence. We can therefore not only make conclusions regarding the rate of previous evolution but also evaluate how rapidly divergence in mate choice genes can proceed, based on their degree of sex linkage, and hence predict an important role of mate choice mechanisms in the future build-up of reproductive isolation.

### Sex linkage and recombination

Concentration of reproductive isolation genes within regions of low inter-taxon recombination through physical clustering [27] or presence in fixed chromosomal inversions [28] enhances the maintenance of distinct genotypes during periods of gene exchange. The observed disproportionate sex linkage has two effects related to recombination. Firstly, the non-random distribution of mate choice genes among chromosomes increases the number of clusters of these genes found in close physical proximity. Secondly, sex linkage increases the possibility of linkage with sexual signal and genetic incompatibility genes, which are often sex-linked [4], [27]. Taken together, the genomic conditions appear ideal for increased evolution of sexual isolation in Zimbabwean female *D. melanogaster*.

One previous (intraspecific) study has examined female gene expression associated with male attractiveness, in the fish *Xiphophorus nigrensis* [29], but they did not present the chromosomal distribution of female mate choice gene expression patterns or link these to sexual isolation. Two previous studies have found evidence for sex-linked differences in female choice between diverging taxa based on inheritance patterns [27], [30], but no candidate mate choice genes were established. In contrast to our study, these previous studies were on taxa – birds [27] and butterflies [30] - in which females are the heterogametic sex; a factor thought to increase the influence of sex-linked genes on the early evolution of prezygotic barriers to gene exchange [4].

Our study reveals novel insights into the present and potential future influence of mate choice on speciation in Zimbabwean *D. melanogaster*. Studies such as this, identifying a set of candidate genes with a mechanistic involvement in mate choice, can aid in predicting the likelihood and rate of speciation based on mate choice divergence, and also the influence of mate choice relative to other isolating mechanisms as speciation progresses. Drawing firm conclusions regarding the importance for speciation of individual genes in this candidate set will require further examination of their influence on pre- or postcopulatory isolation and on their responses to specific male stimuli. However, coincidence of these candidate mate choice genes with mate choice QTL provides one means of homing in on the individual genes (or their *cis*-regulatory regions) that currently cause sexual isolation.

Some mate choice mechanisms may be more sex-linked than others, and this is likely to affect their relative contributions to the build-up of reproductive isolation. Mate choice based on pre-existing sensory biases – likely to have evolved to aid in survival and to have equal fitness in both sexes – is expected to have a strong autosomal component [4], but mate choice mechanisms involving sexually antagonistic traits (such as cryptic choice involving primary reproductive organs) are more likely to be sex-linked [4]. Genomic approaches may therefore shed novel light on the consecutive role of different mate choice mechanisms in the speciation process.

## Materials and Methods

### Fly culturing and experimental conditions

The outbred strain LH_M_ from California [31] represented M strain flies. Z strain flies were represented by the ‘strong Z’ isofemale lines ZS2 and ZS53, plus a composite ‘strong Z’ line, ‘SZ’. All flies were maintained using the standard protocol for LH_M_ [31]. Flies were reared on standard cornmeal/yeast medium and kept on a 12 h: 12 h light: dark cycle. Rearing and experimentation were carried out at 25°C and 60% relative humidity. All flies were virgin and 7 days post-eclosion on the day of experimentation. The composite line SZ was produced by crossing 6 strong Z lines from Sengwa and Harare [15] (ZS2, ZS11, ZS53, ZS56, ZH12, ZH32). Lines were crossed sequentially for 2 generations before being thoroughly mixed to produce a single population, which was then maintained as a 14-vial culture (population size  =  448).

### Multi-choice mating experiments

Six replicate multi-choice mating experiments were carried out to assess the strength of mate preferences and mating propensity, and to distinguish between assortative versus unidirectional female preferences. Each trial involved four individuals: a male and female LH_M_ and a male and a female of either SZ or one of the two strong Z isofemale lines. The isofemale lines ZS2 and ZS53 were used in 4 replicates, and SZ was used in two. The first mating only was recorded in each trial. Each trial of multi-choice assortative mating experiments lasted a maximum of 2 hours. Marking on the thorax under CO_2_ anaesthesia with acrylic paint mixed with water was alternated between strains. Using pooled data, estimation of the assortative mating index (*I*) and mating propensity followed Bailey et al [20], but with AIC used to choose between models. Additionally, asymmetric preference was tested by assuming both individuals of one sex equally preferred males of one strain or the other and using *I* to indicate the strength of asymmetric preference.

### Gene expression analysis

To identify genes whose expression levels shortly after mating differed between Z females mated to Z males versus Z females mated to M males, eight SZ females were individually mated to LH_M_ and eight to SZ males in each of four replicates, and subsequently flash-frozen in liquid nitrogen 30 minutes after copulation had ceased. While differences in gene expression between the period of courtship and postmating are likely [32], these changes are not instantaneous and instead build in magnitude over a period of 6 hours [33]. We therefore expect that gene expression 30 minutes postmating will mainly capture changes occurring during courtship and mating. There were 4 replicates x 2 treatments (ZxZ and ZxM)  =  8 samples involving a total of 64 Z strain female flies. Frozen flies were stored at -80°C until RNA extraction. Extractions were carried out using whole flies, no more than 2 days after freezing. Total RNA was extracted using Trizol (Invitrogen, Carlsbad, CA, USA) and purified with an RNeasy Mini Kit (Qiagen, Hilden, Germany). RNA quantity and quality was checked with an Agilent Bioanalyzer (Agilent Technologies, Santa Clara, CA, USA). According to the manufacturer's instructions, samples were prepared and hybridized to Affymetrix GeneChip Drosophila Genome 2.0 (Affymetrix, Santa Clara, CA, USA) by the Uppsala Array Platform (Uppsala, Sweden). Each experimental treatment consisted of four independent RNA extractions and hybridizations, giving a total of 8 arrays.

Gene expression data were analyzed using R 2.8.1 [34] and BioConductor 2.3 [35]. Background correction, between-array normalization and transformation were carried out using the VSN method for variance stabilization and calibration of microarray data. Summarization employed the Robust Multichip Average (RMA) algorithm as implemented in the Affy package. Filtering involved removal of probe sets with no Entrez Gene ID annotation and, where multiple probe sets mapped to the same Entrez Gene ID, the probe set with the largest variance across samples was retained and others removed. Probe sets showing low variability (variance interquartile range <0.5) were also removed. To account for differences in reliability between individual arrays, each array was weighted according to how well its expression values followed the linear model using the REML scoring method. Differential expression between Z females mated with Z versus M males was then assessed using a Student’s *t*-test and an empirical Bayes method to moderate standard errors of the estimated log fold change, as implemented in the LIMMA package. Analyses were carried out assuming an adjusted *P* value (*q*) giving an FDR (false discovery rate) of either 5% or 10%. No genes had lower than 5% FDR; hence results for 10% FDR are reported.

### Tissue bias among differentially expressed genes

Tissue-level bias in expression of the candidate mate choice gene set was tested for adult brain, head, eye, thoracic abdominal ganglion, salivary gland, crop, midgut, tubule, hindgut, heart, fat body, ovary, virgin spermathacae, mated spermathecae, and carcass. For each tissue the number of the 1,498 differentially expressed genes in that tissue with at least 200% the expression level of whole flies (mRNA enrichment; expression level values downloaded directly from http://www.flyatlas.org [36], [37]) was counted and tested against the expected number from the filtered data set (6,535 genes) using one-tailed Fisher exact tests (*p*<0.05) in R 2.10.1 [34].

### Non-random chromosomal distribution of differentially expressed genes

To find chromosomes and chromosomal regions enriched for candidate mate choice genes, unconditional hypergeometric tests for overrepresentation on chromosomal cytobands were carried out (*p*<0.05; Category package in R, modified [38]). The unfiltered data set, with unmapped genes removed, was used to generate expected values.

## Supporting Information

Text S1 Mechanisms of mate choice: male traits, female responses and the evolution of sexual isolation.
(DOC)

Table S1 Annotation of differentially expressed genes. Affymetrix probeset and gene annotation for each transcript differentially expressed between Z strain females mated with Z strain males versus M strain males.
(PDF)

Table S2 Tissue-level enrichment analysis. Tissues with an overrepresentation of differentially expressed genes with at least 200% the expression level in whole flies (P<0.05) are highlighted in bold.
(PDF)

Table S3 Results of non-random chromosomal distribution analysis, including all significant cytobands, sub-bands and sub-sub bands (p<0.05).
(PDF)
