# Supplementary material for: Female Drosophila melanogaster Gene Expression and Mate Choice: The X Chromosome Harbours Candidate Genes Underlying Sexual Isolation
Source: PLoS One. 2011 Feb 28;6(2):e17358. doi: 10.1371/journal.pone.0017358 (PMC3046225; doi:10.1371/journal.pone.0017358)
Supplement: Text S1 — Mechanisms of mate choice: male traits, female responses and the evolution of sexual isolation. (DOC) [file pone.0017358.s001.doc]

**Supporting Information**

Bailey et al.

**S1 text**

**Mechanisms of mate choice: male traits, female responses and the evolution of sexual isolation.** Mechanisms of mate choice have been studied in great detail in *D. melanogaster.* Traits of male *D. melanogaster* influencing female mate choice include courtship song (1), courtship vigour (2), visual courtship display behaviours, cuticular hydrocarbons (CHCs) (3), mechanical stimuli during mating, and components of the ejaculate (sperm, accessory gland proteins (4))*.* The pathways (male signals and corresponding female choice mechanisms) illustrated in Fig 1 have been shown to occur in *D. melanogaster* or other species and include both active choice in the form of behavioural acceptance or rejection to copulate as well as a number of mechanisms referred to as “cryptic” female choice. (One mechanism of mate choice not represented in the figure is the male’s ability to alter the female’s CHC profile (3), which may for example alter the degree to which female CHCs stimulate male courtship). The extent to which the illustrated pathways of mate choice (Fig 1) also function in the context of sexual isolation depends on the degree of population divergence in male traits and/or in female choice mechanisms. Since variation in many of the relevant male traits is likely to be larger between than within populations we expect a combination of pathways to contribute to sexual isolation. Female behavioural acceptance/rejection to copulate is a well-studied major contributor to sexual isolation. A number of “cryptic” female mate choice mechanisms have also been proven important in the context of sexual isolation, including conspecific sperm precedence (5); reduced fertility of females in heterospecific crosses due to selective sperm storage (5, 6); and male failure to stimulate ovulation (6, 7) or egg production (8). A number of intraspecific studies have revealed differential female investment in offspring quality/quantity based on male attractiveness (9), while a reduction in nestling provisioning by female parents (not represented in Fig 1, but also a mechanism of mate choice via differential investment in offspring) has been shown in Gouldian finch females mated with males of the partially genetically incompatible alternative colour morph (10).

***Drosophila melanogaster* male accessory gland proteins and mate choice.** In *D. melanogaster* there is definitive evidence that the male sex peptide Acp70A, passed to females in the ejaculate during copulation, reduces female post-mating receptivity and causes oviposition (refractory behaviour). Studies of SPR (also known as CG16752), the female receptor for Acp70A, reveal that Acp70A passes from the female reproductive tract to the central nervous system via the haemolymph, and causes female refractory behaviour by modulating activity of *fru* neurons in the brain (11). This particular pathway of sexual selection appears therefore to be a candidate for causing sexual isolation between diverging populations. However the structure and function of Acp70A is highly conserved across insects (11) allowing little scope for direct quantitative variation in its effects, although other male or female factors may cause quantitative variation in the effectiveness of Acp70A. Furthermore, expression of SPR did not differ significantly between treatments in our experiment.

**Spermathecae as the site of sperm storage and manipulation.** While the spermathecae are the primary tissues used for long-term sperm storage in *D. melanogaster*, about twice as much sperm can be stored in a separate tissue, the seminal receptacle (Fig 1) (12). Furthermore, there is also potential for sperm manipulation in the vagina (Fig 1). Therefore, despite finding no evidence in this gene expression study to suggest mate choice in Z strain female *D. melanogaster* involves differential sperm use, there is plenty of scope for this still to occur in other tissues.

**References**

1. Colegrave N, Hollocher H, Hinton K, Ritchie MG (2000) The courtship song of African *Drosophila melanogaster*. *J Evolution Biol* 13:143-150.

2. Coyne JA, Elwyn S (2006) Does the desaturase-2 locus in *Drosophila melanogaster* cause adaptation and sexual isolation? *Evolution* 60:279-291.

3. Everaerts C, Farine J-P, Cobb M, Ferveur J-F (2010) *Drosophila* Cuticular Hydrocarbons Revisited: Mating Status Alters Cuticular Profiles. *PLoS ONE* 5:e9607. doi:10.1371/journal.pone.0009607.

4. Ram KR, Wolfner MF (2007) Seminal influences: *Drosophila* Acps and the molecular interplay between males and females during reproduction. *Integr Comp Biol* 47:427-455.

5.Howard DJ (1999) Conspecific sperm and pollen precedence and speciation. *Annu Rev Ecol Syst* 30:109-132.

6. Markow TA (1997) Assortative fertilization in *Drosophila*. *P Natl Acad Sci USA* 94:7756-7760.

7. Price CSC, Kim CH, Gronlund CJ, Coyne JA (2001) Cryptic reproductive isolation in the *Drosophila simulans* species complex. *Evolution* 55:81-92.

8. Nilsson T, Fricke C, Arnqvist G (2002) Patterns of divergence in the effects of mating on female reproductive performance in flour beetles. *Evolution* 56:111-120.

9. Sheldon BC (2000) Differential allocation: tests, mechanisms and implications. *Trends Ecol Evol* 15:397-402.

10. Pryke SR, Griffith SC (2010) Maternal adjustment of parental effort in relation to mate compatibility affects offspring development. *Behav Ecol* 21:226-232.

11. Yapici N, Kim YJ, Ribeiro C, Dickson BJ (2008) A receptor that mediates the post-mating switch in *Drosophila* reproductive behaviour. *Nature* 451:33-38.

12. Pitnick S, Markow T, Spicer GS (1999) Evolution of multiple kinds of sperm storage organs in *Drosophila*. *Evolution* 53:1804-1822.
