# Supplementary material for: Female Drosophila melanogaster Gene Expression and Mate Choice: The X Chromosome Harbours Candidate Genes Underlying Sexual Isolation
Source: PLoS One. 2011 Feb 28;6(2):e17358. doi: 10.1371/journal.pone.0017358 (PMC3046225; doi:10.1371/journal.pone.0017358)
Supplement: Table S1 — Annotation of differentially expressed genes. Affymetrix probeset and gene annotation for each transcript differentially expressed between Z strain females mated with Z strain males versus M strain males. (PDF) [file pone.0017358.s002.pdf]

Probe	Symbol	Chromosome	Gene	Cytoband	UniGene	logFC
1634092_at	CG3654	3L	39103	67B9-67B1(	Dm.29618	-0,361068
1625270_at	CG31259	3R	41010	85A2-85A2	Dm.6169	-0,415024
1635854_s.	CG40351	3L	3354971		Dm.5195	-0,331018
1625445_s.	shn	2R	36171	47D6-47D7	Dm.4730	-0,366795
1629184_at	CG3819	3L	40069	75E6-75E6	Dm.13243	-0,372508
1626257_at	CG13656	3R	43047	96C8-96C8	Dm.16495	0,303284
1624378_at	east	X	46006	2C2-2C4	Dm.6506	-0,322882
1633465_at	Cp7Fa	X	3885650	7F1-7F1	Dm.23618	-0,966286
1631343_at	CG15571	X	31365	4B3-4B3	Dm.28015	-0,793428
1630441_at	CG12716	X	32248	11D5-11D5	Dm.25386	-0,743974
1636174_at	GstD9	3R	41502	87B8-87B8	Dm.21658	-0,763961
1634154_at	CG15570	X	31366	4B3-4B3	Dm.26454	-0,583006
1629407_at	CG13113	2L	34271	30B10-30B1	Dm.26770	-0,579673
1632992_at	CG11381	X	31067	1E4-1E4	Dm.5813	-0,560702
1627551_s.	AttA	2R	36636	51C1-51C1	Dm.3981	-0,514829
1632975_at	CG4398	2R	36838	53C4-53C4	Dm.16005	-0,510924
1637707_at	CG4009	3R	42054	89E10-89E1	Dm.27320	-0,446913
1624882_at	CG4066	3R	41493	87B6-87B6	Dm.27269	-0,461653
1626086_at	CG10621	2L	35152	37B7-37B7	Dm.10781	0,420911
1631394_at	CG31324	3R	43177	96F14-97A1	Dm.20262	-0,47411
1636343_at	CG6283	3R	43250	97D14-97D'	Dm.5888	-0,385738
1631481_a.	Kr-h1	2L	33861	26B5-26B5	Dm.3266	-0,441636
1637144_a.	Map205	3R	43765	100E3-100E	Dm.1892	-0,400976
1630550_a.	CG5674	2L	35072	36E3-36E3	Dm.12153	-0,403664
1629229_a.	srp	3R	41944	89A11-89A'	Dm.1934	-0,425425
1637182_at	CG9503	X	32424	13A1-13A1	Dm.11854	-0,381957
1634762_a.	CG15096	2R	37170	55F5-55F5	Dm.3842	-0,421049
1633208_at	CG11741	3R	5740784		Dm.33388	-0,448344
1638176_at	Magi	2R	37403	57C2-57C3	Dm.714	-0,320632
1637835_at	CG12177	X	32326	12B2-12B2	Dm.17450	-0,381129
1639320_a.	Ddc	2L	35190	37C1-37C1	Dm.12979	-0,383241
1631779_s.	ptr	X	44529	3A1-3A1	Dm.1828	-0,402564
1639550_a.	CG9650	X	31660	7A2-7A5	Dm.20992	-0,387743
1641048_a.	toc	2L	33526	23D1-23D2	Dm.7877	-0,364812
1637971_a.	CG8129	3R	41117	85D12-85D'	Dm.20161	0,348784
1624800_at	CG5224	2R	37128	55D1-55D1	Dm.11215	-0,423798
1627122_at	CG32767	X	31430	4E2-4E2	Dm.6248	-0,378664
1623931_s.	CanA-14F	X	32625	14E3-14F1	Dm.33830	-0,348804
1634287_at	CG13737	3L	39531	70C5-70C5	Dm.14888	-0,315505
1624979_at	CG18594	3R	42644	94A16-94A'	Dm.20618	-0,356697
1625113_s.	CG6509	2L	34573	32F2-32F2	Dm.2056	-0,320079
1623770_at	CG1673	X	32297	11F1-11F3	Dm.10853	-0,339326
1635742_s.	Cad74A	3L	39936	74A5-74A6	Dm.14940	-0,313552
1633682_a.	l(1)G0232	X	31907	8F2-8F6	Dm.20620	-0,345057
1627284_at	PH4alphaPV	3R	43640	100A1-100/	Dm.12823	-0,32277
1639151_a.	CG4272	2L	33419	22E1-22E1	Dm.3019	-0,317096
1634670_at	CG33181	X	318916	7F1-7F1	Dm.20779	-0,296807
1631105_at	Tsp96F	3R	43127	96F5-96F5	Dm.1337	-0,363346
1624717_s.	CG32647	X	326226	11C1-11C2	Dm.13947	0,32431
1632656_at	CG12112	X	31794	7F4-7F4	Dm.2199	-0,304831
1629658_at	CG33298	2L	2768929	30B5-30B1(	Dm.7689	-0,299779
1631037_at	BthD	X	32317	12A8-12A8	Dm.31055	-0,286704

1640289_a.	CG40439	2L	3355120		Dm.28057	-0,302982
1631687_at	RN-tre	2R	36554	50C23-50C2	Dm.614	-0,288308
1632317_at	CG3036	2L	33695	25B1-25B2	Dm.33536	-0,325446
1625794_at	CG5830	3L	39748	72C1-72C2	Dm.15257	-0,360344
1635741_a.	Patsas	2L	34634	33C4-33C4	Dm.23204	-0,261406
1626478_at	CG12340	2R	36133	47C1-47C1	Dm.3126	-0,289528
1628319_s.	sdk	X	31017	1C2-1C3	Dm.4732	-0,29423
1641272_at	CG34352	X	5740340	8D10-8D10	Dm.115	-0,277738
1631499_a.	sws	X	31716	7D1-7D1	Dm.3604	-0,289048
1634991_at	CG8083	2R	35914	45A1-45A1	Dm.4398	-0,320264
1624525_at	CG12998	X	32710	15F4-15F4	Dm.5433	0,290709
1636006_at	CG17766	X	31155	2B15-2B15	Dm.22	-0,274677
1639524_at	CG18604	2R	37154	55E8-55E9	Dm.690	-0,265448
1634520_at	vn	3L	38657	64E12-64F2	Dm.2441	-0,329144
1625058_at	yellow-d	2R	37703	59D9-59D9	Dm.22926	0,253952
1630995_at	chif	2L	34974	35F1-35F12	Dm.3792	-0,276493
1624856_at	c(2)M	2L	34964	35F1-35F1	Dm.19842	-0,254101
1637660_at	CG5150	3L	38633	64D5-64D5	Dm.3933	-0,331698
1631516_s.	skd	3L	43906	78A2-78A2	Dm.3624	-0,287924
1636145_at	CG7219	2L	34091	28D3-28D3	Dm.18230	-0,263666
1638770_a.	cenB1A	3R	42735	94E1-94E1	Dm.6985	-0,241633
1630772_at	sqz	3R	42300	91F4-91F4	Dm.2401	-0,278216
1641412_at	CG31809	2L	318954	36B2-36B2	Dm.30845	-0,275802
1626259_at	su(s)	X	31012	1B13-1B13	Dm.43	-0,267804
1636614_a.	vav	X	32920	18B6-18B7	Dm.218	-0,219486
1623827_a.	fra	2R	36377	49A10-49B3	Dm.2428	-0,241044
1638547_s.	CG7955	3L	38193	62A8-62A9	Dm.12977	-0,212434
1640997_at	CG30122	2R	37146	55E3-55E3	Dm.12100	-0,224354
1627761_at	fat2	3L	40191	76E1-76E1	Dm.3778	-0,239393
1627996_a.	Pi4KIIalpha	3R	40670	83B1-83B2	Dm.7792	-0,218219
1634804_at	CG15819	2L	34028	28A1-28A1	Dm.13216	-0,227358
1623785_a.	pbl	3L	38879	66A18-66A1	Dm.2712	-0,242323
1638378_at	CG13116	2L	34288	30C6-30C6	Dm.4877	-0,231059
1635181_at	CG3337	3R	42519	93C7-93C7	Dm.24083	-0,220433
1628044_at	CG10214	3R	42811	95B1-95B1	Dm.23336	-0,22033
1635310_s.	CG31688	2L	35314	38C6-38C7	Dm.7420	-0,213297
1632819_at	CG31224	3R	42237	91B8-91B8	Dm.12853	-0,194118
1626413_at	Rbp1-like	X	32293	11F1-11F1	Dm.14416	-0,164559
1641078_s.	GalNAc-T1	2R	36717	52A6-52A6	Dm.3404	-0,20906
1626798_a.	Bsg25D	2L	33757	25D2-25D3	Dm.12836	-0,198335
1635117_at	fu	X	32855	17D1-17D1	Dm.17302	-0,191298
1637627_at	Ptp4E	X	31425	4D7-4D7	Dm.1678	-0,212176
1633247_at	CG7006	3R	42963	96B1-96B1	Dm.5514	-0,201249
1636496_at	Nup153	X	32630	14F2-14F2	Dm.7275	-0,203929
1627759_at	CG30080	2R	246441	52C1-52C1	Dm.21323	-0,223334
1628613_a.	CG30494	2R	35698	43E6-43E6	Dm.19215	-0,20571
1639986_at	CG6854	3L	39645	71B4-71B5	Dm.4243	-0,193117
1634904_s.	RhoGEF2	2R	36915	53E4-53F1	Dm.2959	-0,205942
1625345_at	CG18508	X	59253	3C9-3C9	Dm.5987	-0,173204
1634077_at	CG4789	X	32668	15A9-15A9	Dm.5281	-0,207861
1632251_s.	CG6181	2L	34541	32D3-32D3	Dm.12439	-0,192945
1638999_at	rno	3L	38027	61B2-61B2	Dm.15967	-0,177046
1632654_at	l(2)37Cg	2L	35196	37C1-37C1	Dm.30851	-0,203436

1632418_s.	pum	3R	41094	85C4-85D1	Dm.1115	-0,184707
1628825_at	CG8092	2R	36685	51E5-51E7	Dm.3651	-0,196829
1630639_at	dsd	3R	43315	98A10-98A1	Dm.7161	-0,213117
1629552_at	rdgBbeta	2R	37011	54D6-54D6	Dm.671	-0,182238
1623312_s.	CG11836	3R	43007	96B20-96B2	Dm.3637	-0,188614
1634668_at	Ice	3R	43514	99C1-99C1	Dm.2333	-0,161379
1636468_a.	CG5033	2R	37036	54E9-54E9	Dm.676	-0,178561
1641352_at	CG7922	3R	42543	93E2-93E2	Dm.10217	-0,181493
1639575_at	CG18446	2R	36023	46C1-46C1	Dm.5946	-0,157433
1633243_at	sced	2R	35564	42C3-42C3	Dm.11602	-0,173616
1631119_at	Cip4	3L	38534	64B1-64B2	Dm.7348	-0,174514
1631849_at	heix	2L	34961	35F1-35F1	Dm.2593	-0,175672
1638012_at	rt	3L	39297	68C13-68C1	Dm.6018	-0,170655
1636237_s.	l(1)G0222	X	32732	16B5-16B6	Dm.11951	-0,157902
1627612_a.	Crag	X	31800	7F7-7F8	Dm.7132	-0,164058
1638637_at	CG34246	3L	5740624		Dm.35988	-0,18605
1631286_at	CG7857	3L	39671	71D3-71D3	Dm.3221	-0,162004
1632649_at	RluA-2	2L	34440	31F1-31F1	Dm.4124	-0,15797
1631937_at	CG31755	2L	34331	30F5-30F5	Dm.14021	-0,193166
1637022_at	CG3532	3R	41459	87A5-87A5	Dm.18760	-0,172537
1640235_at	W	3L	40009	75C1-75C1	Dm.5624	-0,19865
1625973_a.	CG3038	X	30970	1A1-1A1	Dm.6474	-0,160078
1638854_s.	CG17667	3L	39453	69E2-69E3	Dm.947	-0,18797
1628853_at	CG8915	X	32715	15F4-15F4	Dm.192	-0,175697
1629337_at	CG32721	X	31681	7B7-7B7	Dm.3142	-0,165712
1625740_at	CG7466	2L	34099	28D3-28D1	Dm.2153	-0,236865
1626028_at	CG4783	3R	42381	92B9-92B9	Dm.1257	-0,28689
1635273_s.	kek5	X	32930	18C3-18C7	Dm.33776	-0,296418
1626323_s.	CG17255	X	31976	9C6-9D1	Dm.11437	-0,274898
1626888_at	CG9426	2L	34719	34A10-34A1	Dm.13511	-0,256188
1639731_at	CG7785	3R	42158	90E1-90E1	Dm.6673	-0,314582
1635343_a.	CkIIalpha-i3	3L	38088	61C8-61C8	Dm.2861	-0,274109
1637286_a.	raw	2L	44851	29E4-29E6	Dm.1634	-0,269639
1636409_at	CG11034	2L	33810	25F5-25F5	Dm.26736	-0,278007
1629295_at	CHES-1-like	X	31678	7B6-7B6	Dm.31076	-0,284091
1635000_at	Glycogenin	2R	37419	57D1-57D1	Dm.30881	-0,283871
1630411_at	CG9945	2R	37329	57A7-57A7	Dm.5593	-0,266822
1635938_s.	Ork1	X	32020	9F7-9F8	Dm.3389	-0,280196
1626465_a.	hep	X	32256	11D10-11D1	Dm.5867	-0,299459
1624714_at	woc	3R	47249	97E11-97E1	Dm.1766	-0,278751
1633412_s.	CG3249	X	31459	4F9-4F10	Dm.3811	-0,25255
1626051_at	CG13339	2R	36528	50C6-50C6	Dm.35325	-0,273412
1631691_at	CG16772	2L	35281	38A8-38A8	Dm.5776	-0,311437
1638370_s.	CG12701	X	32994	18F1-18F2	Dm.22844	-0,264295
1641575_at	Cad87A	3R	41441	87A1-87A1	Dm.10799	-0,271548
1633233_a.	CG15817	3R	43462	99A1-99A1	Dm.3012	-0,264853
1623615_at	CG8675	X	32716	15F4-15F4	Dm.8347	-0,240087
1627068_at	Cyp4aa1	2R	36752	52D4-52D4	Dm.33964	0,235215
1627614_at	chn	2R	36678	51E1-51E2	Dm.11696	-0,257258
1631777_a.	fz4	X	31659	6F5-6F5	Dm.92	-0,266314
1633304_at	p24-1	X	32140	10F1-10F1	Dm.5323	-0,294904
1628884_at	PGRP-SA	X	32099	10C6-10C6	Dm.14186	-0,290138
1630430_a.	CG30437	2R	35494	41F1-41F1	Dm.21950	-0,24378

1632499_at	CG14796	X	31111	2B2-2B2	Dm.26431	-0,262563
1635909_at	cic	3R	53560	92E2-92E2	Dm.5727	-0,257907
1629198_at	l(1)G0095	X	31793	7F4-7F4	Dm.4600	-0,236759
1635173_at	pgant5	2L	326151	25C4-25C5	Dm.4033	-0,297323
1628129_a.	Sdic4	X	2768886	19C1-19C1	Dm.34221	-0,282236
1634114_at	CG31517	3R	251711	88A3-88A3	Dm.3608	-0,274874
1625044_at	CG8891	2L	33718	25B5-25B5	Dm.18782	-0,251954
1631875_at	CG1172	3R	40647	83A1-83A1	Dm.5196	-0,238618
1634437_at	CG10973	3L	39447	69E1-69E1	Dm.20075	-0,267711
1639509_at	Nnp-1	2L	44391	34B7-34B7	Dm.7283	-0,263488
1628788_a.	dsh	X	32078	10B4-10B5	Dm.2927	-0,276982
1630944_s.	CG7536	X	32786	16F6-16F6	Dm.29238	-0,256936
1629579_at	CG2478	2L	35325	38D2-38D2	Dm.479	-0,258572
1635103_at	trr	X	31149	2B14-2B14	Dm.21	-0,246059
1625026_at	CG3348	3R	50082	97F1-97F1	Dm.7343	-0,271995
1639771_at	c11.1	X	53436	8D4-8D5	Dm.2077	-0,267387
1627571_at	CrebA	3L	39682	71E1-71E1	Dm.6979	-0,261238
1630018_at	spn-B	3R	41746	88B4-88B4	Dm.4725	-0,249763
1635446_at	CG15043	X	32835	17B5-17B5	Dm.19166	-0,253303
1639534_at	CG6792	2L	34622	33B11-33B1	Dm.4104	-0,283677
1630329_at	CG1716	X	32301	11F4-11F5	Dm.155	-0,244969
1629227_at	baz	X	32703	15F1-15F1	Dm.191	-0,249377
1624857_at	CG17510	2R	49892	41F5-41F5	Dm.20453	-0,259984
1626591_at	siz	3L	40327	78A5-78B1	Dm.1023	-0,254355
1628937_s.	rin	3R	47998	87F6-87F7	Dm.6945	-0,256135
1636974_at	dlp	3L	39596	70E5-70E7	Dm.2840	-0,259681
1632802_at	Jhl-26	2R	36819	53B1-53B1	Dm.19940	0,224506
1633757_s.	CG10188	2L	35237	37E4-37E5	Dm.10953	-0,245836
1632258_s.	Spec2	3R	3772539	83B6-83B6	Dm.4016	-0,241009
1640720_a.	CG14872	3R	41893	88F7-88F7	Dm.16842	-0,226049
1627096_s.	Tkr	2R	38007	60F3-60F5	Dm.6651	0,20461
1628721_at	CG9864	2R	37287	56F11-56F1	Dm.10928	-0,247028
1629852_at	CG9018	3L	38283	62D3-62D3	Dm.12912	-0,245191
1624541_at	RhoGAP19D	X	33048	19D1-19D2	Dm.7998	-0,26198
1637537_at	Lar	2L	35259	37F2-38A2	Dm.24416	-0,266
1628160_a.	dom	2R	45655	57D11-57D'	Dm.7802	-0,236186
1640098_at	BRWD3	3R	42898	95F12-95F1	Dm.5099	-0,239311
1632904_at	CG10354	2L	33964	27C4-27C4	Dm.8576	-0,230105
1633463_s.	CG30463	2R	246627	53D1-53D4	Dm.24533	-0,24849
1633390_at	CG9056	X	32547	13F17-13F1	Dm.13936	-0,232796
1632178_at	enc	3L	44543	63F1-63F3	Dm.1826	-0,263768
1641063_s.	Oatp30B	2L	34268	30B10-30B1	Dm.33540	-0,253602
1629396_a.	CG8486	2L	34112	28F1-28F1	Dm.18615	-0,251027
1637079_at	CG5098	2R	37063	55B4-55B5	Dm.28281	-0,247463
1634558_s.	egh	X	31239	3A6-3A6	Dm.8115	-0,258975
1626218_s.	oaf	2L	33435	22F4-22F4	Dm.7946	-0,234669
1634383_a.	Jhl-21	2L	34624	33B11-33C1	Dm.411	-0,242803
1638856_at	CG2650	X	31252	3B2-3B2	Dm.5779	0,228216
1637017_at	Cont	3R	40553	82A5-82A5	Dm.12849	-0,260842
1640764_at	RpII215	X	32100	10C6-10C7	Dm.2925	-0,25838
1641381_s.	CG13213	2R	36201	47E6-47F1	Dm.4635	-0,242752
1627795_at	Ir	3R	42742	94E4-94E4	Dm.5652	-0,264702
1635367_at	fs(1)h	X	31722	7D3-7D5	Dm.7909	-0,23868

1629145_at	HERC2	X	33035	19C6-19C6	Dm.3302	-0,247809
1627530_at	CG6700	2L	34496	32B1-32B1	Dm.398	-0,250117
1634549_at	CG11750	X	32079	10B5-10B5	Dm.14003	-0,243615
1624021_a.	dlg1	X	32083	10B6-10B1(	Dm.4352	-0,246348
1636092_a.	wapl	X	31187	2D4-2D5	Dm.7140	-0,242554
1638929_at	CG8920	2R	37304	56F16-56F1	Dm.3203	-0,240915
1635182_at	wts	3R	43651	100A5-100/	Dm.4172	-0,252574
1634504_a.	sol	X	44014	19F5-19F5	Dm.2998	-0,266957
1635506_at	CG11777	2R	36108	47A9-47A9	Dm.20580	-0,230306
1624160_at	CG11329	2L	33938	26F6-26F6	Dm.4529	-0,235666
1629819_s.	Sema-2a	2R	36846	53C6-53C7	Dm.14046	-0,247065
1625785_at	l(1)G0196	X	33137	20B3-20B3	Dm.237	-0,23273
1635743_at	Vang	2R	35922	45A4-45A6	Dm.7726	-0,250073
1629078_s.	Pk61C	3L	38017	61B1-61B1	Dm.7033	-0,252174
1632472_s.	CG4699	3R	41911	89A5-89A5	Dm.10603	-0,251534
1637226_at	CG11885	2L	33215	21C2-21C2	Dm.5619	-0,25432
1628759_a.	CG14073	3L	40046	75E1-75E1	Dm.13637	-0,238347
1623565_at	CG17278	3R	50050	93B4-93B4	Dm.3976	-0,25917
1628328_at	GstE10	2R	37105	55C6-55C6	Dm.27766	0,233984
1625532_at	cv	X	44510	5A13-5A13	Dm.6914	-0,226459
1640433_a.	tou	2R	36241	48A2-48A3	Dm.2658	-0,255973
1624353_at	CG32654	X	32209	11B2-11B3	Dm.31053	-0,233236
1627372_a.	lig	2R	35771	44A4-44A4	Dm.517	-0,236911
1641075_at	Nup358	3R	43041	96C8-96C8	Dm.11385	-0,243
1623837_at	Pez	2L	33882	26C3-26C3	Dm.325	-0,251385
1624931_at	cib	X	31359	4A5-4A5	Dm.74	-0,240502
1628199_at	CG15345	X	50402	7E6-7E6	Dm.4909	-0,253742
1632339_s.	cher	3R	42066	89E12-89E1	Dm.6964	-0,232092
1634740_at	tth	X	32318	12A8-12A8	Dm.3521	-0,221829
1636864_at	corto	3R	40616	82E7-82E7	Dm.24160	-0,221922
1631619_a.	MTF-1	3L	39089	67B5-67B6	Dm.4219, Dı	-0,236806
1632784_at	cnk	2R	36952	54B7-54B7	Dm.664	-0,235423
1638135_at	CG10555	X	31771	7E6-7E6	Dm.3671	-0,227733
1631712_at	CG13398	2L	34169	29C3-29C3	Dm.13498	-0,238193
1624208_s.	Arf51F	2R	36699	51F6-51F6	Dm.2891	-0,236544
1625923_s.	Flo-2	X	32425	12F5-13A1	Dm.6791	-0,223251
1634565_at	CG4957	2L	34374	31B1-31B1	Dm.2216	-0,243553
1627054_at	osp	2L	34850	35B3-35B4	Dm.2714	-0,246842
1634255_at	CG12721	X	32176	11A3-11A3	Dm.20582	-0,224933
1641644_s.	crm	X	31268	3B3-3B4	Dm.68	-0,222823
1628109_at	CG1737	X	32077	10B3-10B4	Dm.133	-0,233147
1633026_a.	CG31158	3R	42665	94B5-94B6	Dm.20724	-0,233396
1629430_s.	regucalcin	X	32165	11A1-11A1	Dm.3430, Dı	-0,237092
1631827_at	CG15863	2R	36034	46C7-46C7	Dm.30958	-0,248033
1625503_at	CG32425	3L	40273	77C6-77C6	Dm.1016	-0,269096
1628172_at	msps	3R	41952	89B1-89B2	Dm.4280	-0,231613
1626575_at	CG1657	X	32070	10B3-10B3	Dm.132	-0,235952
1633611_s.	CG32043	3L	39099	67B8-67B9	Dm.6414	-0,23561
1641123_at	CG34186	2R	5740790		Dm.34782	-0,250659
1626499_at	GATAe	3R	41945	89A12-89A'	Dm.1217	-0,251569
1627864_at	l(2)k07433	2R	36978	54C1-54C3	Dm.20641	-0,229809
1639115_s.	rtGEF	2L	35306	38C5-38C5	Dm.477	-0,239891
1633599_a.	Pepck	2R	37131	55D3-55D3	Dm.6919	0,214697

1640042_at	CG12299	2L	34483	32A5-32A5	Dm.396	-0,239813
1623505_s.	Ubi-p5E	X	326237	5E4-5E4	Dm.21220	0,192797
1633436_at	CG14149	3L	39219	67F1-67F1	Dm.6105	-0,239412
1641747_s.	Atf6	2R	35480	41D4-41E1	Dm.21372	-0,229963
1623496_at	kz	X	31205	2F1-2F1	Dm.33	-0,244671
1624778_at	pcm	X	32935	18C7-18C7	Dm.7395	-0,215672
1639917_at	CG32541	X	32889	17F3-18A2	Dm.4349	-0,214572
1637169_at	Nat1	X	32934	18C7-18C7	Dm.14739	-0,245933
1624755_a.	flw	X	44289	9B14-9C1	Dm.7222	-0,244749
1638200_at	pcx	X	31204	2F1-2F1	Dm.12469	-0,221178
1639264_at	Ddx1	3L	40457	79D3-79D3	Dm.2501	-0,23575
1637410_s.	l(2)gl	2L	33156	21A5-21A5	Dm.2784	-0,225681
1631231_at	fz	3L	45307	70D4-70D5	Dm.1456	-0,20827
1631243_s.	l(3)05822	3R	47260	91A1-91A2	Dm.33520	-0,2532
1627171_at	l(3)87Df	3R	49762	87D9-87D9	Dm.20512	-0,249826
1627394_s.	aop	2L	33392	22D1-22D1	Dm.267	-0,211737
1639519_at	CG5850	2L	34327	30F2-30F2	Dm.378	-0,238804
1636161_at	CG3371	3L	38082	61C8-61C8	Dm.11333	-0,22686
1624385_at	CG17735	3R	40596	82D5-82D5	Dm.31226	-0,22312
1641489_at	CG33169	3L	40504	80B1-80B1	Dm.20419	-0,217928
1640380_a.	MED14	3L	38073	61C6-61C6	Dm.9399	-0,237324
1634954_at	CG4756	X	32664	15A8-15A8	Dm.5131	-0,218246
1629779_at	Mcr	2L	44071	28E1-28E3	Dm.1422	-0,245633
1628978_at	CG5360	2R	37723	59E2-59E2	Dm.13577	-0,246729
1641082_at	Corp	X	31764	7E1-7E1	Dm.105	-0,201691
1629367_at	CG15534	3R	43613	99F4-99F4	Dm.27454	0,214552
1632565_at	Bin1	3R	41965	89B7-89B7	Dm.20197	-0,249671
1637467_at	CG5245	3R	41530	87B10-87B1	Dm.27273	-0,238862
1639690_a.	sba	3R	42824	95C9-95C1	Dm.2202	-0,225273
1629117_at	GalNAc-T2	X	32836	17B6-17B6	Dm.7692	-0,218426
1637936_at	CG32512	X	33085	19E7-19E7	Dm.29242	-0,197246
1623543_at	CG5807	3R	42956	96A23-96A	Dm.3209	-0,238103
1623728_at	CG13689	2L	33241	21E2-21E2	Dm.26695	-0,227021
1623776_s.	dsx	3R	40940	84E5-84E6	Dm.3308	-0,267083
1623887_at	CG12367	2R	36301	48E2-48E3	Dm.573	-0,194957
1632840_s.	fus	2R	44095	52B3-52B5	Dm.7727	-0,2221
1639555_at	CG17724	2R	246682	49F4-49F7	Dm.6975	-0,223272
1634533_at	UbcD10	2R	37035	54E9-54E9	Dm.21581	-0,216083
1632326_at	CG30105	2R	246459	54C12-54C1	Dm.30988	-0,212861
1631271_a.	hang	X	32613	14C4-14C4	Dm.7016	-0,237759
1636921_at	l(2)06496	2R	46074	59C2-59C3	Dm.23950	-0,223271
1634015_s.	CG5077	3R	42412	92E2-92E2	Dm.4545	-0,192281
1625684_at	CG11523	3L	40458	79D3-79D3	Dm.23296	-0,167463
1638368_at	CG31674	2L	318878	38F1-38F1	Dm.21186	-0,20215
1638119_at	PGRP-LE	X	32534	13F1-13F1	Dm.3371	-0,19108
1628726_s.	Rgl	3L	44115	70C5-70C5	Dm.6516	-0,194701
1625269_at	CG9004	3L	38303	62D7-62D7	Dm.11397	-0,145842
1637130_at	CG30159	2R	35611	42E4-42E4	Dm.33454	-0,161705
1639349_at	CG8173	X	32753	16C1-16C1	Dm.6499	-0,177907
1638229_at	l(3)01239	3L	46121	68A4-68A4	Dm.11375	-0,196034
1632839_a.	CG8312	3R	41163	85D27-85E1	Dm.11523	-0,160552
1632381_at	dome	X	32976	18D13-18E1	Dm.2836	-0,168058
1631749_at	CG8928	X	32558	14A1-14A1	Dm.10807	-0,176139

1637190_at	CG9149	3L	38147	61F5-61F5	Dm.4949	-0,16298
1637428_a.	pyd	3R	41062	85B2-85B7	Dm.7093	-0,177513
1627668_s.	bbx	X	3772670	19F5-19F6	Dm.2637	-0,177018
1640102_at	CG17622	3R	42674	94B10-94B1	Dm.27376	0,163927
1630565_at	CG17652	2L	33353	22B1-22B1	Dm.18434	-0,161275
1634117_at	CG6204	3R	42877	95F1-95F1	Dm.1312	-0,146447
1636508_at	Nca	3L	40186	76D8-76D8	Dm.6656	-0,18156
1626483_at	Gl	3L	39536	70C5-70C6	Dm.12980	-0,154549
1637560_at	eIF-4G	4	43839	102C5-102(	Dm.7136	-0,162314
1626943_at	CG14609	3R	40864	84D2-84D2	Dm.4996	0,139833
1641733_a.	CG15645	X	32492	13D4-13D4	Dm.17401	-0,136552
1635066_at	CG9063	3L	40456	79D2-79D3	Dm.3029	-0,152365
1629933_at	Sas10	X	31447	4F5-4F5	Dm.19669	-0,139506
1638889_at	CG3918	X	31585	6B1-6B1	Dm.52	-0,142274
1623968_at	CG10631	2L	35262	38A2-38A2	Dm.17940	-0,146248
1624429_at	CG3967	3L	39086	67B5-67B5	Dm.7851	-0,154156
1637329_a.	tho2	2L	326153	22D1-22D1	Dm.21302	-0,150853
1627172_s.	CG10254	3R	42793	95A7-95A7	Dm.3186	-0,142177
1623670_at	CG14562	3L	40439	79A4-79A4	Dm.13295	-0,163511
1640252_at	mRpL9	3R	41859	88F1-88F1	Dm.12652	-0,149504
1631652_at	CG7713	3R	42139	90C5-90C5	Dm.5918	-0,145487
1624617_at	CG1665	2R	36014	46B4-46B4	Dm.12543	-0,130207
1629218_at	Top3beta	X	31565	5E5-5E5	Dm.2946	-0,158575
1630686_at	CG2186	X	32017	9F5-9F5	Dm.11488	-0,216899
1634587_s.	Mnf	3L	39252	68A7-68A7	Dm.7143	-0,220005
1630227_a.	CG8176	3R	261329	85D19-85D'	Dm.26190	-0,219817
1636052_at	l(1)dd4	X	48481	12B4-12B4	Dm.1749	-0,201006
1625333_at	CG33172	X	32526	13E15-13E1	Dm.25390	-0,223072
1629252_at	CG14657	3R	40602	82D6-82D6	Dm.14498	-0,206463
1633335_at	Sos	2L	34790	34D1-34D1	Dm.4109	-0,221092
1639109_a.	CRMP	3R	40675	83B2-83B2	Dm.3083	-0,245233
1640762_at	CG14464	2R	3355132	41E3-41E3	Dm.23697	-0,224434
1640528_at	CG5053	3R	43096	96E8-96E8	Dm.4682	-0,216938
1634576_at	N	X	31293	3C7-3C9	Dm.4702	-0,199271
1639770_at	CG30421	2R	37954	60D16-60E1	Dm.3844	-0,212248
1640122_at	SelG	X	32149	10F4-10F4	Dm.145	-0,246069
1638682_a.	neur	3R	41085	85C2-85C3	Dm.1114	-0,216389
1630795_at	CG12125	X	31775	7E9-7E9	Dm.3784	-0,211744
1635619_a.	cnn	2R	36491	50A8-50A9	Dm.4773	-0,232682
1640837_a.	ImpL1	3L	39499	70A7-70A7	Dm.7761	0,24307
1638845_at	CG18643	3R	41371	86E5-86E5	Dm.31244	-0,221122
1641504_s.	wdp	2R	37548	58D3-58D3	Dm.7396	-0,251898
1640734_a.	CG16718	3R	42340	92A11-92A'	Dm.22485	-0,235473
1638868_at	Mcm10	2L	35390	39B1-39B1	Dm.19862	-0,217985
1635846_a.	orb	3R	42752	94E9-94E9	Dm.1293	-0,233961
1636474_at	CG12602	2L	34587	33A1-33A1	Dm.18113	-0,227386
1624635_at	CG14210	X	32958	18D3-18D3	Dm.6071	-0,237939
1628416_s.	Dp1	2R	37116	55C8-55C8	Dm.7368	-0,232958
1626706_at	katanin-60	3R	53566	82F6-82F6	Dm.1067	-0,193678
1632908_s.	Lerp	3R	43223	97D2-97D3	Dm.16426	-0,207552
1640922_a.	Hnf4	2L	44544	29E4-29E4	Dm.4524	-0,213382
1634858_s.	wkd	3R	41390	86E11-86E1	Dm.16985	-0,204619
1629372_a.	sec71	2L	34785	34D1-34D1	Dm.12060	-0,220094

1629866_at	Suv4-20	X	31015	1B13-1B14	Dm.4968	-0,206186
1623320_at	CG3711	X	31037	1D2-1D2	Dm.44	-0,226495
1638345_at	fog	X	33148	20D2-20E1	Dm.2819	-0,216891
1639438_at	msl-1	2L	35121	36F11-37A1	Dm.458	-0,217348
1639582_at	Pde11	2L	35107	36F6-36F6	Dm.13948	-0,211869
1628127_at	CG9926	3R	41700	88A3-88A3	Dm.1190	-0,21355
1637302_s.	flfl	3R	41675	87F10-87F1	Dm.14510	-0,25138
1637180_at	CG17977	2R	50251	44A4-44A4	Dm.17826	-0,209584
1628901_at	Asx	2R	36612	51A4-51A4	Dm.2970	-0,208112
1625012_s.	vir-1	2L	34652	33E3-33E4	Dm.12811	-0,23169
1633016_a.	stau	2R	37065	55B5-55B7	Dm.4722	-0,234977
1634190_a.	CG6138	2L	34454	32A1-32A1	Dm.20720	0,206968
1641342_at	cag	2R	36157	47C5-47C6	Dm.19906	-0,208625
1632545_s.	CG5522	2R	36881	53D8-53D9	Dm.6392	-0,218029
1638940_at	RhoGAP15B	X	32686	15C4-15C4	Dm.11080	-0,203704
1624589_a.	cenG1A	2L	34803	34D6-34E2	Dm.1977	-0,201577
1629286_s.	Lag1	X	50392	5E3-5E4	Dm.1552	-0,217736
1634211_at	arr	2R	44279	50A9-50A1	Dm.1842	-0,212443
1630165_s.	Sin3A	2R	36382	49B5-49B7	Dm.4374	-0,213271
1626239_at	bon	3R	44235	92F2-92F3	Dm.1848	-0,230601
1631212_at	MED22	X	31038	1D2-1D2	Dm.31032	-0,223224
1637798_at	CG15717	X	32262	11E1-11E1	Dm.5328	-0,230364
1632332_at	CG5447	3R	43192	97B3-97B3	Dm.3550	-0,201984
1631597_at	foxo	3R	41709	88A5-88A8	Dm.3957	-0,222027
1639499_at	CG2025	X	32143	10F2-10F2	Dm.1603	-0,200673
1626494_at	DNApol-eta	3L	40438	79A4-79A4	Dm.3449	-0,185063
1638873_at	CG6175	3L	39271	68C1-68C2	Dm.13613	-0,179614
1638264_at	CG1304	X	33074	19E5-19E5	Dm.12834	-0,265475
1641287_at	CG8301	3R	41160	85D26-85D2	Dm.6393	-0,22405
1634654_at	poe	2L	46243	28D11-28E1	Dm.7936	-0,20819
1639266_at	CG15744	X	2768909	11E9-11E9	Dm.3618	-0,21968
1622931_at	CG5146	3L	38630	64D4-64D5	Dm.852	-0,192053
1626249_s.	CycE	2L	34924	35D4-35D4	Dm.3509	-0,218261
1639803_at	CG5151	3L	39772	72D9-72D9	Dm.3702	-0,211749
1627465_at	Mes4	2R	37560	58D8-58D8	Dm.18851	-0,215127
1637456_at	CG8878	2R	36302	48E2-48E3	Dm.3130	-0,222191
1640871_at	Cortactin	3R	42491	93B8-93B9	Dm.4483	-0,215814
1631419_at	Yp3	X	32339	12C1-12C1	Dm.5739	0,162055
1639309_at	CG12333	3R	42218	91A5-91A6	Dm.12926	-0,22977
1623787_at	CG7144	2L	34064	28C4-28C4	Dm.11204	-0,208214
1632271_a.	CG7971	3L	38206	62A10-62A	Dm.4138	-0,206894
1624520_a.	ftz-f1	3L	40045	75D8-75E1	Dm.995	-0,193621
1625109_at	Rrp1	2L	33500	23C3-23C4	Dm.280	-0,205609
1634178_at	CG4351	X	32259	11D10-11D	Dm.7052	-0,192282
1633749_at	CG6337	2R	36530	50C6-50C6	Dm.5230	0,185544
1626835_at	Syb	2R	36080	46F8-46F8	Dm.19412	-0,217852
1632063_s.	CG11357	3L	38561	64B9-64B9	Dm.20483	-0,217327
1636805_a.	CG8944	X	32517	13E12-13E1	Dm.11763	-0,200582
1637623_at	CG14270	X	31319	3D4-3D4	Dm.4952	-0,236552
1626580_at	aft	2R	37034	54E9-54E9	Dm.19954	-0,166556
1640509_s.	lama	3L	38610	64C12-64C1	Dm.6871	-0,219573
1623281_s.	His2B:CG17	2L	326273	39D3-39D3	Dm.30218	-0,200262
1623830_at	CG13350	2R	36551	50C23-50C2	Dm.611	-0,193825

1635356_a	CG8816	2R	36379	49B5-49B5	Dm.21574	-0,221911
1635437_a	CG11920	3R	43009	96B20-96B2	Dm.1328	-0,214231
1636730_a	CG13373	X	30988	1B4-1B4	Dm.17788	-0,213224
1631772_a.	MTA1-like	3R	40693	83B7-83B7	Dm.7240	-0,204069
1628189_a	Rep2	2R	35955	45C4-45C5	Dm.4000	-0,212877
1628141_a	rhea	3L	38978	66D6-66D7	Dm.894	-0,206557
1633964_a	CG3626	X	31377	4C2-4C2	Dm.13990	-0,214239
1640266_a.	CG7097	2R	37203	56C1-56C4	Dm.7840	-0,220251
1626676_a	plexB	4	43766	102A1-102/	Dm.1700	-0,203344
1624826_a	CG8552	2L	34109	28E7-28E9	Dm.11988	-0,215898
1626444_a	Fib	2R	37662	59C3-59C3	Dm.2821	-0,22296
1638166_a	Rpb10	3R	42942	96A18-96A	Dm.20527	-0,235323
1625276_a.	Eip71CD	3L	39675	71D3-71D4	Dm.2545	-0,244857
1641033_a.	unk	3R	42738	94E1-94E2	Dm.1291	-0,215393
1626232_a	Camta	2R	35974	45E1-45E1	Dm.3685	-0,206795
1633227_s.	CG14945	2L	34623	33B11-33B1	Dm.19822	-0,198479
1629069_a	CG7504	3L	38904	66B7-66B8	Dm.31993	-0,173293
1622925_a	nej	X	43856	8F7-8F9	Dm.4140	-0,19178
1628779_a.	svp	3R	41491	87B4-87B5	Dm.20676	-0,240164
1632964_a	CG15678	2R	37468	57F6-57F6	Dm.18847	-0,217047
1630968_a	CG13907	3L	38104	61D2-61D3	Dm.4970	-0,220285
1631355_a	mRpS28	2R	37136	55E2-55E2	Dm.20392	-0,239456
1629868_s.	CG3838	2L	34275	30B12-30B1	Dm.2108	-0,207681
1632691_a	R	3L	38244	62B7-62B7	Dm.822	-0,226143
1626550_a	CG34449	X	31887		Dm.35876	-0,188723
1628248_a	Scm	3R	41168	85E2-85E2	Dm.6241	-0,215531
1625940_s.	sim	3R	41612	87D11-87D	Dm.4557	-0,207975
1633091_a	CG1789	X	31812	8A3-8A3	Dm.11316	-0,225934
1637757_a	CG10280	3R	40740	83D2-83D2	Dm.27211	0,21418
1639551_a	bip2	4	43793	102B8-102(	Dm.6500	-0,233492
1628451_a	SuUR	3L	45739	68A4-68A4	Dm.7710	-0,190835
1637758_a	CG7737	2R	36174	47E1-47E1	Dm.19908	-0,207743
1639118_a.	Hrb87F	3R	48535	87F7-87F7	Dm.1711	-0,196795
1635792_a.	Ela	3R	42970	96B1-96B1	Dm.5522	-0,210724
1637574_a	Acf1	3R	43751	100D2-100I	Dm.3122	-0,211217
1639574_a.	rdgB	X	32340	12C1-12C4	Dm.7839	-0,201548
1626993_a	Sra-1	3R	41861	88F1-88F1	Dm.3506	-0,205658
1635444_a	CG32663	X	326228	10E6-10F1	Dm.33491	-0,188956
1628548_a	Apc	3R	44642	98E5-98E6	Dm.4863	-0,193045
1630207_a	CG16972	2L	34715	34A9-34A9	Dm.11885	-0,196062
1627434_a	CG5741	3L	39018	66E4-66E4	Dm.14955	-0,215747
1625665_a	mRpL39	3L	39627	71B1-71B1	Dm.2748	-0,204598
1638162_s.	CG1244	3L	38327	62E8-62E8	Dm.11285	-0,189727
1629363_a	Acyp2	3R	41910	89A5-89A5	Dm.11656	-0,234559
1637629_a.	ssh	3R	42986	96B10-96B1	Dm.1518	-0,207978
1632791_a	CG32479	3L	38103	61D2-61D4	Dm.31128	-0,194642
1625833_a	CG9896	2R	37652	59C1-59C1	Dm.13253	-0,199134
1630343_a	CG3679	X	32591	14B9-14B9	Dm.3195	-0,219752
1636673_s.	ttk	3R	48317	100D1-100I	Dm.1526	-0,227575
1634053_a	CG8408	X	32737	16B9-16B9	Dm.194	-0,186633
1639526_a.	Mekk1	3R	42253	91C5-91C5	Dm.4356	-0,208829
1624726_s.	CG7145	3L	40434	78F4-78F4	Dm.11155	-0,217921
1639302_a	CG5645	3L	39382	68F5-68F5	Dm.938	-0,187993

1631112_at	Epac	2R	35588	42C8-42D1	Dm.4217	-0,211968
1637792_at	A3-3	X	43867	1E5-1E5	Dm.10455	-0,215782
1628390_at	CG6151	3L	39720	72A1-72A1	Dm.4008	-0,2183
1628424_at	CG5913	3R	43132	96F6-96F6	Dm.7622	-0,172036
1634524_s.	Hdm	3L	40217	77A3-77A3	Dm.11528	-0,205665
1628425_at	CG31898	2L	319012	29C1-29C1	Dm.3623	-0,164538
1638156_at	fy	2L	34166	29C1-29C1	Dm.4759	-0,163246
1639405_at	CG4857	X	31363	4B1-4B1	Dm.3621	-0,198461
1636302_s.	Su(dx)	2L	33379	22C1-22C1	Dm.2146	-0,226729
1629030_a.	CG18659	2R	35929	45A9-45A1(	Dm.7969	-0,210987
1628984_s.	HmgZ	2R	37480	57F9-57F9	Dm.20604	-0,218551
1639856_at	CG5727	2L	34356	31B1-31B1	Dm.10907	-0,221129
1623287_at	CG13921	3L	38222	62B1-62B1	Dm.14911	-0,216616
1628795_s.	CG9175	2L	33862	26B5-26B5	Dm.3923	-0,186351
1625230_a.	CG31738	2L	34989	36A7-36A9	Dm.11210	-0,192532
1636644_at	RpII18	3R	48312	83A1-83A1	Dm.1757	-0,222554
1630753_at	ric8a	X	31874	8D3-8D3	Dm.6684	-0,205013
1624203_s.	Gli	2L	34927	35D4-35D4	Dm.6515	-0,19358
1632936_at	alpha-Man-IIb	3R	41913	89A5-89A5	Dm.3115	-0,209793
1634304_a.	CG17233	3L	40220	77A3-77A3	Dm.3971	-0,207978
1630911_s.	eIF5	X	32566	14A5-14A5	Dm.173	-0,195278
1637705_at	ewg	X	30975	1A1-1A1	Dm.4971	-0,193548
1631046_s.	CG6448	2L	35442	40E5-40F1	Dm.12142	-0,205516
1635508_at	Taf10	2L	33469	23A5-23A5	Dm.30758	-0,208648
1630396_at	CG4360	3R	42413	92E2-92E3	Dm.10989	-0,204959
1633468_x.	CG41582	Un	5740395		Dm.21410	0,225122
1632630_at	CG7039	X	31841	8C9-8C9	Dm.3677	-0,198866
1633554_at	cos	2R	35653	43B1-43B2	Dm.5775	-0,20424
1633354_at	Cyp6v1	X	33056	19E1-19E1	Dm.12075	-0,184034
1636816_s.	CG33090	2L	34835	35A4-35A4	Dm.7918	-0,208442
1625515_a.	sd	X	32536	13F1-13F4	Dm.6072	-0,1998
1633234_at	CG2807	2L	33235	21E2-21E2	Dm.18458	-0,188308
1637799_at	r-l	3R	42493	93B10-93B1	Dm.1654	-0,217084
1639587_s.	CG7458	3L	40441	79A4-79A4	Dm.11044	-0,165691
1631362_at	CG11279	3L	39486	69F6-69F6	Dm.18896	-0,211697
1638113_at	beta4GalNAc	2R	36585	50E6-50E6	Dm.18830	-0,19192
1624675_at	UBL3	X	32541	13F13-13F1	Dm.7131	-0,211385
1634442_at	smo	2L	33196	21B7-21B7	Dm.244	-0,198995
1635928_a.	Pka-C3	3L	39733	72B1-72B2	Dm.7805	-0,219721
1639951_at	CG2611	2L	35324	38D2-38D2	Dm.19852	-0,206935
1635914_at	CG10413	2L	35111	36F7-36F7	Dm.6777	-0,20877
1627334_at	Tsp39D	2L	35405	39D1-39D1	Dm.22927	-0,194856
1635567_s.	swm	2L	35235	37E4-37E4	Dm.12338	-0,191031
1635153_at	Gfat2	3R	43373	98C4-98C4	Dm.12971	-0,194755
1632134_s.	Axn	3R	43565	99D2-99D3	Dm.7154	-0,203868
1625888_a.	gry	3L	38391	63B11-63B1	Dm.9462	-0,184623
1636868_at	CG14939	2L	34593	33A2-33A2	Dm.2062	-0,18568
1622903_s.	Tango10	X	32125	10D8-10D8	Dm.2186	-0,203507
1640892_a.	Doa	3R	43415	98F1-98F2	Dm.6767	-0,1925
1641481_at	Ugt86Dd	3R	53507	86D4-86D4	Dm.27519	0,16644
1626841_s.	ck	2L	34882	35B8-35B9	Dm.2128	-0,168605
1634527_s.	CG40005	2L	3355118		Dm.33164	-0,196993
1630196_at	Bem46	2L	44441	23C4-23C4	Dm.20302	-0,178372

1625571_at	CG5385	2L	34386	31D4-31D4	Dm.18392	-0,189292
1622981_at	usp	X	31165	2C7-2C7	Dm.25	-0,178729
1628516_at	CG15432	2L	33647	24F2-24F2	Dm.23843	-0,200993
1632089_at	CG4045	X	31176	2C10-2C10	Dm.26191	-0,191087
1640324_at	AnnIX	3R	42492	93B9-93B1(	Dm.4864	-0,215111
1631594_s.	Pi3K92E	3R	42446	92F3-92F3	Dm.1876	-0,199961
1632099_s.	Liprin-alpha	2L	249072	27A1-27A1	Dm.12197	-0,198777
1636287_at	CG10592	3L	38632	64D5-64D5	Dm.5219	-0,209385
1635960_at	slmb	3R	42504	93B13-93C1	Dm.2387	-0,191117
1636919_at	mago	2R	37402	57C2-57C2	Dm.19970	-0,168241
1637163_at	CG7003	3L	39654	71B6-71B6	Dm.966	-0,179681
1635248_at	CG17144	3L	39398	69A2-69A2	Dm.5785	-0,2184
1627589_s.	CG9520	2L	34215	29F5-29F5	Dm.366	-0,189599
1637773_s.	Baldspot	3L	39860	73B4-73B5	Dm.3261	-0,191395
1636033_at	CG31368	3R	41437	87A1-87A1	Dm.10009	-0,180267
1624244_at	S6k	3L	38654	64E8-64E11	Dm.856	-0,206481
1637768_at	CG30100	2R	246457	53B1-53B1	Dm.16014	-0,18814
1640841_at	mts	2L	45959	28D1-28D2	Dm.4245	-0,186526
1635260_at	Stam	2L	34505	32B2-32B3	Dm.401	-0,188125
1628840_at	ken	2R	37785	60A6-60A7	Dm.2792	-0,198479
1639469_a.	Pu	2R	37415	57C7-57C8	Dm.7417	-0,221489
1640240_s.	CG4165	X	31458	4F10-4F10	Dm.7220	-0,17212
1631248_at	l(1)G0020	X	31811	8A2-8A3	Dm.19627	-0,178707
1624058_at	fs(1)N	X	45973	1F3-1F3	Dm.6551	-0,187445
1638133_at	qkr58E-3	2R	37559	58D7-58D8	Dm.2101	-0,194128
1626280_a.	Chrac-14	2L	3772329	29C4-29C5	Dm.3099	-0,199842
1623805_at	CG4875	X	32678	15B4-15B4	Dm.19727	-0,175155
1639764_at	CG30020	2R	36156	47C5-47C5	Dm.23234	-0,16818
1636691_a.	Sfmbt	2L	34709	34A7-34A8	Dm.11543	-0,195414
1627502_at	CG9304	2R	37500	58A4-58A4	Dm.729	-0,194602
1624496_at	xl6	2L	33967	27C4-27C4	Dm.33422	-0,205084
1640730_at	pygo	3R	43718	100C6-100(	Dm.5270	-0,194087
1640130_at	CG31678	2L	35327	38D2-38D4	Dm.19853	-0,183367
1625024_at	zwilch	3R	59247	100B6-100E	Dm.4254	-0,166812
1624644_a.	CG5514	3R	43352	98B6-98B6	Dm.11925	-0,185644
1625218_at	CG11399	3L	40295	77E6-77E6	Dm.4002	-0,183506
1628243_at	dbr	2L	33161	21B1-21B1	Dm.3074	-0,188843
1636371_at	CG2941	X	31348	3F7-3F7	Dm.13423	-0,168682
1640405_at	l(2)01810	2L	47253	33B9-33B9	Dm.1496	-0,202272
1627598_at	CG11490	2L	33184	21B4-21B4	Dm.243	-0,194876
1633778_at	gb	3R	43265	97E2-97E2	Dm.1350	-0,188611
1635771_a.	CG32306	3L	38306	62E1-62E2	Dm.6482	-0,184264
1623874_at	CG14215	X	32971	18D13-18D'	Dm.224	-0,174322
1625437_at	CG32164	3L	59215	73B1-73B1	Dm.1565	-0,191478
1629290_at	Smox	X	31738	7D14-7D15	Dm.2941	-0,18209
1637951_at	CG8461	3R	41758	88C3-88C3	Dm.1196	-0,173997
1627693_at	CG1965	3R	40842	84B2-84B2	Dm.11552	-0,188597
1634660_at	mrt	3R	43291	97F1-97F1	Dm.20267	-0,206258
1634044_at	bib	2L	34330	30F5-30F5	Dm.3236	-0,206559
1640567_at	CG7337	2L	33367	22B4-22B6	Dm.264	-0,191422
1632548_a.	Mes2	3L	40514	80C1-80C1	Dm.4368	-0,169806
1629762_at	CG5148	3R	42065	89E12-89E1	Dm.20203	-0,1713
1623985_at	l(3)j2D3	3L	39374	68F3-68F3	Dm.3655	-0,178877

1634909_at	CG12975	3L	40343	78C2-78C2	Dm.5873	-0,188392
1639444_a.	CG6454	3R	42904	95F14-95F1	Dm.23343	-0,200943
1625295_at	ksr	3R	40660	83A5-83A5	Dm.2488	-0,174722
1635917_at	RpL35	X	31483	5A11-5A11	Dm.19648	-0,194067
1628990_at	Hmgcr	3R	42803	95B1-95B1	Dm.21259	-0,169816
1641304_s.	CG9801	3R	41044	85A9-85A1(	Dm.4969	-0,172865
1634146_at	CG15926	X	32197	11A11-11A'	Dm.12832	-0,17427
1638350_at	CG6712	2L	34631	33C4-33C4	Dm.8705	-0,193404
1631190_at	CG8089	2R	36679	51E2-51E2	Dm.24518	-0,160844
1633403_at	Socs44A	2R	35786	44B3-44B3	Dm.2677	-0,160866
1630281_at	dpr12	2R	50320	42A1-42A1	Dm.17879	0,181931
1625421_at	CG30377	2R	50252	43F1-43F1	Dm.12133	-0,190508
1637223_s.	CG8929	2R	37306	56F16-56F1	Dm.6803	-0,175377
1630764_at	CG8683	2L	326157	28E6-28E7	Dm.21374	-0,204994
1624864_s.	dock	2L	33262	21E2-21E2	Dm.5508	-0,20897
1636122_at	Socs16D	X	32760	16D4-16D4	Dm.6250	-0,178592
1638819_at	CG31712	2L	34320	30F1-30F1	Dm.377	-0,191755
1641445_s.	CG7192	X	32791	16F6-16F6	Dm.17327	-0,190621
1624766_s.	Dsp1	X	117294	14B14-14B1	Dm.2879	-0,175695
1639976_at	CG7222	2R	36138	47C1-47C1	Dm.560	-0,202887
1626980_at	Pli	3R	42821	95C5-95C8	Dm.1303	-0,172472
1641226_a.	mask	3R	50070	95F3-95F5	Dm.7426	-0,184583
1638359_s.	CG14478	2R	36964	54B16-54B1	Dm.11614	-0,194912
1636634_a.	MRP	2L	34686	33F3-33F4	Dm.1929	-0,191171
1627956_x.	CG14216	X	32973	18D13-18D'	Dm.3992	-0,19854
1627431_at	CG8155	2R	36698	51F5-51F6	Dm.4468	-0,177831
1640200_at	Dys	3R	42327	92A5-92A1(	Dm.16710	-0,168988
1636048_at	Dak1	3R	43165	96F10-96F1	Dm.3048, Dı	-0,195524
1623383_at	Dhc64C	3L	38580	64B17-64C1	Dm.846	-0,165525
1639305_a.	Khc-73	2R	36718	52A6-52A1(	Dm.350	-0,182482
1637730_at	CG17361	3L	39548	70C10-70C1	Dm.23537	-0,158342
1628602_a.	CG9248	2L	35384	39A6-39A7	Dm.2220	-0,178729
1626339_at	garz	2R	36337	48F8-48F10	Dm.5126	-0,199657
1631868_at	Vap-33-1	X	31349	3F9-3F9	Dm.4003	-0,190427
1640101_at	CG8152	2R	36696	51F4-51F4	Dm.12835	-0,184242
1635713_at	CG13185	2R	36268	48C5-48C5	Dm.567	-0,17339
1636412_at	MICAL	3R	41225	85F5-85F7	Dm.33543	-0,176151
1635021_at	disp	3R	44274	83C5-83C5	Dm.1845	-0,196026
1632848_at	nmdyn-D6	X	32396	12F1-12F1	Dm.3287	-0,17464
1632922_at	NFAT	X	32321	12A9-12B2	Dm.2918	-0,186727
1638871_at	SCAR	2L	34519	32C1-32C1	Dm.402	-0,195927
1623881_s.	Sirt4	X	31480	5A10-5A10	Dm.11339	-0,17513
1637723_at	mod(mdg4)	3R	49228	93D7-93D9	Dm.35861	-0,190691
1635678_at	CG11299	2R	37755	59F6-59F7	Dm.2037	-0,199419
1629072_at	su(w[a])	X	31054	1E1-1E1	Dm.4721	-0,193875
1636572_at	Duox	2L	33477	23B2-23B2	Dm.11399	-0,183182
1628902_at	mRpS11	3R	42061	89E11-89E1	Dm.20701	-0,195585
1633169_at	dlt	3L	46719	62B4-62B4	Dm.7397	-0,169768
1638970_at	CG14543	3R	43167	96F10-96F1	Dm.5823	-0,206515
1632932_a.	CG33171	3L	38769	65E1-65E2	Dm.11036	-0,183868
1632397_at	CG16868	2R	37298	56F16-56F1	Dm.11943	-0,190387
1634625_at	Dnz1	2L	34503	32B1-32B2	Dm.3214	-0,201668
1630007_s.	Plc21C	2L	33204	21B8-21C1	Dm.4674	-0,195963

1639034_at	CG7593	3R	43500	99B9-99B9	Dm.23486	-0,166924
1640470_s.	Fur2	X	32604	14C1-14C2	Dm.6383	-0,199457
1631645_at	CG3363	2R	37874	60C2-60C2	Dm.4580	-0,190756
1639136_s.	psq	2R	36118	47A13-47B1	Dm.7369	-0,182349
1630640_at	CG4594	2L	34316	30E4-30F1	Dm.375	-0,185089
1630000_at	CG7358	X	32886	17F2-17F2	Dm.11560	-0,196355
1640637_at	MED19	3L	39987	75A4-75A4	Dm.2027	-0,187389
1637962_at	spir	2L	45931	38C5-38C5	Dm.3356	-0,177778
1624037_s.	Taf4	3L	39765	72D7-72D8	Dm.7812	-0,197665
1634001_at	CG9062	2R	36199	47E5-47E6	Dm.14157	-0,188289
1627492_at	CG5018	3L	39774	72D10-72D1	Dm.2039	-0,174654
1623853_at	betaggt-I	2L	33704	25B4-25B4	Dm.12506	-0,174073
1630284_at	CG14082	3L	40088	75F7-75F7	Dm.27176	0,202367
1630636_s.	CG2446	X	32123	10D6-10D7	Dm.19556	-0,195359
1627245_s.	CG5746	3R	42903	95F14-95F1	Dm.1510	-0,184144
1631242_at	jumu	3R	41265	86B1-86B1	Dm.3109	-0,193887
1632882_at	CG11671	3R	40976	84F8-84F8	Dm.31234	-0,191024
1635838_at	CG13545	2R	37675	59D1-59D1	Dm.27784	0,179872
1639459_a.	Nrx-IV	3L	39387	68F6-68F7	Dm.1843	-0,178874
1625343_at	CG5931	3L	39737	72C1-72C1	Dm.973	-0,187437
1637747_s.	CG8841	2R	36336	48F8-48F8	Dm.12120	-0,192297
1640015_at	CG7053	X	32871	17D6-17D6	Dm.33435	-0,201601
1631122_at	CG4656	3R	42734	94D13-94E1	Dm.1290	-0,193611
1639189_at	Lap1	2R	36670	51D4-51D4	Dm.12431	-0,179445
1626985_a.	CG7263	2L	33390	22D1-22D1	Dm.7891	-0,182439
1640066_at	CG1142	3R	40894	84D8-84D8	Dm.5925	-0,193477
1635468_a.	CG7730	3L	39908	73E4-73E4	Dm.3614	-0,165674
1625233_at	Pi3K68D	3L	39329	68D4-68D6	Dm.7619	-0,187769
1640798_a.	inx2	X	31646	6E4-6E4	Dm.3095	-0,170433
1623058_at	CG32676	X	32005	9E1-9E2	Dm.11844	-0,192115
1624690_at	CG6878	3L	39656	71B6-71B6	Dm.29679	-0,193236
1625879_at	Pomp	2L	35342	38E10-38E1	Dm.19856	-0,204866
1629844_s.	rap	X	45922	4C11-4C12	Dm.1470	-0,163634
1640854_at	CG31248	3R	40853	84C4-84C4	Dm.3839	-0,191217
1638979_a.	mu2	3L	38257	62B11-62B1	Dm.6813	-0,178383
1640335_at	CG31304	3R	41817	88E1-88E1	Dm.16861	-0,19236
1633729_a.	Eps-15	2R	37961	60E1-60E1	Dm.781	-0,193069
1629842_at	Gap1	3L	39158	67C10-67C1	Dm.919	-0,200114
1636347_at	Hs6st	3R	42380	92B9-92C1	Dm.33425	-0,176995
1630900_at	Nhe1	2L	33167	21B2-21B2	Dm.33361	-0,182105
1640243_at	CG9267	2L	34762	34C1-34C1	Dm.5879	-0,181475
1624054_at	Adgf-A	3L	39976	75A1-75A1	Dm.1714	-0,208249
1630942_at	CG5742	2R	37072	55B9-55B9	Dm.680	-0,171027
1627927_at	CG8443	2R	36793	52F5-52F7	Dm.7233	-0,191153
1637584_at	pgant3	2R	35627	42E7-42E7	Dm.3144	-0,180658
1630105_at	CG3891	3L	39091	67B6-67B6	Dm.11610	-0,181218
1632618_a.	cno	3R	40620	82F3-82F6	Dm.4251	-0,168974
1628804_at	CG11419	2R	36975	54C1-54C1	Dm.19949	-0,173443
1631520_at	CG1845	2R	35648	43A2-43A2	Dm.11599	-0,204684
1634924_at	Spt6	X	44000	5E4-5E4	Dm.7373	-0,18007
1629577_at	CG5690	3L	38258	62B11-62B1	Dm.4377	-0,172962
1629279_at	CG8211	X	32745	16B10-16B1	Dm.17341	-0,186545
1637703_a.	Socs36E	2L	35085	36E6-36E6	Dm.5707	-0,199965

1640090_a.	slgA	X	33117	19F6-20A1	Dm.6612	-0,197957
1623023_at	Slip1	4	43809	102C2-102C	Dm.11434	-0,194514
1627684_at	lrrk	3R	42447	92F4-92F4	Dm.24081	-0,170325
1628001_at	CG10492	2L	35177	37B12-37B1	Dm.7370	-0,189081
1626112_at	alpha-Man-II	3R	41126	85D15-85D	Dm.2459	-0,193651
1626654_a.	CG11791	3R	43000	96B19-96B1	Dm.1326	-0,194206
1632306_at	CG9467	3R	41207	85E9-85E9	Dm.4583	-0,187314
1639963_at	CG1951	3R	43423	98F5-98F5	Dm.7736	-0,182003
1633560_at	CG16903	X	31178	2C10-2C10	Dm.4166	-0,179441
1626384_at	CG12182	3L	38324	62E7-62E7	Dm.3031	-0,160885
1630099_at	mip120	2R	36499	50A14-50A	Dm.33814	-0,189298
1631087_a.	CG9809	3R	40562	82B1-82B1	Dm.6908	-0,199725
1633419_s.	CG7341	3L	40004	75B10-75B1	Dm.992	-0,184968
1625517_at	Max	3L	40095	76A3-76A3	Dm.3749	-0,192639
1630341_at	mbt	X	32631	14F2-14F2	Dm.4406	-0,161688
1623138_at	pds5	2R	36286	48D7-48D8	Dm.4304	-0,166372
1636741_s.	Hsc70Cb	3L	39557	70C15-70C1	Dm.7118	-0,181222
1634340_at	CG11970	3R	41072	85C1-85C1	Dm.3129	-0,177929
1632355_at	lack	2R	36999	54C12-54D1	Dm.2783	-0,177932
1636771_at	CoRest	X	32941	18C8-18C8	Dm.4085	-0,183318
1639635_s.	CG4196	3R	41827	88E2-88E3	Dm.4290	-0,191617
1635476_at	CG9752	2R	37431	57D8-57D8	Dm.21585	-0,16633
1626520_at	Rad17	3R	41779	88C10-88C1	Dm.1199	-0,176009
1626621_at	CG1407	2R	36043	46D5-46D7	Dm.3930	-0,190126
1625806_at	Pp1alpha-96	3R	42922	96A5-96A5	Dm.2362	-0,195193
1624365_at	CG10915	2R	37076	55B9-55B1C	Dm.12105	-0,175923
1631180_at	CG5728	3R	42899	95F12-95F1	Dm.1316	-0,170357
1629001_at	CG5787	2L	34684	33F3-33F3	Dm.11772	-0,174355
1629098_at	CG7631	2L	34919	35D3-35D3	Dm.26821	-0,184774
1626839_s.	blot	3L	39939	74B1-74B2	Dm.6622	-0,190248
1626355_at	DNApol-epsil	3R	42758	94E13-94E1	Dm.1294	-0,16957
1623257_a.	CG17665	2R	3355010		Dm.10741	-0,188429
1631616_at	CG15308	X	50415	9B4-9B4	Dm.25253	0,193948
1626967_at	CalpB	3L	39165	67D1-67D1	Dm.2874	-0,199161
1635633_s.	bin3	2R	35552	42A13-42A	Dm.2881	-0,173413
1636909_at	CG4078	X	31497	5B6-5B6	Dm.11709	-0,165858
1629148_a.	CG9776	3R	40546	82A3-82A4	Dm.1464	-0,177958
1638765_at	CG7818	2L	34138	29A3-29A3	Dm.351	-0,159318
1626300_at	CG17068	X	33024	19C1-19C1	Dm.23176	-0,166789
1641548_at	CG10289	3L	38714	65A7-65A7	Dm.862	-0,162248
1624237_at	CG15099	2R	37176	55F6-55F7	Dm.28282	-0,177064
1626696_at	E(z)	3L	39203	67E5-67E5	Dm.2823	-0,177958
1641379_a.	CG41099	3R	3355072		Dm.5502	-0,17512
1641317_at	squ	2L	2768920	36A10-36A	Dm.7018	-0,169198
1635290_at	CG11418	X	31081	1F4-1F4	Dm.10897	-0,17521
1623460_at	Aats-leu	3L	38581	64C1-64C1	Dm.1571	-0,167994
1632471_at	CG12155	X	31682	7B7-7B7	Dm.95	-0,17694
1641297_at	hyd	3R	41181	85E5-85E5	Dm.7365	-0,182179
1627443_at	CG5941	X	31536	5D2-5D2	Dm.7753	-0,171794
1637552_s.	shep	3L	38605	64C8-64C11	Dm.20861	-0,163198
1624849_at	Prosap	2R	50225	50D3-50E1	Dm.19025	-0,181678
1628338_at	CG32756	X	318193	5C10-5C10	Dm.13013	-0,161113
1628488_at	CG6051	3R	43271	97E3-97E3	Dm.2180	-0,188271

1627114_at	CG3793	2L	34928	35D4-35D4	Dm.4558	-0,163817
1626313_at	crl	X	44054	14F1-14F1	Dm.1420	-0,179437
1623602_a.	Ptp69D	3L	39443	69D6-69E1	Dm.945	-0,171451
1630123_at	CG3099	X	31906	8F2-8F2	Dm.17569	-0,165925
1627354_at	CG5835	3R	42296	91F4-91F4	Dm.4282	-0,186843
1630324_at	l(2)k07824	2R	36831	53C1-53C1	Dm.11287	-0,171429
1623758_at	CG13123	2L	34303	30D1-30D1	Dm.12887	-0,174541
1629716_a.	CG13284	2L	35021	36B2-36B2	Dm.33838	0,172051
1639947_s.	CG11166	2R	35660	43B2-43B2	Dm.7246	-0,174162
1641403_at	CG14967	3L	38395	63B13-63B1	Dm.20771	-0,166711
1640157_at	CG1678	X	33121	20A1-20A1	Dm.20679	0,143931
1630543_at	CG5161	3L	39769	72D9-72D9	Dm.18901	-0,176772
1626942_at	CG9996	3R	43008	96B20-96B2	Dm.23682	-0,176619
1623039_at	CG3409	2R	35578	42C5-42C6	Dm.2070	-0,192861
1627843_at	CG12253	2L	47730	32C1-32C1	Dm.10633	-0,172509
1640362_at	CG14781	X	31104	2B1-2B1	Dm.4944	-0,15968
1630823_at	CG32210	3L	40127	76B6-76B7	Dm.30375	-0,186555
1629111_at	MED1	3L	40403	78E1-78E1	Dm.6444	-0,180017
1625654_at	GluRIIB	2L	33789	25E6-25E6	Dm.6941	0,170538
1624079_a.	Rab21	X	3355163		Dm.11159	-0,175007
1623782_at	SCAP	2R	35529	42A8-42A8	Dm.13206	-0,169408
1629053_at	CG8370	2R	36761	52D11-52D1	Dm.641	-0,188001
1623473_at	shark	2R	44353	52F8-52F8	Dm.6764	-0,177173
1623777_s.	CG1358	2R	35697	43E3-43E4	Dm.7075	-0,190845
1636273_at	Dpit47	2R	35565	42C3-42C3	Dm.5395	-0,181684
1629973_at	CG6218	3R	41863	88F1-88F1	Dm.20190	-0,170045
1627764_at	CG6194	3R	41868	88F1-88F1	Dm.2122	-0,164524
1633138_at	CG32251	3L	326205	64B1-64B1	Dm.21144	-0,172228
1632433_at	CG17598	X	33140	20C1-20C1	Dm.238	-0,181745
1626117_at	CG5720	3R	42897	95F11-95F1	Dm.3011	-0,179023
1641510_at	Tor	2L	47396	34A4-34A4	Dm.1502	-0,162399
1635530_at	CG1249	3R	40781	83E4-83E4	Dm.9898	-0,193802
1640421_at	CG11811	3L	39213	67E6-67E7	Dm.6149	-0,179165
1639527_at	Klp31E	2L	34422	31E1-31E1	Dm.3297	-0,184952
1628683_at	CG6272	3L	39243	68A6-68A6	Dm.20675	-0,196239
1624113_at	CG7845	2R	35536	42A9-42A9	Dm.4409	-0,174037
1641214_at	CG9298	2L	34197	29E3-29E4	Dm.18211	-0,162576
1625963_at	CG1896	3R	43738	100D2-100D	Dm.1393	-0,193996
1641624_at	CG31957	2L	319046	24C8-24C8	Dm.18349	-0,181256
1634563_at	CG3764	3L	39902	73E2-73E3	Dm.2243	-0,184191
1632998_at	D12	2L	34172	29C4-29C4	Dm.3524	-0,180225
1635638_s.	tomosyn	X	32217	11B6-11B7	Dm.12987	-0,174689
1634555_at	CG11033	3R	41090	85C3-85C4	Dm.18939	-0,174047
1627673_at	sbr	X	43944	9F4-9F5	Dm.1645	-0,168371
1625245_at	CG3759	2L	34258	30B2-30B3	Dm.5988	-0,191558
1630531_at	RhoGAP93B	3R	42496	93B10-93B1	Dm.11758	-0,207586
1636286_at	CG7382	2L	33772	25E5-25E5	Dm.309	-0,171186
1631872_at	CG7755	2R	36816	53A5-53A5	Dm.21579	0,167823
1625855_at	MED30	3L	38078	61C8-61C8	Dm.798	-0,176927
1633515_s.	ago	3L	38516	64A11-64A1	Dm.2559	-0,181504
1629795_at	PTP-ER	2R	37461	57F4-57F4	Dm.3114	-0,181315
1630721_s.	auxillin	3R	40527	82A1-82A1	Dm.9840	-0,173415
1624533_s.	trx	3R	41737	88B1-88B1	Dm.6437	-0,150185

1624103_at	CG7246	X	31833	8C4-8C4	Dm.5351	-0,154861
1641427_at	foi	3L	38976	66D5-66D6	Dm.2818	-0,177259
1639367_a.	CG11508	2R	35787	44B3-44B3	Dm.11519	-0,164325
1634492_s.	CG7597	3L	40385	78D4-78D5	Dm.1943	-0,172207
1639370_at	CG1239	3R	40706	83C1-83C1	Dm.3565	-0,179218
1629943_at	CG7112	3L	38945	66C8-66C8	Dm.13603	-0,182075
1623641_at	CG32856	3R	318251	89A8-89A8	Dm.6089	-0,1905
1624478_at	Roc1a	X	31014	1B13-1B13	Dm.31031	-0,174456
1635214_at	mus308	3R	41571	87C6-87C6	Dm.23677	-0,172727
1632580_at	CG12765	2R	36463	49F11-49F1	Dm.5033	-0,15725
1637611_at	Yippee	X	32295	11F1-11F1	Dm.3461	-0,165499
1633779_s.	loj	3L	38698	65A6-65A6	Dm.1486	-0,183013
1637307_at	CG31694	2L	33489	23B7-23B8	Dm.11419	-0,167188
1636297_at	CG3173	2R	37840	60B5-60B5	Dm.7814	-0,161706
1625632_at	robl	2R	36963	54B16-54B1	Dm.2693	-0,209182
1633730_at	dik	X	32787	16F6-16F6	Dm.2841	-0,166683
1635123_at	glu	2L	35001	36A12-36A	Dm.3063	-0,168619
1635973_at	CG11196	2R	35763	44A2-44A2	Dm.515	0,183779
1628358_at	Tob	X	32574	14A8-14A8	Dm.11284	-0,19144
1641653_at	WASp	3R	43402	98E5-98E5	Dm.6373	-0,190119
1631249_at	CG12713	3L	39724	72A2-72A2	Dm.20089	-0,164573
1625427_at	CG12241	3R	41834	88E3-88E3	Dm.4143	-0,169083
1628155_at	klar	3L	38067	61C1-61C3	Dm.2787	-0,196371
1635649_at	CG8038	3L	38889	66A21-66A2	Dm.31180	-0,17033
1631586_at	CG15250	X	31929	9A3-9A3	Dm.26597	0,161537
1631979_at	MICAL-like	3L	39475	69F5-69F5	Dm.4420	-0,176856
1624750_at	CG8545	2R	36365	49A7-49A7	Dm.2211	-0,161819
1637536_at	CG14303	3R	42236	91B7-91B8	Dm.13713	-0,147672
1633055_at	CG10874	2L	33394	22D1-22D1	Dm.6070	-0,16995
1636517_at	gprs	2R	36862	53C13-53C1	Dm.12423	0,173909
1640110_at	CG11122	X	32046	10A4-10A6	Dm.10786	-0,160925
1637487_at	Dcp-1	2R	37729	59E3-59E3	Dm.4849	-0,17051
1636574_s.	CG33056	3L	326246	78C2-78C2	Dm.1027	-0,189071
1623212_s.	tamo	2R	37864	60B11-60B1	Dm.6387	-0,186748
1630825_at	CG5798	3R	42509	93C1-93C1	Dm.11085	-0,164541
1633144_at	jbug	2R	43997	59A3-59A3	Dm.14398	-0,186538
1627667_at	CG16753	3L	38365	63A5-63A5	Dm.829	-0,186997
1637469_at	pen-2	2R	251430	55C2-55C2	Dm.14392	-0,196758
1628685_at	CG12029	3L	38436	63E5-63E6	Dm.27049	0,179152
1625204_a.	CG8475	2L	34114	28F1-28F1	Dm.4977	-0,167154
1634013_at	mRpL17	3L	38046	61B3-61B3	Dm.14476	-0,185604
1630438_at	CG32409	3L	318016	64F4-64F4	Dm.11429	-0,169502
1635254_at	CG17662	2R	37738	59F4-59F4	Dm.31010	0,180114
1631554_at	CG11473	X	31476	5A8-5A9	Dm.5372	-0,180147
1634453_at	g	X	44819	12B4-12B4	Dm.1817	-0,155685
1627461_at	bsf	2L	35100	36F5-36F5	Dm.453	-0,174047
1639768_at	CG2813	2L	33237	21E2-21E2	Dm.250	-0,182334
1635104_s.	CG33936	3R	41114	85D11-85D	Dm.21258	-0,179869
1641346_at	CG12124	X	31869	8D2-8D2	Dm.17578	-0,16086
1623374_a.	Fim	X	32721	15F8-16A1	Dm.2171	-0,189791
1625144_at	CG6758	2R	37532	58C5-58C5	Dm.11634	-0,162519
1640146_at	CG33116	2L	326259	37F1-37F1	Dm.1955	-0,176308
1624705_a.	fax	3L	39826	72E5-72F1	Dm.6723	-0,172431

1634942_at	CG5114	3L	39624	71A4-71A4	Dm.961	-0,169294
1623043_s.	CG7852	3L	38178	62A3-62A3	Dm.11783	-0,180637
1628824_s.	l(1)G0060	X	31496	5B4-5B6	Dm.4385	-0,179499
1626898_at	CG4751	2L	34551	32D4-32D5	Dm.4426	-0,174751
1624747_at	Ts	2L	33499	23C3-23C3	Dm.30804	-0,136596
1640456_at	CG15439	2L	33651	24F3-24F3	Dm.2115	-0,17832
1640441_at	Mi-2	3L	40170	76D3-76D4	Dm.28317	-0,17704
1641247_at	Ranbp21	X	32970	18D12-18D1	Dm.3479	-0,188779
1626930_at	CG6230	2L	34546	32D4-32D4	Dm.18494	-0,174211
1627326_a.	CG6860	2L	35041	36C7-36C7	Dm.444	-0,175098
1640263_at	CG13993	2L	33857	26B4-26B4	Dm.30771	-0,199512
1640497_at	mib2	2L	35170	37B10-37B1	Dm.11412	-0,173955
1634592_at	ECSIT	3R	40732	83C5-83C5	Dm.3065	-0,158262
1640914_at	CG8838	2L	33551	23E5-23E5	Dm.7989	0,172825
1624257_at	CG18259	X	32866	17D5-17D5	Dm.19742	-0,166327
1635302_at	mus101	X	48309	12B4-12B4	Dm.21710	-0,160142
1632413_at	CG5343	2L	34429	31E1-31E1	Dm.390	-0,175285
1631525_at	l(2)k10201	2R	48373	45D3-45D3	Dm.27473	-0,168994
1627868_at	Prat	3R	40935	84E5-84E5	Dm.9919	-0,168845
1639093_s.	Tom70	2L	34618	33B9-33B10	Dm.11000	-0,182924
1636745_at	Art4	3R	41219	85F4-85F4	Dm.17037	-0,172575
1623549_at	CG1973	3R	43508	99B10-99B1	Dm.6905	-0,169147
1624649_a.	rogdi	3L	39917	73E5-73E5	Dm.4011	-0,178016
1640818_at	pkaap	2L	34957	35F1-35F1	Dm.8778	-0,178895
1636337_s.	pgant6	3L	38346	62F4-62F4	Dm.3519	-0,179395
1634509_s.	fwd	3L	45374	61C1-61C1	Dm.1632	-0,17794
1633060_at	Cyp6g2	2R	36317	48E7-48E7	Dm.18513	0,173988
1629101_a.	CG10395	2R	35490	41E5-41E5	Dm.489	-0,169699
1635186_at	Syx13	3L	39485	69F6-69F6	Dm.1482	-0,17045
1623952_a.	Su(z)12	3L	48071	76D4-76D4	Dm.1941	-0,168734
1638844_s.	CG3714	2L	33626	24D8-24D8	Dm.11340	-0,176897
1630981_at	CG2698	3R	40945	84E8-84E8	Dm.1096	-0,176388
1638653_a.	CG9932	2L	34701	34A4-34A5	Dm.31451	-0,192514
1633731_at	wee	2L	33965	27C4-27C4	Dm.3106	-0,162411
1628969_s.	CG8485	2R	36579	50E4-50E4	Dm.6604	-0,168952
1641396_a.	CG7766	X	31839	8C8-8C9	Dm.4277	-0,163136
1632512_at	CG11820	3R	43004	96B19-96B1	Dm.2031	-0,176581
1625819_at	sno	X	32273	11E3-11E3	Dm.3794	-0,165999
1623090_at	CG30390	2R	37429	57D8-57D8	Dm.15748	-0,176324
1635568_at	CG10080	2R	37462	57F4-57F5	Dm.11761	-0,165416
1630859_s.	Chro	3L	40508	80B1-80B2	Dm.6422	-0,177083
1629133_at	CG3815	X	31571	5E6-5E7	Dm.2312	-0,166774
1638520_at	nudE	3L	39169	67D1-67D1	Dm.6458	-0,178102
1629271_at	CG10444	2R	37294	56F11-56F1	Dm.11252	-0,180716
1633032_s.	CG8177	3L	39147	67C7-67C8	Dm.3977	-0,160546
1622899_at	Rad9	3L	40054	75E2-75E3	Dm.1964	-0,170371
1625244_at	CG15433	2L	33649	24F2-24F2	Dm.12505	-0,15864
1623875_at	CG3434	3L	39098	67B8-67B8	Dm.910	-0,159332
1628427_a.	Rab40	X	32195	11A11-11A1	Dm.148	-0,148238
1632931_at	cta	2L	3355131		Dm.2855	-0,167193
1641438_at	DNApol-delta	3L	39746	72C1-72C1	Dm.2838	-0,151018
1622983_a.	Nup154	2L	34527	32C1-32C1	Dm.404	-0,181099
1627789_at	CG11444	X	31388	4C7-4C7	Dm.3765	-0,187393

1630683_a1	Patr-1	3R	42058	89E10-89E1	Dm.1227	-0,174691
1638257_a1	CG2931	3R	40673	83B2-83B2	Dm.1071	-0,17697
1634824_a.	CG1677	X	31662	7A8-7B1	Dm.33530	-0,180798
1639806_a1	chb	3L	43901	78C1-78C2	Dm.1413	-0,176358
1624139_a1	PhKgamma	X	32120	10D5-10D5	Dm.2615	-0,174896
1631048_a1	CG14434	X	31632	6D7-6D7	Dm.13434	-0,163775
1633915_a1	RnpS1	3R	41147	85D24-85D2	Dm.23312	-0,161062
1623185_s.	jar	3R	42889	95F6-95F8	Dm.7884	-0,186028
1629248_a1	yrt	3R	41656	87E11-87E1	Dm.1183	-0,164831
1638736_a1	CG11458	3L	50110	77F4-77F4	Dm.21436	-0,174153
1626553_a1	CG10376	2L	35126	37A1-37A1	Dm.4386	-0,166538
1641579_a1	mRpS26	3L	40001	75B9-75B9	Dm.6602	-0,177198
1626763_a.	veli	3R	43003	96B19-96B1	Dm.13950	-0,182
1637308_a1	CG11417	X	31083	1F4-1F4	Dm.11	-0,169045
1631013_a1	mbc	3R	42817	95B5-95B7	Dm.3480	-0,160879
1632261_a1	CG32732	X	31638	6E3-6E4	Dm.7994	-0,154725
1637084_a1	CG14883	3R	41997	89B17-89B1	Dm.16812	-0,167572
1626025_a1	CG34267	3L	5740365		Dm.34296	0,173022
1640753_a1	CG41124	3R	3355105		Dm.29383	0,173997
1629246_a1	CG12219	X	31572	5E7-5E7	Dm.6	-0,160595
1631595_a1	CG13393	2L	34159	29B4-29B4	Dm.30817	-0,185517
1626271_a1	CG9634	X	32660	15A3-15A5	Dm.7732	-0,146305
1634193_a1	CG13142	2L	34434	31E2-31E2	Dm.23865	-0,155836
1627651_a.	loco	3R	42672	94B6-94B8	Dm.3231	-0,164819
1641737_a.	YT521-B	3L	38420	63D2-63D2	Dm.7409	-0,158066
1639985_s.	Chc	X	32537	13F5-13F7	Dm.3174	-0,180663
1627403_s.	Paf-AHalpha	X	32529	13E18-13E1	Dm.1672	-0,151415
1625243_a.	E(bx)	3L	44811	61B2-61B3	Dm.1449	-0,157011
1625556_a1	ari-2	2R	37542	58D1-58D2	Dm.4783	-0,165019
1632333_a1	CG6108	2L	34733	34B3-34B4	Dm.23525	0,162843
1633394_a.	CG13900	3L	38093	61D2-61D2	Dm.7679	-0,178032
1641321_a.	CG5284	3L	39759	72D5-72D5	Dm.11624	-0,155928
1641196_a1	Pnn	3R	41185	85E6-85E6	Dm.6024	-0,156026
1633784_a1	CG8379	3R	41060	85B2-85B2	Dm.2106	-0,178412
1639061_a1	enok	2R	37859	60B10-60B1	Dm.766	-0,157814
1636742_a1	nord	2R	37882	60C3-60C4	Dm.5164	-0,167659
1623343_a1	CG4049	2R	37860	60B10-60B1	Dm.15012	-0,160159
1633691_a1	CG32262	3L	38445	63F1-63F1	Dm.2278	-0,148871
1630717_s.	cg	2R	36571	50E1-50E1	Dm.2240	-0,149865
1627470_a1	phl	X	31221	3A1-3A1	Dm.39	-0,179567
1636887_s.	CG11247	3L	40414	78E5-78E5	Dm.1044	-0,173529
1626299_a1	yl	X	32367	12E3-12E3	Dm.2917	-0,161418
1638283_a1	CG9799	3R	41647	87E8-87E8	Dm.1181	-0,152318
1628175_a1	CG6325	3R	41268	86B4-86B4	Dm.3798	-0,159199
1633019_a.	Spf45	2L	3355114		Dm.7003	-0,169291
1641223_a1	CG4933	3L	39811	72E2-72E2	Dm.31123	-0,170399
1636182_a.	CG10948	3L	39459	69E6-69E6	Dm.6029	-0,142598
1637442_a1	ttv	2R	36614	51A6-51B4	Dm.2966	-0,15851
1641329_a1	CG34422	X	32877	17E1-17E1	Dm.214	-0,162627
1627244_a1	CG18870	2R	59218	57F4-57F4	Dm.10711	-0,161871
1641345_a1	CG3808	3L	40074	75F2-75F2	Dm.20111	-0,15322
1631535_a1	Vinc	X	31201	2E2-2E3	Dm.32	-0,163019
1624320_a1	mei-9	X	31373	4B6-4B6	Dm.1684	-0,156178

1628920_at	CG12237	X	32967	18D11-18D1	Dm.3567	-0,164097
1630358_at	CG2162	3L	38360	63A3-63A3	Dm.1490	-0,155284
1625444_at	CG13773	2L	33963	27C4-27C4	Dm.11997	-0,155904
1631308_at	CG10737	2R	37202	56C1-56C1	Dm.14706	-0,196362
1625839_at	X11Lbeta	X	31997	9E1-9E1	Dm.22750	-0,172198
1633415_at	dpr18	X	32572	14A6-14A7	Dm.17381	-0,162705
1633023_at	Acp70A	3L	39494	70A4-70A4	Dm.2898	0,157349
1625481_a.	retn	2R	45976	59F5-59F5	Dm.20640	-0,179996
1639813_at	CG11008	3L	39457	69E4-69E5	Dm.15343	0,170296
1624008_at	eIF2B-epsilon	X	31156	2B16-2B16	Dm.3467	-0,13867
1640589_at	gek	2R	37858	60B9-60B10	Dm.4506	-0,170791
1635377_at	CG13344	2R	36541	50C6-50C6	Dm.610	-0,152638
1630078_a.	CG32000	4	317815	102A6-102A	Dm.20862	-0,175947
1639159_at	CG4069	3L	39437	69C6-69C6	Dm.7287	-0,145148
1624963_at	CG17802	3R	42143	90C5-90C5	Dm.12904	-0,160598
1623388_at	CG15861	2R	37990	60E8-60E8	Dm.7976	-0,166077
1630530_at	CG9296	2L	34196	29E3-29E3	Dm.6187	-0,162598
1638189_s.	S	2L	33281	21E4-21E4	Dm.251	-0,170221
1624029_at	CG3608	2R	37938	60D9-60D9	Dm.10995	-0,161418
1636333_a.	mthl2	3L	38636	64D6-64D6	Dm.12868	-0,166379
1635187_s.	CG1646	3R	43399	98E5-98E5	Dm.31288	-0,148842
1626180_at	Gmer	2R	37638	59B4-59B4	Dm.739	-0,149051
1629023_s.	CG40191	2R	3354930		Dm.7153	-0,154708
1625795_a.	CG3394	2R	37887	60C6-60C6	Dm.7883	-0,126709
1634072_s.	Hmgs	2R	44154	53C1-53C1	Dm.7717	-0,158924
1624848_at	Prp18	3R	42285	91E4-91E4	Dm.23329	-0,150684
1632475_at	msn	3L	44030	62E6-62E7	Dm.1870	-0,150343
1623015_at	CG7671	3R	42209	91A3-91A3	Dm.20212	-0,135417
1639057_at	mRpS31	3L	39749	72C2-72C2	Dm.20090	-0,128037
1629922_at	CG11409	X	31078	1F3-1F3	Dm.2029	0,160117
1627723_at	CG14701	3R	41352	86D8-86D8	Dm.34077	-0,181641
1630537_a.	Dap160	2L	35378	39A4-39A4	Dm.2093	-0,163658
1637327_at	CG11376	2L	33165	21B1-21B2	Dm.12212	-0,16693
1625723_at	CG14710	3R	41393	86E13-86E1	Dm.20173	-0,154687
1623254_s.	CG31531	3R	40583	82D1-82D2	Dm.11428	-0,169668
1640826_at	CG3947	3L	40293	77E6-77E6	Dm.31216	-0,153445
1623324_at	lin	2R	45325	44F6-44F7	Dm.1802	-0,151836
1633338_at	mRpS23	2L	318977	34D6-34D6	Dm.30836	-0,170918
1625030_at	CG6751	2R	36150	47C5-47C5	Dm.30901	-0,147109
1625519_at	CG6453	2L	35056	36C10-36C1	Dm.447	-0,180806
1628216_at	ppan	3R	42502	93B13-93B1	Dm.3008	-0,164546
1626918_at	CG13879	3L	38044	61B3-61B3	Dm.27030	-0,164016
1633387_at	bves	X	33083	19E7-19E7	Dm.7881	-0,155544
1625814_at	CG8230	2R	35897	44F9-44F10	Dm.536	-0,182652
1629020_s.	arm	X	31151	2B14-2B14	Dm.4782	-0,180437
1630110_at	CG12935	2R	36134	47C1-47C1	Dm.6706	-0,155779
1631907_at	CG6038	3L	39321	68D2-68D3	Dm.935	-0,17037
1626208_at	Exp6	X	47141	1B4-1B4	Dm.1770	-0,149651
1625866_at	Lcp65Aa	3L	38709	65A6-65A6	Dm.27065	0,17094
1641673_at	CG8378	2R	36299	48E2-48E2	Dm.572	-0,147078
1627043_at	CG4674	3R	41320	86C7-86C7	Dm.31243	-0,155889
1626968_at	CG7207	3L	38928	66C4-66C5	Dm.3152	-0,160272
1640143_at	CG10463	2L	35264	38A2-38A2	Dm.13522	-0,16032

1628763_at	Ptpmeg	3L	38059	61C1-61C1	Dm.5654	-0,154921
1638175_at	mst	X	33119	20A1-20A1	Dm.2605	-0,166555
1640417_a.	Pkn	2R	35950	45C1-45C1	Dm.7964	-0,160947
1638009_s.	CG9399	3R	41157	85D25-85D2	Dm.5977	-0,172165
1635365_at	CG5198	2L	34432	31E2-31E2	Dm.391	-0,166694
1632466_at	CG9243	2L	35387	39A7-39A7	Dm.30865	-0,171977
1626781_at	Pp2B-14D	X	32624	14E1-14E3	Dm.33340	-0,160763
1630887_a.	CG14998	3L	38491	64A7-64A7	Dm.14813	-0,166766
1623189_at	slik	2R	37893	60C7-60C8	Dm.10858	-0,18361
1627409_at	CG15727	X	32215	11B5-11B5	Dm.5327	-0,157385
1625332_at	CG14764	2R	35749	43F4-43F7	Dm.2038	-0,166447
1625004_at	CG18811	3L	59172	75D5-75D6	Dm.13182	-0,165644
1635526_at	CG15390	2L	33422	22E1-22E1	Dm.4935	-0,13745
1626972_at	CG11874	3R	43436	98F10-98F1	Dm.1365	-0,142534
1632652_s.	CG40100	2R	3355045		Dm.29348	-0,179247
1625387_s.	torp4a	X	31399	4C11-4C11	Dm.19668	-0,191775
1635714_s.	fdl	2R	250735	49A9-49A1(	Dm.3735	-0,177934
1625183_at	smi35A	2L	34831	35A1-35A2	Dm.2597	-0,178582
1634683_at	Mad	2L	33529	23D3-23D3	Dm.7367	-0,165644
1634541_a.	CG3271	2R	35570	42C3-42C3	Dm.495	-0,156412
1634307_at	Graf	X	32522	13E14-13E1	Dm.10748	-0,155032
1623424_a.	babo	2R	35900	44F11-44F1	Dm.5376	-0,152372
1629733_at	Lim1	X	31813	8A5-8B2	Dm.3025	-0,164212
1641309_s.	Iswi	2R	36390	49B10-49B1	Dm.2581	-0,148742
1633850_at	CG18156	3L	38762	65D5-65D5	Dm.6087	-0,160328
1631101_at	CG6480	3L	40228	77B1-77B1	Dm.15115	-0,162299
1635634_at	CG7172	3L	40395	78D6-78D6	Dm.4953	-0,175006
1635347_a.	CG8557	X	32729	16A5-16B1	Dm.1952	-0,179484
1637265_at	CG7550	3L	38901	66B7-66B7	Dm.15495	-0,148322
1641484_a.	otu	X	31789	7F1-7F1	Dm.7029	-0,170229
1631575_at	U2A	2R	35713	43E9-43E9	Dm.14227	-0,149769
1640271_s.	plexA	4	43832	102D1-102I	Dm.1408	-0,16376
1638998_at	cul-5	3R	43434	98F6-98F6	Dm.5893	-0,151515
1623453_at	CG10447	2L	35261	38A2-38A2	Dm.6186	-0,160379
1625732_at	dpr8	X	32387	12E8-12E1C	Dm.164	-0,163166
1628714_at	CG12129	2R	36044	46D7-46D7	Dm.11398	-0,165352
1640230_at	CG34228	2R	5740377		Dm.33405	-0,16152
1628636_a.	CG11943	X	33002	18F4-18F4	Dm.11611	-0,159516
1627576_at	CG8247	2R	35889	44F7-44F7	Dm.5947	-0,15541
1633204_a.	CG10971	3L	39450	69E2-69E2	Dm.11739	-0,156722
1639576_at	Klp68D	3L	39332	68D6-68E1	Dm.2788	-0,144993
1629997_at	CG13858	3R	50058	94A5-94A5	Dm.27952	0,164887
1634822_a.	CG10344	2R	37565	58E1-58E1	Dm.20177, I	-0,150817
1630918_at	a6	X	31127	2B7-2B7	Dm.2135	-0,149747
1632844_s.	CG12004	3L	38185	62A6-62A6	Dm.813	-0,146138
1624364_at	CG4849	3R	43358	98B6-98B6	Dm.28203	-0,146604
1623656_s.	CG1109	3R	40698	83B8-83B8	Dm.4508	-0,14996
1634560_at	140up	3R	41720	88A9-88A9	Dm.10056	-0,171737
1629942_at	CG5292	3R	42079	89F1-89F1	Dm.5476	-0,162229
1627111_at	NHP2	3L	44005	70F6-70F6	Dm.7234	-0,175047
1628292_s.	HLH106	3L	40155	76C5-76C5	Dm.7945	-0,158996
1634849_at	CG9247	2L	35385	39A6-39A7	Dm.30864	-0,159027
1636106_s.	alt	3R	42127	90C1-90C1	Dm.7290	-0,164706

1633190_at	CG31278	3R	41282	86C4-86C4	Dm.17011	-0,150609
1635299_at	Rev1	3L	38079	61C8-61C8	Dm.2203	-0,175631
1635706_at	mRpL20	3L	39477	69F5-69F5	Dm.2752	-0,160265
1627421_at	CG18292	X	32050	10A6-10A7	Dm.2023	-0,156736
1633092_at	cup	2L	33934	26F5-26F6	Dm.333	-0,165401
1635819_at	sty	3L	38424	63D2-63D3	Dm.2561	-0,172767
1634526_a.	qua	2L	35058	36C10-36C1	Dm.2984	-0,157741
1634293_at	Alg-2	3R	3355106		Dm.5985	-0,142879
1631832_at	Mlc-c	X	31474	5A8-5A8	Dm.3980	-0,160847
1638882_at	ry	3R	41605	87D9-87D9	Dm.2436	-0,188347
1624138_at	CG5800	X	32742	16B10-16B1	Dm.13464	-0,167152
1629638_at	Als	2R	36588	50E6-50E6	Dm.23922	0,165845
1630696_at	CG12972	3L	40363	78C8-78C8	Dm.24788	-0,149897
1637720_at	CG6856	3L	40052	75E2-75E2	Dm.6165	-0,147361
1635035_at	CG9384	3L	39608	70F4-70F4	Dm.31156	-0,139945
1632567_at	CG31342	3R	41592	87D7-87D7	Dm.11941	-0,154098
1626175_at	CG11534	3L	39381	68F5-68F5	Dm.4305	-0,158319
1639759_s.	vlc	2R	46078	41F8-41F8	Dm.1631	-0,151074
1638318_at	CG7544	2R	36680	51E2-51E2	Dm.634	-0,141444
1638511_at	Aats-trp	3R	45399	85D7-85D7	Dm.3001	-0,166404
1623722_at	nonA-l	3R	42026	89D6-89D6	Dm.1225	-0,15215
1623889_at	CG1317	3L	38300	62D7-62D7	Dm.4190	-0,150041
1626774_s.	robo	2R	37603	59A2-59A3	Dm.7485	-0,152734
1635027_at	CG31800	2L	318947	37B10-37B1	Dm.17964	-0,161514
1626833_at	CG11839	3R	43006	96B20-96B2	Dm.16509	-0,162704
1636928_s.	CG1486	X	33108	19F4-19F4	Dm.2324	-0,175713
1634037_s.	Ssdp	3R	42177	90F6-90F7	Dm.13397	-0,154963
1627267_at	MESR3	2L	35116	36F7-36F9	Dm.2155	-0,177279
1630216_at	drosha	2R	35747	43F2-43F3	Dm.4482	-0,172951
1636935_at	CG2219	4	43771	102B1-102E	Dm.13309	-0,159154
1637612_at	CG6017	3L	39747	72C1-72C1	Dm.11460	-0,157195
1633746_s.	a	2R	43852	58C1-58C5	Dm.33421	-0,151067
1628167_s.	CG6597	3L	40231	77B2-77B2	Dm.11689	-0,157831
1636870_at	CG9793	3R	41041	85A9-85A9	Dm.31299	-0,155361
1635376_at	CG9086	X	32687	15C5-15C6	Dm.7014	-0,159554
1628306_at	trc	3L	40165	76D1-76D1	Dm.2518	-0,151317
1639612_s.	CG4323	3R	42423	92E12-92E1	Dm.11879	0,149433
1635051_a.	Herp	2L	34065	28C4-28C4	Dm.6736	-0,163644
1623733_at	Nmd3	X	31195	2E1-2E1	Dm.7201	-0,14886
1623365_at	Bgb	3L	38198	62A9-62A9	Dm.4101	-0,162668
1630101_s.	Smr	X	32225	11B10-11B1	Dm.7194	-0,147159
1632003_a.	Ptp99A	3R	43469	99A11-99B1	Dm.12683	-0,16954
1634411_at	CG6506	X	32766	16E1-16E1	Dm.11571	-0,153545
1628198_at	okr	2L	33507	23C4-23C4	Dm.19774	-0,146502
1630824_a.	CG33145	2R	36279	48D5-48D5	Dm.6148	-0,159102
1640198_at	CG5664	3L	40371	78D2-78D2	Dm.1037	-0,154113
1631381_at	Uba2	3L	44496	66B7-66B7	Dm.6538	-0,163422
1636706_at	Myd88	2R	35956	45C5-45C5	Dm.2113	-0,152837
1630622_at	Sirt6	3R	41254	86A3-86A3	Dm.24872	-0,141456
1625142_s.	CG7377	3L	2768978	68C13-68C1	Dm.29146	0,168742
1639633_at	CG8272	2R	35875	44F2-44F3	Dm.534	-0,149081
1640673_at	Noa36	3R	43384	98D6-98D6	Dm.7297	-0,150907
1633054_at	CG14057	3L	39915	73E5-73E5	Dm.24015	-0,160281

1630140_at	CG14931	2L	34588	33A1-33A1	Dm.26793	-0,160489
1625537_at	CG3655	X	31056	1E1-1E1	Dm.1956	-0,151937
1638704_at	Kap3	X	32071	10B3-10B3	Dm.14732	-0,150586
1636831_s.	brm	3L	39744	72C1-72C1	Dm.5356	-0,153349
1641031_s.	jigr1	3R	43093	96E6-96E6	Dm.7493	-0,157848
1640376_a.	CG30054	2R	246420	49B9-49B9	Dm.33112	-0,13508
1638609_s.	MBD-R2	3R	41498	87B7-87B8	Dm.13212	-0,155307
1636514_at	Tak1	X	39659	19D2-19D2	Dm.6425	-0,165004
1636958_s.	l(2)k01209	2R	36953	54B7-54B7	Dm.10860	-0,140614
1640054_at	D19A	3L	38718	65A7-65A7	Dm.2988	-0,147691
1623826_at	CHIP	2L	34433	31E2-31E2	Dm.392	-0,157318
1640231_a.	CG8908	2R	37293	56F11-56F1	Dm.26975	0,148226
1627383_at	asp	3R	42946	96A19-96A2	Dm.7038	-0,145142
1635342_at	CG10306	2R	37475	57F8-57F8	Dm.3730	-0,164268
1640418_at	mor	3R	41942	89A8-89A1	Dm.2417	-0,160505
1639984_at	raps	3R	53569	98A8-98A8	Dm.7372	-0,166815
1631106_s.	Aats-ile	3L	45785	79D4-79D4	Dm.6892	-0,163821
1627677_s.	CG10743	3L	39524	70B4-70B4	Dm.15315	-0,147856
1630290_at	mld	3R	2768685	96A9-96A9	Dm.31276	-0,150219
1634162_at	CG40198	3R	3354922		Dm.24163	0,138206
1632511_at	CG12862	2R	36625	51B7-51B7	Dm.16062	0,171132
1640619_at	CG7028	3L	38032	61B2-61B2	Dm.3161	-0,152668
1625011_at	CG11206	2R	37552	58D3-58D4	Dm.12465	-0,171051
1628774_a.	boi	X	31229	3A3-3A3	Dm.40	-0,168064
1625344_at	fu12	2L	34176	29C5-29D1	Dm.356	-0,172424
1628264_a.	RecQ5	3L	39594	70E5-70E5	Dm.4338	-0,144871
1634867_at	CG13169	2R	36326	48F1-48F1	Dm.23913	0,151182
1629104_at	CG2258	X	31729	7D6-7D6	Dm.11529	-0,150542
1627239_s.	CG17034	2R	36488	50A5-50A8	Dm.7647	-0,139724
1634817_at	or	2R	43943	60A14-60A	Dm.3362	-0,162729
1630055_a.	sec31	2R	35877	44F3-44F3	Dm.30896	-0,166713
1625945_a.	CG1233	3L	38063	61C1-61C1	Dm.23271	-0,146659
1632525_at	CG9925	3R	41702	88A3-88A3	Dm.1191	-0,160894
1635314_at	maf-S	2R	37336	57A8-57A8	Dm.6801	-0,170146
1625679_at	CG15386	2L	50188	22D4-22D4	Dm.18411	-0,17899
1628755_at	Gfr	3R	40981	84F10-84F1	Dm.31298	-0,160697
1633951_at	DDB1	3R	41611	87D11-87D	Dm.3215	-0,159616
1634649_at	mys	X	44885	7D5-7D5	Dm.1814	-0,174527
1641440_a.	CG4877	3L	39821	72E4-72E4	Dm.7165	-0,168973
1624053_at	CG33967	3R	41783	88D1-88D1	Dm.16870	0,145231
1628025_at	CG13055	3L	39779	72D10-72D	Dm.27863	0,149317
1638737_at	CG17209	X	32524	13E14-13E1	Dm.14839	-0,145282
1641072_at	PEK	3R	40653	83A4-83A4	Dm.3303	-0,165158
1625488_s.	Rho1	2R	36775	52E5-52E5	Dm.644	-0,16347
1623934_at	blp	3R	41937	89A8-89A8	Dm.14264	-0,18034
1629525_at	CG30152	2R	246485	57A10-57A	Dm.11199	-0,170718
1625876_at	AP-1sigma	3R	42835	95D1-95D1	Dm.1305	-0,157215
1625875_at	CG10635	3L	38615	64C13-64C1	Dm.19075	-0,160952
1634329_at	CG32579	X	318096	14B12-14B1	Dm.10769	-0,141247
1632753_a.	CG3980	2L	33610	24C8-24C8	Dm.7915	-0,151561
1627806_at	CG11133	3L	40512	80B2-80B2	Dm.15043	-0,159132
1626266_at	MAGE	3R	40860	84C7-84C7	Dm.4924	-0,152159
1634217_s.	Crk	4	43775	102B1-102E	Dm.3226	-0,157935

1639838_a1	CG8483	3R	41623	87E2-87E2	Dm.4446	0,167155
1624141_a1	CG4901	2L	34357	31B1-31B1	Dm.10854	-0,164473
1624310_s.	CG4753	3L	39819	72E4-72E4	Dm.6346	-0,164782
1625688_a1	CG6293	3R	41259	86A7-86A7	Dm.2071	-0,148479
1624812_a1	CG11070	2L	33932	26F3-26F4	Dm.331	-0,149989
1635580_a1	Cbl	3L	38961	66C12-66C1	Dm.2332	-0,166531
1623479_a1	Rbf	X	31027	1C5-1C5	Dm.4654	-0,151617
1639288_a1	Mys45A	2R	35925	45A7-45A8	Dm.3517	-0,147705
1625505_a1	CG13896	3L	38089	61C8-61C8	Dm.25434	-0,144318
1628469_a.	CG32529	X	33000	18F4-19A2	Dm.31021	-0,160112
1634405_s.	Pak	3R	44039	83E4-83E4	Dm.1868	-0,144177
1628420_s.	CG6923	3R	41420	86E17-86E1	Dm.7827	-0,14951
1634150_s.	Hr78	3L	40378	78D4-78D4	Dm.6935	-0,154502
1640285_a1	Tm1	3R	41852	88E12-88E1	Dm.19116	0,17039
1628080_a1	GIIIspla2	X	31747	7D16-7D17	Dm.6657	-0,154798
1636279_a1	l(1)1Bi	X	31010	1B12-1B12	Dm.5268	-0,144177
1623989_a1	CG5734	2L	34354	31B1-31B1	Dm.3692	-0,167436
1638678_a1	CG6522	2R	36965	54B16-54B1	Dm.666	-0,16357
1637124_a1	sec13	3R	44437	94E13-94E1	Dm.7682	-0,164639
1635824_a1	Tip60	X	31362	4A6-4B1	Dm.75	-0,146368
1623040_a1	CG4449	3R	42748	94E8-94E9	Dm.13736	-0,149245
1626337_a1	Eap	2R	37964	60E1-60E1	Dm.30940	-0,159407
1637618_a1	Hs2st	2L	44433	37E1-37E1	Dm.4274	-0,156615
1626585_s.	CG4589	2R	37912	60D3-60D3	Dm.7406	-0,157206
1631019_a1	CG5971	3L	38989	66D9-66D9	Dm.4495	-0,153903
1627459_a1	CG9154	2L	33858	26B4-26B4	Dm.322	-0,146378
1624997_s.	05-sep	2R	43990	43F8-43F8	Dm.3096	-0,147745
1624620_a1	Klp61F	3L	38135	61F4-61F4	Dm.804	-0,139547
1639200_a1	CG14333	3R	42088	90A3-90A3	Dm.27934	0,149755
1637053_a1	Cas	2L	35016	36B1-36B2	Dm.442	-0,145673
1639826_a1	CG32662	X	32136	10E6-10F1	Dm.4433	-0,166126
1636108_a1	CG17033	3L	39735	72B2-72C1	Dm.972	-0,147814
1624390_a.	thoc7	3L	38033	61B2-61B2	Dm.15964	-0,161336
1633593_s.	CG33223	X	2768867	7F1-7F1	Dm.33755	-0,146082
1640644_a1	Surf6	3R	42370	92B6-92B6	Dm.31323	-0,152265
1633822_a1	CG7519	3L	40377	78D4-78D4	Dm.5966	-0,153787
1630858_s.	CG7816	3R	43533	99C4-99C5	Dm.7928	-0,13793
1623012_a1	mRpL55	3R	42290	91F1-91F1	Dm.3421	-0,160994
1633752_a1	CG34413	2R	37979	60E5-60E5	Dm.7939	-0,162162
1638203_a1	CG7339	3L	39310	68D1-68D1	Dm.11797	-0,14943
1625908_a.	TppII	2R	36444	49F4-49F4	Dm.7374	-0,161269
1627027_a1	CG15168	2L	35159	37B8-37B8	Dm.6168	-0,164048
1637861_a1	CG7786	2R	36802	53A1-53A1	Dm.27756	0,13228
1634866_a1	CG4880	X	32680	15B4-15B4	Dm.14809	-0,135353
1631931_s.	Sdc	2R	37447	57E1-57E6	Dm.721	-0,151266
1638846_a1	CG3402	3L	38077	61C8-61C8	Dm.2160	-0,142951
1626710_a1	SA	2L	33974	27C7-27C7	Dm.336	-0,144818
1635980_s.	CG12991	X	32734	16B6-16B7	Dm.13387	-0,174438
1641424_a1	CG9723	X	32639	14F5-14F5	Dm.2036	-0,15104
1636773_a.	CG10981	3R	40725	83C4-83C4	Dm.1077	-0,148831
1632256_a1	Taf1	3R	40813	84A1-84A1	Dm.7380	-0,155901
1634722_s.	mbf1	3L	39842	73A9-73A9	Dm.7996	-0,162784
1629737_a1	dpa	2R	35679	43C6-43C7	Dm.21562	-0,136724

1639617_at	Gr39a	2L	117346	39C1-39C1	Dm.27684	-0,135285
1638651_a.	CG3508	3R	41778	88C10-88C1	Dm.4480	-0,141824
1626219_s.	Ogt	2R	35486	41E4-41E5	Dm.2728	-0,158337
1640746_at	CG18542	3R	41176	85E4-85E4	Dm.1130, Dı	-0,160014
1634714_at	cactin	X	33043	19D1-19D1	Dm.2875	-0,128626
1641461_at	CG34408	X	31953	9B4-9B4	Dm.3020	-0,14833
1640161_at	e(y)2	X	45848	10C7-10C7	Dm.31083	-0,166154
1626506_at	Syx18	3R	42933	96A12-96A	Dm.1652	-0,142017
1623176_at	CG16812	2L	34722	34A10-34A	Dm.4237	-0,144477
1623902_at	tsu	2R	35924	45A7-45A7	Dm.19884	-0,152875
1629307_s.	hdc	3R	43604	99E4-99F1	Dm.1909	-0,133014
1628429_at	Sec61gamm	X	32968	18D11-18D	Dm.6361	-0,170467
1625500_at	Mlh1	2R	35796	44B8-44B8	Dm.26257	-0,1507
1632301_at	CG9536	2L	33904	26D7-26D7	Dm.6442	-0,145826
1638658_at	Gos28	3R	248102	91B8-91B8	Dm.12874	-0,14607
1632815_at	CG16952	X	32546	13F17-13F1	Dm.7172	-0,150449
1625201_s.	CG17230	3R	41376	86E5-86E6	Dm.4548	-0,132878
1627152_at	CG12918	2R	36046	46D9-46D9	Dm.12129	-0,164727
1631519_at	CG9143	2R	37296	56F14-56F1	Dm.22095	-0,151421
1623698_s.	lap2	2R	36748	52D2-52D2	Dm.7173	-0,158932
1623692_s.	rho-4	X	32109	10C9-10C1(	Dm.1673	-0,14959
1635013_s.	CG10722	2L	35298	38B6-38B6	Dm.14744	-0,134
1637004_at	pcs	2R	36674	51D6-51D7	Dm.4202	-0,149087
1633727_s.	wnd	3L	40143	76B9-76B9	Dm.12913	-0,168401
1636291_at	Rae1	2R	37467	57F6-57F6	Dm.11463	-0,151495
1629185_at	CG6734	2L	34616	33B8-33B9	Dm.18103	-0,147708
1639178_s.	mael	3L	40489	80A1-80A1	Dm.5827	-0,149244
1627873_at	CG5641	3R	41529	87B10-87B1	Dm.1170	-0,15493
1629270_at	Sry-delta	3R	43572	99D3-99D3	Dm.1914	-0,132854
1627207_at	CG14352	2L	33341	22A3-22A3	Dm.5941	-0,137755
1641091_at	CG3280	3L	39117	67C2-67C2	Dm.15446	0,151907
1637056_s.	CG11200	2R	37301	56F16-56F1	Dm.706	-0,165288
1633367_at	CG6340	X	32482	13C5-13C5	Dm.169	-0,141265
1629692_at	CG15653	2R	37388	57B16-57B1	Dm.28040	-0,15769
1632043_a.	shtd	X	32473	13C3-13C4	Dm.4453	-0,129677
1638147_at	CG7772	X	32776	16F1-16F1	Dm.4618	-0,146072
1623819_at	RanGap	2L	35223	37E1-37E1	Dm.3220	-0,136914
1634222_at	CG18596	3R	42622	94A6-94A6	Dm.1987	-0,159577
1638386_at	HLH3B	X	31249	3B1-3B1	Dm.13010	0,147613
1635647_at	CG2617	2L	35331	38D5-38D5	Dm.19855	-0,143456
1633349_at	TfIIFbeta	3R	41290	86C6-86C6	Dm.20167	-0,147931
1634386_s.	Rab4	2R	36992	54C9-54C1(	Dm.2700	-0,148685
1636085_at	CG18143	3R	40528	82A1-82A1	Dm.23299	-0,150842
1639552_s.	CG6951	3L	40222	77A3-77A4	Dm.33820	-0,143186
1625639_at	CG6737	2L	34482	32A5-32A5	Dm.27677, I	0,144513
1632687_at	mib1	3L	39750	72C2-72C2	Dm.10758	-0,144064
1628148_at	unc-119	X	31664	7B1-7B1	Dm.93	-0,130293
1626680_at	CG9305	2L	34748	34B8-34B8	Dm.11440	-0,143989
1639632_at	CG1234	3R	40870	84D3-84D3	Dm.4465	-0,149753
1633167_s.	CG32496	X	318054	16F1-16F1	Dm.31025	0,144229
1623087_at	Gef64C	3L	38578	64B13-64B1	Dm.3853	-0,164345
1627320_at	zfh2	4	43795	102C1-102(	Dm.3906	-0,159108
1629033_at	CG2277	3L	38148	61F5-61F5	Dm.4498	-0,136038

1634116_at	CG7158	3L	40410	78E3-78E3	Dm.3610	-0,153481
1635407_at	CG32772	X	31420	4D5-4D5	Dm.1986	-0,125874
1637810_at	CG5780	2L	34676	33F3-33F3	Dm.5424	-0,145457
1639376_at	CG10591	3L	38634	64D5-64D5	Dm.10982	0,139718
1635588_at	CG14977	3L	38454	63F5-63F5	Dm.14162	-0,164395
1637118_at	CG30010	2R	246389	46B4-46B4	Dm.14599	-0,157221
1634724_at	CG12499	3R	42637	94A14-94A1	Dm.24981	-0,127008
1637517_s.	KrT95D	3R	42843	95D5-95D8	Dm.6042	-0,142824
1636718_s.	CG4502	2L	34002	27E4-27E4	Dm.340	-0,157235
1626738_at	CG4644	2L	33285	21F1-21F1	Dm.12209	-0,133007
1624152_at	CG11851	3R	43031	96C6-96C7	Dm.7685	-0,149367
1630703_at	CG8963	2R	36913	53E4-53E4	Dm.4633	-0,153399
1640430_s.	CG15881	3L	40159	76C5-76C6	Dm.6970	-0,16087
1623810_at	CG17265	2L	33509	23C4-23C4	Dm.283	-0,15481
1629592_at	CG8931	X	32564	14A5-14A5	Dm.4581	-0,138412
1623031_a.	RecQ4	3L	53438	66B10-66B1	Dm.1556	-0,140525
1629305_at	Nup214	2R	46091	59B4-59B6	Dm.12859	-0,140403
1633911_a.	CG31550	3R	40661	83A5-83A5	Dm.3932	-0,140398
1625211_s.	CG32810	X	31110	2B1-2B1	Dm.15	-0,141922
1630514_at	Gprk1	2R	3355013		Dm.1704	-0,159133
1623194_at	CG7637	2R	36135	47C1-47C1	Dm.30961	-0,171573
1638600_at	ebi	2L	33212	21C2-21C2	Dm.3639	-0,165563
1641279_at	ben	X	32358	12D2-12D2	Dm.2088	-0,166134
1638782_at	CG8195	2R	36725	52A12-52A1	Dm.4193	-0,147268
1632728_at	Gcn2	3R	43709	100C3-100C	Dm.2985	-0,134785
1625046_at	CG10648	X	31818	8B7-8B7	Dm.2116	-0,136745
1631768_at	qkr58E-1	2R	37561	58D8-58D8	Dm.2571	-0,142187
1638758_at	CG3016	X	31519	5C5-5C6	Dm.2026	-0,142245
1639206_at	CG15914	X	32542	13F14-13F1	Dm.31058	-0,15205
1632161_at	nbs	3L	44259	67C5-67C5	Dm.1431	-0,155001
1630151_at	spn-D	3R	318579	97E6-97E6	Dm.6085	-0,14379
1624665_at	CG9257	2L	35375	39A2-39A2	Dm.6325	-0,14673
1635581_at	CG8300	X	31658	6F5-6F5	Dm.91	-0,134428
1626584_a.	SMSr	3L	38823	65F7-65F9	Dm.874	-0,144407
1631876_at	CG15266	2L	34893	35C5-35C5	Dm.26815	-0,169116
1641378_at	phtf	2R	35583	42C7-42C7	Dm.3021	-0,148506
1637398_a.	capu	2L	33611	24C8-24C9	Dm.4776	-0,137875
1634171_at	CG12231	X	32942	18C8-18C8	Dm.27591	0,152895
1627871_at	Abd-B	3R	47763	89E4-89E5	Dm.1763	0,145805
1631194_at	CG10265	2R	36676	51D7-51D7	Dm.3825	-0,134808
1625844_s.	apt	2R	37734	59F1-59F4	Dm.6653	-0,122124
1637214_a.	scra	2R	35696	43E3-43E3	Dm.508	-0,143543
1623793_at	krz	3R	53554	100E3-100E	Dm.5655	-0,133075
1627331_at	Taf5	2R	47900	47C5-47C5	Dm.19905	-0,137424
1625464_at	Marf	X	31581	5F4-5F4	Dm.11038	-0,171201
1624894_s.	CSN8	2L	49077	29B2-29B2	Dm.33149	-0,15047
1638125_a.	msi	3R	43087	96E2-96E4	Dm.1334	-0,151692
1638673_at	Bap	X	32987	18E4-18E5	Dm.2885	-0,15794
1629493_at	U26	2L	34175	29C5-29C5	Dm.3089	-0,128809
1623487_at	dmt	3R	41180	85E5-85E5	Dm.2455	-0,148162
1638303_at	CG33160	3L	326265	63B12-63B1	Dm.31165	0,15393
1626481_a.	tafazzin	2R	36405	49C2-49C2	Dm.6762	-0,153632
1641711_at	CG13360	X	31025	1C4-1C4	Dm.19662	-0,136765

1626523_a.	Aats-glupro	3R	42834	95D1-95D1	Dm.1304	-0,14877
1623584_at	hkl	2L	35173	37B11-37B1	Dm.2091	-0,150692
1633846_at	CG15523	3R	43550	99C7-99C7	Dm.16311	-0,137668
1630841_at	CG8668	2L	34107	28E7-28E7	Dm.347	-0,146243
1634377_at	CG14646	3R	40555	82A5-82A5	Dm.7582	-0,134313
1624473_at	DNAprim	3L	44915	77B6-77B6	Dm.7051	-0,135831
1632194_at	Nup98	3R	42816	95B1-95B5	Dm.14811	-0,141988
1623825_s.	CG17018	2L	49895	40F7-40F7	Dm.20928	-0,140791
1633792_a.	osa	3R	42130	90C1-90C2	Dm.2989	-0,139097
1634122_at	CG31223	3R	326127	93A1-93A1	Dm.21119	-0,133725
1639411_at	l(1)G0148	X	31598	6C3-6C3	Dm.11300	-0,143502
1637547_at	CG8199	3R	41149	85D24-85D2	Dm.12640	-0,140765
1640300_at	CG33144	2R	36131	47B9-47B13	Dm.10908	-0,141982
1624632_at	Klp3A	X	31240	3A6-3A6	Dm.1693	-0,139801
1635755_a.	CG10741	3L	39521	70B2-70B3	Dm.15316	-0,136776
1624673_at	CG5899	2L	34311	30E1-30E4	Dm.3953	-0,15583
1627154_at	CG10904	2R	37817	60A14-60A1	Dm.6015	-0,144275
1627924_at	Rpl135	2L	33210	21C2-21C2	Dm.30753	-0,131261
1628011_a.	CG12877	3R	43333	98B2-98B2	Dm.14943	-0,143074
1631330_at	CG3408	3L	39107	67B10-67B1	Dm.4947	-0,14024
1627020_at	CG11110	2R	37337	57A8-57A8	Dm.15796	-0,142096
1628960_s.	CG17829	X	47718	1B12-1B12	Dm.10946	-0,140851
1640526_at	CG1103	3R	40547	82A4-82A4	Dm.1054	-0,154144
1635710_at	CG15470	X	31417	4D4-4D5	Dm.26460	0,128461
1628019_a.	spas	3R	42846	95D9-95D9	Dm.7035	-0,131623
1637085_at	Cdk5	2R	36727	52A13-52A1	Dm.640	-0,13377
1639599_at	CG4887	2L	33304	21F2-21F2	Dm.7247	-0,137676
1630805_at	noi	3R	40678	83B4-83B4	Dm.4519	-0,146763
1634172_at	TfIIB	2L	34430	31E2-31E2	Dm.4476	-0,126153
1640145_at	bys	X	31701	7C2-7C2	Dm.98	-0,141344
1624550_at	Trf	2L	34102	28E1-28E1	Dm.3090	-0,130861
1627280_s.	pnut	2R	35801	44C2-44C2	Dm.4132	-0,147348
1630741_s.	PGRP-LB	3R	41379	86E6-86E6	Dm.3374	-0,151754
1635473_at	Apf	2L	318908	30F1-30F1	Dm.24355	-0,137163
1627938_at	CG17840	2L	33658	24F4-24F4	Dm.297	-0,14303
1633364_at	CG14184	3L	40193	76E1-76E1	Dm.30734	-0,159735
1628731_s.	CG8080	2R	35918	45A2-45A2	Dm.20768	-0,148259
1636721_at	Pc	3L	40358	78C6-78C7	Dm.13644	-0,139954
1632816_at	Es2	X	31780	7E11-7E11	Dm.31045	-0,139769
1627131_at	spt4	2R	36387	49B10-49B1	Dm.19918	-0,158914
1627509_s.	hoe1	2L	249663	25B3-25B3	Dm.6487	-0,143759
1633767_at	Hrp59	3R	41138	85D18-85D1	Dm.7304	-0,133137
1636896_a.	CG12576	X	33135	20B3-20B3	Dm.6439	-0,149917
1641618_at	Gycalpha99E	3R	43493	99B7-99B7	Dm.3968	0,121746
1636073_at	CG32344	3L	326208	61C1-61C1	Dm.796	-0,14737
1628638_at	CG7200	2L	35089	36E6-36E6	Dm.7940	-0,142219
1627279_at	nwk	3L	39052	67A1-67A1	Dm.15455	0,126089
1624837_at	CG7506	3L	38857	66A8-66A8	Dm.3946	-0,138486
1631784_at	CG13663	3R	43029	96C5-96C5	Dm.25487	-0,128252
1640077_at	CalpC	X	32597	14B13-14B1	Dm.178	-0,136445
1634393_s.	Klp10A	X	32049	10A6-10A6	Dm.130	-0,139919
1627832_at	scat	2L	47942	30B3-30B3	Dm.1628	-0,150694
1628524_at	CG31008	3R	318554	100B1-100E	Dm.33607	0,137276

1623820_at	CG12252	2R	37925	60D5-60D5	Dm.10903	-0,133579
1637060_a.	Nopp140	3L	40433	78F4-78F4	Dm.3277	-0,141988
1623402_at	Arc-p34	2L	35311	38C6-38C6	Dm.4005	-0,151945
1628943_at	Tsf2	3L	39435	69C4-69C5	Dm.944	-0,15598
1636446_at	CSN6	3R	42661	94B5-94B5	Dm.2377	-0,13478
1632389_at	CG14812	X	31137	2B12-2B12	Dm.19624	-0,149714
1632956_at	CG3021	X	53546	1E1-1E1	Dm.23134	-0,148656
1639051_at	CG12938	2R	36141	47C1-47C1	Dm.30962	-0,153118
1631092_at	CG8032	3R	41026	85A5-85A5	Dm.1103	-0,129096
1632593_at	CG32147	3L	251690	71B5-71B5	Dm.6181	-0,14247
1623130_at	CG30457	2R	36940	54A2-54A2	Dm.15979	0,149833
1633643_a.	CG31033	3R	326115	99C2-99C2	Dm.20800	-0,131799
1624994_at	dup	2R	47121	51F11-51F1	Dm.1771	-0,138254
1637555_at	mtTFB1	3L	39320	68D2-68D2	Dm.29643	-0,134997
1639258_at	CG7335	3L	40203	76F2-76F2	Dm.31215	0,135556
1633466_at	CG7728	3L	39904	73E4-73E4	Dm.3597	-0,13654
1638014_at	CG5808	3R	42957	96A23-96A2	Dm.3212	-0,132057
1634182_at	CG11593	3L	38477	64A4-64A4	Dm.10937	-0,139898
1627209_at	mRNA-cappi	X	32379	12E5-12E5	Dm.5295	-0,138112
1623384_at	ytr	2R	47457	60A3-60A3	Dm.754	-0,132546
1638621_at	CG31648	2L	33776	25E5-25E5	Dm.33604	-0,139773
1640669_at	CG17163	X	3354988		Dm.6075	-0,133258
1640241_at	Tudor-SN	3L	38045	61B3-61B3	Dm.795	-0,146608
1626768_at	CG7044	3R	42497	93B11-93B1	Dm.6369	-0,1423
1631719_at	CG31109	3R	43001	96B19-96B1	Dm.6101	-0,153183
1622895_at	CG32075	3L	326192	68A3-68A4	Dm.925	-0,153278
1625755_at	CG1291	3L	38374	63B5-63B6	Dm.832	-0,145973
1630393_a.	Hrs	2L	33458	23A3-23A3	Dm.274	-0,118717
1629964_at	Nufip	3L	40043	75D8-75D8	Dm.3502	-0,134579
1641448_a.	Dg	2R	36773	52E2-52E4	Dm.1504	-0,129322
1634659_at	Dl	3R	42313	92A1-92A2	Dm.7687	-0,13377
1629281_at	Dhh1	3L	39195	67E3-67E3	Dm.921	-0,139077
1638575_at	Mcm6	X	31603	6C4-6C5	Dm.2944	-0,125753
1641449_at	Dcr-2	2R	36993	54C10-54C1	Dm.7764	-0,140272
1631534_at	sfl	3L	38736	65B3-65B4	Dm.4995	-0,125937
1626647_at	Mcm5	3R	41296	86C6-86C6	Dm.5829	-0,135793
1631125_at	CG15737	X	32152	10F4-10F4	Dm.6637	-0,13304
1627694_at	CG9715	3L	39883	73D1-73D1	Dm.6658	-0,133295
1625603_at	CG13604	3R	42840	95D5-95D5	Dm.4624	-0,11894
1634451_at	CG12236	X	31523	5C7-5C7	Dm.2138	-0,134038
1635102_at	syt	2L	33473	23A6-23B1	Dm.19478	0,143168
1623350_at	Lrr47	2L	34449	31F5-31F5	Dm.2776	-0,133246
1624168_at	CG14411	X	32408	12F5-12F5	Dm.10811	-0,131067
1629806_a.	CG32103	3L	39415	69B4-69B5	Dm.10857	-0,132151
1640568_at	CstF-64	3R	42239	91B8-91B8	Dm.4853	-0,127015
1623773_at	CG32082	3L	39258	68A8-68A9	Dm.21623	0,122876
1635551_at	CG8386	2R	36762	52D12-52D1	Dm.642	-0,129451
1624881_at	CG11448	X	31090	2A1-2A1	Dm.12	-0,134203
1638840_at	CG14641	3R	40529	82A1-82A1	Dm.3575	-0,128698
1628785_at	l(3)04053	3L	46158	80A1-80A1	Dm.4222	-0,135973
1626719_a.	wls	3L	39259	68A9-68A9	Dm.7728	-0,136873
1631006_a.	CG10171	3L	39501	70A7-70A8	Dm.952	-0,132844
1624252_s.	sle	3R	41262	86A8-86B1	Dm.4098	-0,144726

1631173_at	dob	X	32436	13A8-13A8	Dm.2112	-0,121529
1631696_s.	MAN1	2R	37838	60B5-60B5	Dm.7321	-0,148297
1637111_a.	PR2	2R	36442	49F3-49F4	Dm.4488	-0,132081
1631466_at	CG1523	3R	43400	98E5-98E5	Dm.4423	-0,133152
1638031_at	CG14512	3R	43443	98F12-98F1	Dm.31353	-0,134529
1640158_at	CG2608	2L	35323	38D2-38D2	Dm.3666	-0,136213
1636847_s.	Mkrn1	3L	44131	78D5-78D5	Dm.1642	-0,135618
1626883_at	CG1620	2R	35678	43C5-43C6	Dm.4394	-0,135963
1623967_at	Shc	3L	44052	67B5-67B5	Dm.1644	-0,142632
1630850_at	CG30469	2R	246631	52A6-52A6	Dm.26407	-0,133083
1624352_at	dpr13	2R	3885598	55A3-55A3	Dm.23935	0,130365
1639279_a.	Rlc1	3R	41141	85D19-85D'	Dm.20784	-0,14772
1626818_at	CG13097	2L	34184	29D3-29D4	Dm.359	-0,142074
1640758_at	CG32613	X	318117	12D1-12D1	Dm.6390	-0,126386
1633962_a.	CG14815	X	31141	2B13-2B13	Dm.11465	-0,134626
1638522_at	CG12123	X	31777	7E9-7E9	Dm.107	-0,115856
1639605_at	CG33993	3L	3885637	65A1-65A1	Dm.29571	0,139584
1627897_at	CG12265	3R	42077	89F1-89F1	Dm.1578	-0,127405
1627295_s.	CycD	X	32551	13F18-14A1	Dm.2096	-0,124135
1636164_s.	CG3107	2R	35475	41D3-41D3	Dm.13025	-0,123159
1624743_s.	PpD3	3R	49779	85E6-85E6	Dm.6984	-0,133317
1633860_at	CG12078	3L	38401	63C1-63C1	Dm.27812	0,132596
1623315_at	CG13253	3L	40288	77E3-77E3	Dm.18595	0,127617
1625898_s.	Lnk	3R	43130	96F6-96F6	Dm.7043	-0,119008
1634226_at	CG17249	3L	38201	62A9-62A9	Dm.11207	-0,12767
1632318_at	CG12773	X	31082	1F4-1F4	Dm.11524	-0,141231
1636002_at	CG4281	X	31171	2C8-2C8	Dm.3910	-0,126591
1624447_s.	dco	3R	43673	100B2-100E	Dm.1901	-0,145252
1630839_at	CG14804	X	31144	2B13-2B13	Dm.11603	-0,136185
1628241_at	CG2614	2L	35326	38D2-38D2	Dm.5004	-0,121883
1622930_a.	CG1957	3R	43426	98F5-98F5	Dm.1362	-0,142707
1627995_at	Art8	2L	34528	32C1-32C1	Dm.30827	-0,129999
1625386_at	da	2L	34413	31D11-31E1	Dm.388	-0,133041
1636939_at	Tango6	2L	35155	37B7-37B8	Dm.464	-0,133177
1628767_s.	MED26	4	43816	102C3-102(	Dm.18596	-0,128127
1632080_s.	Cpr67Fa1	3L	39223	67F1-67F1	Dm.7034	0,133253
1625944_a.	CG16838	3L	39770	72D9-72D9	Dm.11503	-0,126934
1637264_at	ncd	3R	43517	99C1-99C2	Dm.6668	-0,124781
1627636_at	CG14636	3R	40526	81F6-82A1	Dm.13649	0,126748
1637727_at	CG7049	3L	38022	61B2-61B2	Dm.793	-0,123099
1627087_at	CG14805	X	31146	2B14-2B14	Dm.11436	-0,139099
1624425_at	CG7384	2L	34447	31F4-31F4	Dm.393	-0,123566
1636809_at	Lam	2L	33782	25E6-25E6	Dm.312	-0,143364
1624145_a.	isopeptidase	2R	37261	56E1-56E1	Dm.9242	-0,133551
1636986_at	l(2)35Df	2L	48782	35D4-35D4	Dm.1748	-0,131639
1636651_a.	Csp	3L	40459	79D3-79D3	Dm.5140	-0,137549
1638290_at	CG32528	X	32990	18E5-18F1	Dm.11318	-0,11439
1629766_at	ca	3R	43518	99C2-99C2	Dm.6851	-0,124784
1630182_at	bin	3L	38766	65D6-65D6	Dm.5684	0,135235
1635129_at	CG6332	3R	42585	93F14-93F1	Dm.12009	0,120315
1633693_at	CG13814	3L	40223	77A4-77A4	Dm.27186	0,123837
1632109_s.	alphaCop	3L	38199	62A9-62A9	Dm.2217	-0,137825
1631577_a.	CG3760	2R	37987	60E5-60E5	Dm.6816	-0,130375

1641590_at	timeout	3R	41615	87D12-87D1	Dm.2433	-0,115026
1633166_at	CG12942	2R	36158	47C6-47C6	Dm.30902	-0,129576
1640805_at	CG13162	2R	36341	48F11-49A1	Dm.26909	-0,114896
1629016_s_	unc-13-4A	3L	38801	65E10-65E1	Dm.21296	0,134214
1639941_at	CG15317	X	31898	8E7-8E7	Dm.25382	-0,126051
1634784_at	yemalpha	3R	43439	98F10-98F1	Dm.21691	-0,136079
1641147_s_	CG3632	X	32589	14B9-14B9	Dm.6627	-0,126703
1632954_at	JTBR	3L	38235	62B4-62B4	Dm.24703	-0,119975
1629384_at	Brd8	3R	43460	99A1-99A1	Dm.20273	-0,125172
1624343_at	Opbp	2R	246618	42C2-42C2	Dm.14929	-0,117549
1641632_at	CG17048	2R	36479	50A3-50A3	Dm.26922	0,117081
1635901_at	CG17197	3R	43111	96F2-96F2	Dm.31346	0,12762
1638591_at	Tif-IA	2L	35454	40F6-40F7	Dm.485	-0,123768
1626106_a_	CG7139	3L	40448	79B2-79B2	Dm.3205	-0,120162
1625459_at	CG3520	2R	37708	59D10-59D1	Dm.14059	-0,115994

PValue	adjPVal
0,002688	0,079352
0,006081	0,079824
0,016108	0,090657
0,000235	0,079352
0,002673	0,079352
0,018002	0,093554
0,006106	0,079824
0,00014	0,079352
0,000334	0,079352
0,000246	0,079352
0,011946	0,08635
0,001014	0,079352
0,000231	0,079352
0,002483	0,079352
0,001432	0,079352
0,004105	0,079352
0,012466	0,086835
0,001401	0,079352
0,000405	0,079352
0,001571	0,079352
0,000309	0,079352
0,000386	0,079352
0,001158	0,079352
6,81E-05	0,079352
0,00073	0,079352
0,000232	0,079352
0,001172	0,079352
0,008722	0,082596
0,006297	0,079824
0,002038	0,079352
0,000153	0,079352
0,008075	0,082596
0,00053	0,079352
0,000809	0,079352
0,000649	0,079352
0,009425	0,082596
0,00173	0,079352
0,000586	0,079352
0,013611	0,087896
0,020576	0,096932
0,000151	0,079352
0,00145	0,079352
0,00377	0,079352
0,000874	0,079352
0,007354	0,081257
0,000239	0,079352
0,000381	0,079352
0,005548	0,079824
0,004512	0,079824
0,001579	0,079352
0,001068	0,079352
0,000257	0,079352

0,000112	0,079352
8,42E-05	0,079352
0,001527	0,079352
0,012974	0,087247
0,001091	0,079352
0,000185	0,079352
0,004037	0,079352
0,003721	0,079352
0,000443	0,079352
0,00265	0,079352
0,011424	0,085642
0,000549	0,079352
0,005334	0,079824
0,004269	0,079352
0,00479	0,079824
0,001566	0,079352
0,020841	0,096932
0,020559	0,096932
0,003216	0,079352
0,006261	0,079824
0,002441	0,079352
0,002386	0,079352
0,004145	0,079352
0,000235	0,079352
0,001121	0,079352
0,002422	0,079352
0,001123	0,079352
0,000813	0,079352
0,002933	0,079352
0,000716	0,079352
0,009171	0,082596
0,000893	0,079352
0,005043	0,079824
0,00408	0,079352
0,002385	0,079352
0,004394	0,079352
0,014742	0,089364
0,012769	0,086835
0,002493	0,079352
0,007314	0,081257
0,001935	0,079352
0,020499	0,096932
0,001709	0,079352
0,004119	0,079352
0,015084	0,089867
0,003119	0,079352
0,005582	0,079824
0,004386	0,079352
0,01473	0,089364
0,002447	0,079352
0,005606	0,079824
0,005386	0,079824
0,006231	0,079824

0,002484	0,079352
0,003583	0,079352
0,005366	0,079824
0,002063	0,079352
0,014123	0,088999
0,007537	0,082088
0,005473	0,079824
0,012135	0,086778
0,008352	0,082596
0,006708	0,08073
0,004072	0,079352
0,015297	0,089971
0,009048	0,082596
0,00892	0,082596
0,008804	0,082596
0,016961	0,091758
0,004123	0,079352
0,008207	0,082596
0,012749	0,086835
0,018799	0,094796
0,021704	0,098529
0,006026	0,079824
0,018083	0,093554
0,00732	0,081257
0,0077	0,082596
0,004041	0,079352
0,002574	0,079352
0,001949	0,079352
0,001288	0,079352
0,001691	0,079352
0,008658	0,082596
0,001396	0,079352
0,000177	0,079352
0,001553	0,079352
0,018347	0,094163
0,00116	0,079352
0,008205	0,082596
0,000754	0,079352
0,001889	0,079352
0,000316	0,079352
0,005034	0,079824
0,005312	0,079824
0,018687	0,094738
0,000425	0,079352
0,000971	0,079352
0,001874	0,079352
0,001849	0,079352
0,000881	0,079352
0,000207	0,079352
0,000357	0,079352
0,012915	0,087247
0,00434	0,079352
0,004162	0,079352

0,015869	0,090543
0,003359	0,079352
0,017398	0,092761
0,011998	0,08635
0,003202	0,079352
0,020388	0,096827
0,000419	0,079352
0,000414	0,079352
0,000512	0,079352
0,000322	0,079352
0,000968	0,079352
0,001274	0,079352
0,000308	0,079352
0,000474	0,079352
0,005682	0,079824
0,001067	0,079352
0,004689	0,079824
0,00702	0,081257
0,012614	0,086835
0,005304	0,079824
0,000782	0,079352
0,015887	0,090543
0,002619	0,079352
0,001646	0,079352
0,005943	0,079824
0,001214	0,079352
0,004273	0,079352
0,000367	0,079352
0,001162	0,079352
0,021994	0,098784
0,007221	0,081257
0,001977	0,079352
0,000818	0,079352
0,003818	0,079352
0,002637	0,079352
0,003881	0,079352
0,000625	0,079352
0,002912	0,079352
0,002859	0,079352
0,002228	0,079352
0,012102	0,086752
0,000963	0,079352
0,005379	0,079824
0,001352	0,079352
0,003484	0,079352
0,001821	0,079352
0,002795	0,079352
0,002707	0,079352
0,004569	0,079824
0,002027	0,079352
0,001062	0,079352
0,002422	0,079352
0,002886	0,079352

0,006247	0,079824
0,001871	0,079352
0,009475	0,082751
0,001702	0,079352
0,002007	0,079352
0,000539	0,079352
0,00092	0,079352
0,006566	0,079824
0,007503	0,081972
0,0004	0,079352
0,001998	0,079352
0,000756	0,079352
0,000954	0,079352
0,002422	0,079352
0,001817	0,079352
0,00541	0,079824
0,003196	0,079352
0,002055	0,079352
0,004278	0,079352
0,007224	0,081257
0,007248	0,081257
0,003793	0,079352
0,000583	0,079352
0,001285	0,079352
0,001225	0,079352
0,005056	0,079824
0,001619	0,079352
0,006482	0,079824
0,001498	0,079352
0,002025	0,079352
0,000499	0,079352
0,000834	0,079352
0,002035	0,079352
0,003128	0,079352
0,006853	0,081134
0,003385	0,079352
0,000889	0,079352
0,005567	0,079824
0,002841	0,079352
0,001385	0,079352
0,000832	0,079352
0,001089	0,079352
0,018016	0,093554
0,002566	0,079352
0,011454	0,085642
0,000887	0,079352
0,00101	0,079352
0,001621	0,079352
0,005368	0,079824
0,010068	0,083623
0,001119	0,079352
0,00167	0,079352
0,00242	0,079352

0,001044	0,079352
0,011588	0,085665
0,00226	0,079352
0,002357	0,079352
0,003366	0,079352
0,001814	0,079352
0,004396	0,079352
0,00166	0,079352
0,004553	0,079824
0,010678	0,084308
0,002496	0,079352
0,010837	0,084308
0,002762	0,079352
0,004117	0,079352
0,00534	0,079824
0,013673	0,088164
0,002041	0,079352
0,017125	0,09211
0,005177	0,079824
0,000664	0,079352
0,002931	0,079352
0,000771	0,079352
0,003206	0,079352
0,002992	0,079352
0,0022	0,079352
0,022762	0,099563
0,0041	0,079352
0,006456	0,079824
0,010243	0,08378
0,000583	0,079352
0,002055	0,079352
0,002495	0,079352
0,002898	0,079352
0,014149	0,089068
0,003947	0,079352
0,002198	0,079352
0,00356	0,079352
0,001581	0,079352
0,00095	0,079352
0,009088	0,082596
0,006007	0,079824
0,012602	0,086835
0,015915	0,090543
0,008875	0,082596
0,003678	0,079352
0,004874	0,079824
0,019077	0,094989
0,010684	0,084308
0,011587	0,085665
0,017692	0,093155
0,006849	0,081134
0,012429	0,086835
0,007052	0,081257

0,014363	0,089235
0,012207	0,086835
0,016263	0,090914
0,009249	0,082596
0,008194	0,082596
0,008266	0,082596
0,013425	0,087588
0,008106	0,082596
0,022056	0,098993
0,009652	0,082997
0,018672	0,094738
0,018939	0,094883
0,010754	0,084308
0,00851	0,082596
0,008825	0,082596
0,01606	0,090657
0,013144	0,08738
0,009035	0,082596
0,018319	0,094163
0,010577	0,084308
0,011907	0,08635
0,021733	0,098529
0,013446	0,087588
0,002023	0,079352
0,00284	0,079352
0,000885	0,079352
0,001776	0,079352
0,000938	0,079352
0,001356	0,079352
0,002753	0,079352
0,014433	0,089235
0,002942	0,079352
0,007019	0,081257
0,016529	0,091052
0,003201	0,079352
0,015156	0,089867
0,006115	0,079824
0,000904	0,079352
0,003627	0,079352
0,004098	0,079352
0,003579	0,079352
0,00951	0,082751
0,00359	0,079352
0,001944	0,079352
0,003919	0,079352
0,019176	0,095061
0,005621	0,079824
0,013989	0,088843
0,001854	0,079352
0,005882	0,079824
0,001169	0,079352
0,009381	0,082596
0,002188	0,079352

0,00682	0,081134
0,005217	0,079824
0,00158	0,079352
0,001669	0,079352
0,004593	0,079824
0,00554	0,079824
0,016293	0,091003
0,000774	0,079352
0,002943	0,079352
0,004981	0,079824
0,011653	0,085801
0,001772	0,079352
0,01067	0,084308
0,003552	0,079352
0,001786	0,079352
0,010594	0,084308
0,003361	0,079352
0,015178	0,089867
0,003224	0,079352
0,002776	0,079352
0,003623	0,079352
0,013044	0,087247
0,004705	0,079824
0,005996	0,079824
0,002699	0,079352
0,005731	0,079824
0,018553	0,094374
0,017635	0,093155
0,002201	0,079352
0,01902	0,094883
0,002591	0,079352
0,004013	0,079352
0,019014	0,094883
0,00522	0,079824
0,002271	0,079352
0,002587	0,079352
0,001971	0,079352
0,017362	0,092761
0,004889	0,079824
0,010752	0,084308
0,001022	0,079352
0,003553	0,079352
0,001899	0,079352
0,013824	0,088385
0,01062	0,084308
0,010262	0,083827
0,002034	0,079352
0,002163	0,079352
0,009258	0,082596
0,015046	0,089867
0,006394	0,079824
0,005551	0,079824
0,007809	0,082596

0,003225	0,079352
0,001658	0,079352
0,003558	0,079352
0,001675	0,079352
0,013417	0,087588
0,007514	0,081972
0,004138	0,079352
0,006037	0,079824
0,004426	0,079671
0,008136	0,082596
0,007089	0,081257
0,006839	0,081134
0,020754	0,096932
0,002632	0,079352
0,00202	0,079352
0,013742	0,088232
0,014983	0,089867
0,002224	0,079352
0,016111	0,090657
0,021569	0,098359
0,008451	0,082596
0,007964	0,082596
0,00239	0,079352
0,006407	0,079824
0,008612	0,082596
0,01145	0,085642
0,008785	0,082596
0,005664	0,079824
0,001624	0,079352
0,007117	0,081257
0,010092	0,083623
0,00187	0,079352
0,004273	0,079352
0,020935	0,096963
0,001921	0,079352
0,002632	0,079352
0,006028	0,079824
0,009435	0,082596
0,016102	0,090657
0,003065	0,079352
0,002304	0,079352
0,002228	0,079352
0,003356	0,079352
0,007848	0,082596
0,001389	0,079352
0,018034	0,093554
0,010212	0,083735
0,005653	0,079824
0,008086	0,082596
0,001878	0,079352
0,00552	0,079824
0,003789	0,079352
0,015173	0,089867

0,014547	0,089364
0,00623	0,079824
0,007784	0,082596
0,011042	0,084691
0,002072	0,079352
0,021545	0,098359
0,008725	0,082596
0,002988	0,079352
0,010791	0,084308
0,002656	0,079352
0,007043	0,081257
0,006297	0,079824
0,019	0,094883
0,004162	0,079352
0,014416	0,089235
0,006152	0,079824
0,002872	0,079352
0,006014	0,079824
0,00809	0,082596
0,00513	0,079824
0,002067	0,079352
0,006732	0,080867
0,008625	0,082596
0,01256	0,086835
0,003381	0,079352
0,011483	0,08566
0,003129	0,079352
0,004091	0,079352
0,003045	0,079352
0,008724	0,082596
0,010805	0,084308
0,001979	0,079352
0,010473	0,084308
0,019613	0,095836
0,003365	0,079352
0,022342	0,099296
0,004374	0,079352
0,012478	0,086835
0,007253	0,081257
0,004535	0,079824
0,003202	0,079352
0,003955	0,079352
0,008413	0,082596
0,007386	0,081257
0,004778	0,079824
0,001293	0,079352
0,002926	0,079352
0,003199	0,079352
0,004831	0,079824
0,0109	0,084528
0,02252	0,099372
0,003559	0,079352
0,015336	0,089971

0,001666	0,079352
0,008983	0,082596
0,003273	0,079352
0,002135	0,079352
0,007122	0,081257
0,002544	0,079352
0,003571	0,079352
0,022683	0,099389
0,006536	0,079824
0,007672	0,082596
0,014752	0,089364
0,014238	0,089103
0,005505	0,079824
0,017402	0,092761
0,003575	0,079352
0,01335	0,087588
0,002549	0,079352
0,005986	0,079824
0,002044	0,079352
0,002281	0,079352
0,015799	0,090543
0,003533	0,079352
0,001838	0,079352
0,00374	0,079352
0,008873	0,082596
0,005709	0,079824
0,003875	0,079352
0,020793	0,096932
0,00646	0,079824
0,005639	0,079824
0,004571	0,079824
0,003727	0,079352
0,002905	0,079352
0,003555	0,079352
0,00307	0,079352
0,006984	0,081257
0,003983	0,079352
0,019686	0,095836
0,004048	0,079352
0,001786	0,079352
0,005863	0,079824
0,007259	0,081257
0,012152	0,086788
0,008835	0,082596
0,010228	0,083761
0,008809	0,082596
0,002353	0,079352
0,01055	0,084308
0,004798	0,079824
0,009391	0,082596
0,006532	0,079824
0,01351	0,087588
0,020676	0,096932

0,00486	0,079824
0,005456	0,079824
0,002442	0,079352
0,005201	0,079824
0,01551	0,090255
0,012704	0,086835
0,010055	0,083623
0,003352	0,079352
0,005622	0,079824
0,018432	0,094327
0,001678	0,079352
0,016407	0,091052
0,00722	0,081257
0,004859	0,079824
0,008334	0,082596
0,01255	0,086835
0,020936	0,096963
0,009117	0,082596
0,004648	0,079824
0,009418	0,082596
0,010398	0,084308
0,016581	0,091052
0,004053	0,079352
0,019108	0,095031
0,004015	0,079352
0,009333	0,082596
0,020233	0,096466
0,00453	0,079824
0,009164	0,082596
0,00288	0,079352
0,021078	0,097375
0,007075	0,081257
0,004687	0,079824
0,006633	0,080086
0,005882	0,079824
0,014875	0,089614
0,006556	0,079824
0,004473	0,079824
0,004242	0,079352
0,0047	0,079824
0,00402	0,079352
0,003459	0,079352
0,004107	0,079352
0,008958	0,082596
0,010706	0,084308
0,006423	0,079824
0,004002	0,079352
0,021228	0,09754
0,01016	0,083623
0,00413	0,079352
0,003301	0,079352
0,011119	0,085142
0,005186	0,079824

0,008959	0,082596
0,007915	0,082596
0,003954	0,079352
0,010399	0,084308
0,004336	0,079352
0,006497	0,079824
0,005033	0,079824
0,017695	0,093155
0,009183	0,082596
0,005759	0,079824
0,003512	0,079352
0,002096	0,079352
0,010138	0,083623
0,005278	0,079824
0,006551	0,079824
0,012676	0,086835
0,019238	0,095169
0,008185	0,082596
0,007185	0,081257
0,002582	0,079352
0,008991	0,082596
0,01694	0,091716
0,003278	0,079352
0,017391	0,092761
0,002296	0,079352
0,005167	0,079824
0,010098	0,083623
0,013037	0,087247
0,012494	0,086835
0,014286	0,089169
0,00387	0,079352
0,012387	0,086835
0,00688	0,081161
0,0068	0,081134
0,01327	0,087588
0,018469	0,094368
0,006624	0,080086
0,013058	0,087254
0,004306	0,079352
0,006895	0,081188
0,005288	0,079824
0,009585	0,082853
0,019975	0,095974
0,006559	0,079824
0,003339	0,079352
0,007769	0,082596
0,015121	0,089867
0,002643	0,079352
0,011429	0,085642
0,003134	0,079352
0,00591	0,079824
0,005275	0,079824
0,018102	0,093554

0,014752	0,089364
0,00899	0,082596
0,003461	0,079352
0,006547	0,079824
0,004454	0,079824
0,022122	0,098993
0,012137	0,086778
0,003614	0,079352
0,01472	0,089364
0,00644	0,079824
0,004548	0,079824
0,018023	0,093554
0,015095	0,089867
0,007932	0,082596
0,014263	0,089109
0,00551	0,079824
0,003977	0,079352
0,006228	0,079824
0,013823	0,088385
0,00568	0,079824
0,009617	0,082943
0,00465	0,079824
0,002934	0,079352
0,006786	0,081134
0,014545	0,089364
0,010782	0,084308
0,00638	0,079824
0,004748	0,079824
0,015371	0,089971
0,009539	0,082803
0,003096	0,079352
0,006569	0,079824
0,004892	0,079824
0,009509	0,082751
0,010115	0,083623
0,004201	0,079352
0,003225	0,079352
0,003943	0,079352
0,005333	0,079824
0,013252	0,087588
0,007384	0,081257
0,002667	0,079352
0,002827	0,079352
0,005802	0,079824
0,004144	0,079352
0,004627	0,079824
0,003066	0,079352
0,003676	0,079352
0,005253	0,079824
0,011232	0,085301
0,009226	0,082596
0,016438	0,091052
0,004154	0,079352

0,003934	0,079352
0,01337	0,087588
0,00531	0,079824
0,010562	0,084308
0,019863	0,095936
0,00354	0,079352
0,018748	0,094757
0,010372	0,084308
0,004364	0,079352
0,003219	0,079352
0,020635	0,096932
0,009893	0,083623
0,003988	0,079352
0,011239	0,085301
0,005488	0,079824
0,005482	0,079824
0,005136	0,079824
0,008425	0,082596
0,010279	0,083862
0,01015	0,083623
0,010011	0,083623
0,006451	0,079824
0,007205	0,081257
0,007003	0,081257
0,00478	0,079824
0,006058	0,079824
0,012468	0,086835
0,010049	0,083623
0,004544	0,079824
0,003852	0,079352
0,007435	0,081524
0,012661	0,086835
0,003792	0,079352
0,008761	0,082596
0,011685	0,085801
0,008413	0,082596
0,005318	0,079824
0,010146	0,083623
0,005343	0,079824
0,005963	0,079824
0,006338	0,079824
0,00457	0,079824
0,004684	0,079824
0,006154	0,079824
0,022336	0,099296
0,017309	0,092761
0,005789	0,079824
0,013414	0,087588
0,008623	0,082596
0,009367	0,082596
0,015853	0,090543
0,005636	0,079824
0,011864	0,08635

0,00919	0,082596
0,009621	0,082943
0,006084	0,079824
0,018328	0,094163
0,005034	0,079824
0,01273	0,086835
0,014371	0,089235
0,00988	0,083623
0,010096	0,083623
0,015016	0,089867
0,008267	0,082596
0,009714	0,083029
0,008826	0,082596
0,004131	0,079352
0,020851	0,096932
0,007784	0,082596
0,011967	0,08635
0,008945	0,082596
0,022081	0,098993
0,010888	0,084528
0,013836	0,088385
0,010949	0,084528
0,017878	0,093391
0,016147	0,090731
0,01607	0,090657
0,006642	0,080086
0,004899	0,079824
0,021376	0,097994
0,004718	0,079824
0,00342	0,079352
0,011322	0,085301
0,005884	0,079824
0,0102	0,083735
0,00855	0,082596
0,004138	0,079352
0,01357	0,087891
0,006609	0,080086
0,013795	0,088385
0,009413	0,082596
0,007238	0,081257
0,017681	0,093155
0,007889	0,082596
0,004925	0,079824
0,013106	0,08738
0,006039	0,079824
0,003981	0,079352
0,00656	0,079824
0,018722	0,094749
0,006517	0,079824
0,009078	0,082596
0,004801	0,079824
0,004289	0,079352
0,01385	0,088389

0,003935	0,079352
0,008353	0,082596
0,011508	0,085665
0,01118	0,085161
0,015989	0,090657
0,018851	0,094839
0,009281	0,082596
0,008149	0,082596
0,006145	0,079824
0,012463	0,086835
0,018951	0,094883
0,005661	0,079824
0,008293	0,082596
0,011583	0,085665
0,014101	0,088999
0,009316	0,082596
0,01591	0,090543
0,004255	0,079352
0,005028	0,079824
0,012607	0,086835
0,007286	0,081257
0,009433	0,082596
0,007276	0,081257
0,019725	0,095836
0,006572	0,079824
0,016551	0,091052
0,008014	0,082596
0,012849	0,087105
0,00644	0,079824
0,004235	0,079352
0,007227	0,081257
0,014789	0,089364
0,02224	0,099273
0,005013	0,079824
0,004338	0,079352
0,010792	0,084308
0,017466	0,092871
0,011684	0,085801
0,005382	0,079824
0,004739	0,079824
0,016898	0,091667
0,004112	0,079352
0,009412	0,082596
0,020539	0,096932
0,017035	0,091928
0,00519	0,079824
0,009269	0,082596
0,006062	0,079824
0,020627	0,096932
0,012804	0,086983
0,013326	0,087588
0,007814	0,082596
0,014364	0,089235

0,007407	0,081352
0,011275	0,085301
0,008468	0,082596
0,019921	0,095936
0,009303	0,082596
0,017363	0,092761
0,004075	0,079352
0,016797	0,09155
0,00473	0,079824
0,008319	0,082596
0,007097	0,081257
0,010126	0,083623
0,012964	0,087247
0,004948	0,079824
0,021049	0,097349
0,007244	0,081257
0,00864	0,082596
0,008154	0,082596
0,010946	0,084528
0,022271	0,099296
0,011889	0,08635
0,011472	0,08566
0,014943	0,089835
0,014539	0,089364
0,004316	0,079352
0,01019	0,083735
0,007994	0,082596
0,022554	0,099376
0,014597	0,089364
0,007326	0,081257
0,022143	0,098993
0,01665	0,091052
0,014895	0,08963
0,012637	0,086835
0,013884	0,088431
0,012645	0,086835
0,007349	0,081257
0,008695	0,082596
0,006006	0,079824
0,010116	0,083623
0,012493	0,086835
0,014114	0,088999
0,005795	0,079824
0,008103	0,082596
0,008093	0,082596
0,016506	0,091052
0,018817	0,094809
0,009375	0,082596
0,008822	0,082596
0,010394	0,084308
0,004226	0,079352
0,005807	0,079824
0,014351	0,089235

0,019717	0,095836
0,022682	0,099389
0,006826	0,081134
0,022454	0,099372
0,006501	0,079824
0,016728	0,091327
0,005706	0,079824
0,017712	0,093155
0,022146	0,098993
0,012101	0,086752
0,007796	0,082596
0,007083	0,081257
0,015037	0,089867
0,008413	0,082596
0,007385	0,081257
0,010655	0,084308
0,010308	0,083991
0,010933	0,084528
0,004696	0,079824
0,020801	0,096932
0,009746	0,083039
0,012688	0,086835
0,021808	0,098714
0,020071	0,096261
0,019594	0,095836
0,011152	0,085161
0,016817	0,091582
0,009106	0,082596
0,012505	0,086835
0,005296	0,079824
0,018676	0,094738
0,009385	0,082596
0,014753	0,089364
0,005526	0,079824
0,009558	0,082803
0,006937	0,081257
0,016362	0,091052
0,006875	0,081161
0,009232	0,082596
0,014621	0,089364
0,009153	0,082596
0,007825	0,082596
0,005619	0,079824
0,020715	0,096932
0,014052	0,088981
0,021398	0,097994
0,012447	0,086835
0,006419	0,079824
0,010576	0,084308
0,006319	0,079824
0,018109	0,093554
0,015759	0,090543
0,006063	0,079824

0,01117	0,085161
0,015491	0,090226
0,010808	0,084308
0,021741	0,098529
0,012367	0,086835
0,00929	0,082596
0,008218	0,082596
0,00755	0,082096
0,01658	0,091052
0,007347	0,081257
0,007585	0,082207
0,016499	0,091052
0,013135	0,08738
0,009777	0,083083
0,013496	0,087588
0,022633	0,099389
0,018335	0,094163
0,015317	0,089971
0,014664	0,089364
0,011285	0,085301
0,015174	0,089867
0,021726	0,098529
0,008821	0,082596
0,008181	0,082596
0,010159	0,083623
0,015386	0,089971
0,01481	0,089364
0,022805	0,099687
0,008228	0,082596
0,007665	0,082596
0,007702	0,082596
0,01126	0,085301
0,005638	0,079824
0,022567	0,099376
0,015358	0,089971
0,007583	0,082207
0,010461	0,084308
0,013725	0,088232
0,016507	0,091052
0,008893	0,082596
0,018268	0,094148
0,014106	0,088999
0,008805	0,082596
0,01014	0,083623
0,010931	0,084528
0,010741	0,084308
0,006312	0,079824
0,008485	0,082596
0,006172	0,079824
0,01539	0,089971
0,009547	0,082803
0,005718	0,079824
0,008177	0,082596

0,011404	0,085642
0,022429	0,099372
0,011126	0,085142
0,005622	0,079824
0,012674	0,086835
0,009326	0,082596
0,015367	0,089971
0,013033	0,087247
0,011311	0,085301
0,020657	0,096932
0,008006	0,082596
0,013693	0,088164
0,012696	0,086835
0,016553	0,091052
0,015875	0,090543
0,018017	0,093554
0,008462	0,082596
0,009161	0,082596
0,016628	0,091052
0,010047	0,083623
0,015527	0,090277
0,009342	0,082596
0,007138	0,081257
0,010016	0,083623
0,015627	0,090543
0,022106	0,098993
0,015201	0,089897
0,019721	0,095836
0,010837	0,084308
0,009255	0,082596
0,008087	0,082596
0,008879	0,082596
0,012453	0,086835
0,00967	0,083029
0,013965	0,088772
0,007117	0,081257
0,008608	0,082596
0,014879	0,089614
0,017571	0,093126
0,012526	0,086835
0,015851	0,090543
0,012107	0,086752
0,013351	0,087588
0,016355	0,091052
0,015769	0,090543
0,010752	0,084308
0,009839	0,0835
0,006796	0,081134
0,020927	0,096963
0,011625	0,085801
0,016081	0,090657
0,009725	0,083029
0,016796	0,09155

0,012204	0,086835
0,022354	0,099296
0,007252	0,081257
0,012326	0,086835
0,006484	0,079824
0,015115	0,089867
0,008621	0,082596
0,007463	0,081688
0,012358	0,086835
0,01954	0,095726
0,011544	0,085665
0,008293	0,082596
0,021086	0,097375
0,011955	0,08635
0,014076	0,088999
0,011783	0,086262
0,00976	0,083048
0,005515	0,079824
0,011896	0,08635
0,011008	0,084636
0,017846	0,093374
0,007939	0,082596
0,019739	0,095836
0,020493	0,096932
0,022914	0,099963
0,009646	0,082997
0,010023	0,083623
0,013606	0,087896
0,01324	0,087588
0,011313	0,085301
0,019703	0,095836
0,015182	0,089867
0,014162	0,089068
0,019675	0,095836
0,017987	0,093554
0,008983	0,082596
0,010506	0,084308
0,021611	0,098418
0,015269	0,089971
0,014405	0,089235
0,008162	0,082596
0,00608	0,079824
0,017778	0,093165
0,013693	0,088164
0,021558	0,098359
0,01888	0,094839
0,015218	0,08992
0,016199	0,090791
0,010008	0,083623
0,013003	0,087247
0,022473	0,099372
0,016536	0,091052
0,010155	0,083623

0,013249	0,087588
0,01774	0,093155
0,014535	0,089364
0,00724	0,081257
0,009707	0,083029
0,017402	0,092761
0,014378	0,089235
0,006467	0,079824
0,013313	0,087588
0,022691	0,099389
0,018538	0,094374
0,019015	0,094883
0,008114	0,082596
0,020271	0,096554
0,011665	0,085801
0,011967	0,08635
0,021906	0,098714
0,017765	0,093165
0,016022	0,090657
0,017113	0,09211
0,014782	0,089364
0,010055	0,083623
0,015654	0,090543
0,01133	0,085301
0,009385	0,082596
0,009067	0,082596
0,013473	0,087588
0,019954	0,095974
0,011575	0,085665
0,015862	0,090543
0,01782	0,093311
0,01622	0,090832
0,010507	0,084308
0,019988	0,095974
0,013163	0,087419
0,011235	0,085301
0,01303	0,087247
0,01612	0,090657
0,016899	0,091667
0,015729	0,090543
0,014798	0,089364
0,020843	0,096932
0,017697	0,093155
0,008543	0,082596
0,010812	0,084308
0,011971	0,08635
0,010984	0,084543
0,021933	0,098714
0,011026	0,08467
0,008938	0,082596
0,012232	0,086835
0,015691	0,090543
0,010426	0,084308

0,012252	0,086835
0,010968	0,084528
0,009277	0,082596
0,019658	0,095836
0,014558	0,089364
0,009441	0,082596
0,018405	0,094262
0,008314	0,082596
0,011812	0,086347
0,013461	0,087588
0,015919	0,090543
0,017456	0,092871
0,013745	0,088232
0,008977	0,082596
0,008304	0,082596
0,014578	0,089364
0,020303	0,096588
0,015708	0,090543
0,013468	0,087588
0,020552	0,096932
0,007809	0,082596
0,008085	0,082596
0,010785	0,084308
0,015637	0,090543
0,019541	0,095726
0,012678	0,086835
0,009732	0,083029
0,019283	0,095169
0,016718	0,091327
0,008586	0,082596
0,00821	0,082596
0,022405	0,099372
0,013223	0,087588
0,016366	0,091052
0,019274	0,095169
0,010647	0,084308
0,020077	0,096261
0,015128	0,089867
0,016092	0,090657
0,011566	0,085665
0,010593	0,084308
0,010675	0,084308
0,012637	0,086835
0,014222	0,089103
0,013775	0,088344
0,012427	0,086835
0,01691	0,091667
0,02251	0,099372
0,009213	0,082596
0,009727	0,083029
0,016917	0,091667
0,017723	0,093155
0,018714	0,094749

0,010753	0,084308
0,019159	0,095061
0,011399	0,085642
0,021889	0,098714
0,022511	0,099372
0,011913	0,08635
0,015675	0,090543
0,013501	0,087588
0,017518	0,093049
0,011583	0,085665
0,016617	0,091052
0,009501	0,082751
0,016315	0,091048
0,01935	0,095294
0,012914	0,087247
0,0188	0,094796
0,020308	0,096588
0,014429	0,089235
0,013433	0,087588
0,021157	0,097492
0,02079	0,096932
0,020217	0,096466
0,019085	0,094989
0,013387	0,087588
0,015734	0,090543
0,018189	0,093817
0,009566	0,082803
0,008022	0,082596
0,016597	0,091052
0,012472	0,086835
0,012066	0,086742
0,017627	0,093155
0,016001	0,090657
0,012571	0,086835
0,022551	0,099376
0,017386	0,092761
0,018797	0,094796
0,012763	0,086835
0,011992	0,08635
0,020616	0,096932
0,021831	0,098714
0,015409	0,089971
0,020367	0,096798
0,022359	0,099296
0,022626	0,099389
0,011964	0,08635
0,020888	0,096963
0,018129	0,09358
0,015785	0,090543
0,017029	0,091928
0,015979	0,090657
0,01767	0,093155
0,01542	0,089971

0,010548	0,084308
0,020639	0,096932
0,014639	0,089364
0,011655	0,085801
0,015356	0,089971
0,018539	0,094374
0,011181	0,085161
0,018527	0,094374
0,010969	0,084528
0,008769	0,082596
0,012968	0,087247
0,009006	0,082596
0,0156	0,090543
0,019131	0,095061
0,013119	0,08738
0,016983	0,091797
0,011788	0,086262
0,013608	0,087896
0,014473	0,089311
0,011779	0,086262
0,022758	0,099563
0,013035	0,087247
0,017747	0,093155
0,021202	0,09754
0,021697	0,098529
0,014015	0,088918
0,013043	0,087247
0,012527	0,086835
0,014453	0,089271
0,013127	0,08738
0,021161	0,097492
0,016235	0,090835
0,019856	0,095936
0,01479	0,089364
0,017639	0,093155
0,022669	0,099389
0,017542	0,093049
0,014248	0,089103
0,012765	0,086835
0,020231	0,096466
0,022303	0,099296
0,012598	0,086835
0,022671	0,099389
0,020413	0,096876
0,016403	0,091052
0,020579	0,096932
0,019642	0,095836
0,013933	0,088658
0,015039	0,089867
0,018881	0,094839
0,016469	0,091052
0,019763	0,095881
0,013883	0,088431

0,013427	0,087588
0,022366	0,099296
0,02078	0,096932
0,020782	0,096932
0,014178	0,089068
0,017352	0,092761
0,014188	0,089068
0,022834	0,099745
0,01714	0,092115
0,015456	0,090105
0,01809	0,093554
0,014035	0,088961
0,012649	0,086835
0,01894	0,094883
0,020932	0,096963
0,022136	0,098993
0,015815	0,090543
0,018732	0,094749
0,012883	0,087246
0,021497	0,098358
0,017458	0,092871
0,020663	0,096932
0,01501	0,089867
0,020855	0,096932
0,019044	0,094929
0,021239	0,09754
0,015949	0,090631
0,019397	0,09545
0,020726	0,096932
0,020984	0,097117
0,020153	0,096466
0,015047	0,089867
0,022194	0,099139
0,019256	0,095169
0,019823	0,095936
0,019477	0,095722
0,018092	0,093554
0,012821	0,087007
0,021099	0,097375
0,016614	0,091052
0,019539	0,095726
0,017871	0,093391
0,015409	0,089971
0,016172	0,090744
0,012608	0,086835
0,018495	0,094374
0,019481	0,095722
0,021277	0,097644
0,011891	0,08635
0,014752	0,089364
0,016643	0,091052
0,018028	0,093554
0,017098	0,09211

0,020742	0,096932
0,019973	0,095974
0,016177	0,090744
0,013257	0,087588
0,01423	0,089103
0,014644	0,089364
0,021952	0,09873
0,019894	0,095936
0,018557	0,094374
0,015365	0,089971
0,015727	0,090543
0,022678	0,099389
0,016892	0,091667
0,020233	0,096466
0,019863	0,095936
0,020146	0,096466
0,021523	0,098358
0,018858	0,094839
0,020574	0,096932
0,020853	0,096932
0,019885	0,095936
0,019819	0,095936
0,021925	0,098714
0,020238	0,096466
0,015685	0,090543
0,021908	0,098714
0,020238	0,096466
0,021714	0,098529
0,016598	0,091052
0,02199	0,098784
0,018903	0,094879
0,016604	0,091052
0,021692	0,098529
0,019187	0,095061
0,018361	0,094163
0,018621	0,094625
0,014661	0,089364
0,018372	0,094163
0,014419	0,089235
0,017533	0,093049
0,019711	0,095836
0,018457	0,094368
0,021513	0,098358
0,018086	0,093554
0,022867	0,099822
0,018071	0,093554
0,021583	0,098359
0,019157	0,095061
0,017073	0,092058
0,021395	0,097994
0,019311	0,095169
0,021869	0,098714
0,016916	0,091667

0,019017	0,094883
0,019303	0,095169
0,021821	0,098714
0,019284	0,095169
0,017381	0,092761
0,022418	0,099372
0,0195	0,095726
0,019909	0,095936
0,022321	0,099296
0,021928	0,098714
0,018371	0,094163
0,019801	0,095936
0,022515	0,099372
0,021225	0,09754
0,021169	0,097492
