# Supplementary material for: Female Drosophila melanogaster Gene Expression and Mate Choice: The X Chromosome Harbours Candidate Genes Underlying Sexual Isolation
Source: PLoS One. 2011 Feb 28;6(2):e17358. doi: 10.1371/journal.pone.0017358 (PMC3046225; doi:10.1371/journal.pone.0017358)
Supplement: Table S2 — Tissue-level enrichment analysis. Tissues with an overrepresentation of differentially expressed genes with at least 200% the expression level in whole flies (P<0.05) are highlighted in bold. (PDF) [file pone.0017358.s003.pdf]

Table S2. Tissue-level enrichment analysis. Tissues with an overrepresentation of differentially expressed genes with at least 200% the expression level in whole flies (P < 0.05) are highlighted in bold.

Tissue	P value	Odds ratio	Expected count	Count	Total genes
**Brain**	**0.015**	**1.164**	**284.47**	**331**	**1241**
Head	1	0.382	264.76	101	1155
Eye	1	0.662	244.59	162	1067
Thoracic abdominal ganglion	0.277	1.046	273.47	286	1193
Salivary gland	0.999	0.773	312.9	242	1365
Crop	0.999	0.8	232.21	181	1013
Midgut	1	0.541	240.46	130	1049
Tubule	1	0.687	218.45	150	953
Hindgut	1	0.547	217.54	119	949
heart	1	0.529	215.47	114	940
Fat body	1	0.631	237.71	150	1037
**Ovary**	**2.20E-16**	**2.134**	**300.29**	**641**	**1310**
Virgin spermatheca	1	0.645	226.25	146	987
Mated spermatheca	1	0.639	233.12	149	1017
Carcass	1	0.316	275.53	87	1202
