# Supplementary material for: Female Drosophila melanogaster Gene Expression and Mate Choice: The X Chromosome Harbours Candidate Genes Underlying Sexual Isolation
Source: PLoS One. 2011 Feb 28;6(2):e17358. doi: 10.1371/journal.pone.0017358 (PMC3046225; doi:10.1371/journal.pone.0017358)
Supplement: Table S3 — Results of non-random chromosomal distribution analysis, including all significant cytobands, sub-bands and sub-sub bands (p<0.05). (PDF) [file pone.0017358.s004.pdf]

Table S3. Results of non-random chromosomal distribution analysis, including all significant cytobands, sub-bands and sub-sub bands ($p < 0.05$).

Chromosome Map ID	P value	Odds Ratio	Expected Count	Count	Size
X	2.24E-08	1.475	241.371	317	2079
47C	5.64E-04	4.503	3.135	10	27
72C	7.74E-04	6.689	1.741	7	15
31E2	8.21E-04	30.536	0.58	4	5
38D2	8.67E-04	12.73	0.929	5	8
79D	8.67E-04	12.73	0.929	5	8
61C	1.11E-03	3.404	4.528	12	39
79D3	1.56E-03	Inf	0.348	3	3
96B20	2.24E-03	15.266	0.697	4	6
61	2.84E-03	1.939	15.557	27	134
61C8	3.18E-03	5.73	1.625	6	14
1	4.40E-03	1.983	13.003	23	112
47C5	4.74E-03	10.177	0.813	4	7
96B19	4.74E-03	10.177	0.813	4	7
98E5	4.74E-03	10.177	0.813	4	7
1F	5.30E-03	6.363	1.277	5	11
93B	5.69E-03	3.597	2.902	8	25
1B13	5.71E-03	22.886	0.464	3	4
24C8	5.71E-03	22.886	0.464	3	4
33B11	5.71E-03	22.886	0.464	3	4
54E9	5.71E-	22.886	0.464	3	4

	03				
57F4	5.71E-03	22.886	0.464	3	4
38D	6.92E-03	4.583	1.858	6	16
61B	6.99E-03	3.128	3.599	9	31
13F	7.47E-03	3.82	2.438	7	21
47C1	8.22E-03	5.454	1.393	5	12
72C1	8.22E-03	5.454	1.393	5	12
22D1	8.61E-03	7.632	0.929	4	8
27C4	8.61E-03	7.632	0.929	4	8
2B	1.04E-02	2.416	5.805	12	50
1F4	1.30E-02	11.442	0.58	3	5
64D5	1.30E-02	11.442	0.58	3	5
66B7	1.30E-02	11.442	0.58	3	5
79A4	1.30E-02	11.442	0.58	3	5
10C6	1.35E-02	Inf	0.232	2	2
10E6	1.35E-02	Inf	0.232	2	2
12C1	1.35E-02	Inf	0.232	2	2
45A7	1.35E-02	Inf	0.232	2	2
48F8	1.35E-02	Inf	0.232	2	2
4B3	1.35E-02	Inf	0.232	2	2
51E2	1.35E-02	Inf	0.232	2	2
56C1	1.35E-02	Inf	0.232	2	2
70E5	1.35E-02	Inf	0.232	2	2
72C2	1.35E-	Inf	0.232	2	2

	02				
87D11	1.35E-02	Inf	0.232	2	2
8F2	1.35E-02	Inf	0.232	2	2
92B9	1.35E-02	Inf	0.232	2	2
95F12	1.35E-02	Inf	0.232	2	2
56F16	1.41E-02	6.105	1.045	4	9
7	1.70E-02	1.681	16.138	25	139
28E	1.71E-02	4.241	1.625	5	14
29C	1.73E-02	3.524	2.206	6	19
72	1.79E-02	1.738	13.816	22	119
2	1.82E-02	1.711	14.629	23	126
14	2.11E-02	1.859	10.101	17	87
5E	2.23E-02	3.272	2.322	6	20
7E	2.23E-02	3.272	2.322	6	20
61C1	2.32E-02	3.816	1.741	5	15
18D13	2.38E-02	7.627	0.697	3	6
38A2	2.38E-02	7.627	0.697	3	6
48E2	2.38E-02	7.627	0.697	3	6
52A6	2.38E-02	7.627	0.697	3	6
70C5	2.38E-02	7.627	0.697	3	6
72D9	2.38E-02	7.627	0.697	3	6
88A3	2.38E-02	7.627	0.697	3	6
92E2	2.38E-02	7.627	0.697	3	6
94E	2.53E-	2.814	3.019	7	26

	02				
7F	2.83E-02	3.054	2.438	6	21
35D4	3.05E-02	4.36	1.277	4	11
61B2	3.06E-02	3.469	1.858	5	16
18D	3.08E-02	2.673	3.135	7	27
96B	3.39E-02	2.051	6.037	11	52
30B	3.52E-02	2.863	2.554	6	22
31E	3.52E-02	2.863	2.554	6	22
10	3.53E-02	1.624	13.932	21	120
18D11	3.73E-02	15.246	0.348	2	3
1F3	3.73E-02	15.246	0.348	2	3
23C3	3.73E-02	15.246	0.348	2	3
26B5	3.73E-02	15.246	0.348	2	3
28E1	3.73E-02	15.246	0.348	2	3
29C5	3.73E-02	15.246	0.348	2	3
29E4	3.73E-02	15.246	0.348	2	3
33B9	3.73E-02	15.246	0.348	2	3
37B10	3.73E-02	15.246	0.348	2	3
43E3	3.73E-02	15.246	0.348	2	3
57C2	3.73E-02	15.246	0.348	2	3
58D8	3.73E-02	15.246	0.348	2	3
5E4	3.73E-02	15.246	0.348	2	3
60B10	3.73E-02	15.246	0.348	2	3
72B	3.73E-	15.246	0.348	2	3

	02				
76C5	3.73E-02	15.246	0.348	2	3
7A	3.73E-02	15.246	0.348	2	3
93B13	3.73E-02	15.246	0.348	2	3
94E13	3.73E-02	15.246	0.348	2	3
96F6	3.73E-02	15.246	0.348	2	3
16F6	3.81E-02	5.72	0.813	3	7
67B5	3.81E-02	5.72	0.813	3	7
16B	3.93E-02	3.18	1.974	5	17
33B	4.31E-02	2.694	2.67	6	23
56F	4.37E-02	2.263	4.063	8	35
18	4.54E-02	1.639	11.842	18	102
18C	4.94E-02	2.935	2.09	5	18
